# ﻿A revision of the hitherto neglected genus *Topiris* Walker, 1863 (Lepidoptera, Xyloryctidae) with taxonomic notes on the genus *Athrypsiastis* Meyrick, 1910

**DOI:** 10.3897/zookeys.1229.119155

**Published:** 2025-03-04

**Authors:** Mark J. Sterling, Ben W. Price, David C. Lees

**Affiliations:** 1 Department of Science, Natural History Museum, Cromwell Road, South Kensington, London SW7 5BD, UK Natural History Museum London United Kingdom

**Keywords:** Alfred Russel Wallace, DNA barcodes, generic revision, museomics, new species, pest species, third generation sequencing

## Abstract

The genus *Topiris* Walker, 1863 is revised. This genus, previously neglected or deemed unrecognisable, comprised only Walker’s damaged and misrepaired type specimen of *Topiriscandidella* Walker, 1863. Evidence is provided that this specimen was collected by Alfred Russel Wallace in 1855–56 in Sarawak, Malaysian Borneo. The mitogenome of this specimen was assembled using low coverage whole genome sequencing (genome skimming). The COI-5P portion of this mitogenome (658 bp) differs by 1–3 bp from two haplotypes sequenced from early 1990’s Brunei specimens. Another specimen recently discovered at NHMUK with an identical label to that of the type perfectly matches the Brunei specimens in its genitalia. Based on these four specimens, we present a fuller description of the morphology of *T.candidella*. *Topiris* includes the following additional species authored by Sterling and Lees: *Topirisalbidella***sp. nov.**, *T.albogrisella***sp. nov.**, *T.cinderella***sp. nov.**, *T.digiticosta***sp. nov.**, *T.lacteella***sp. nov.**, *T.madonna***sp. nov.**, *T.meyricki***sp. nov.**, *T.ochrotincta***sp. nov.**, *T.schneeweissella***sp. nov.**, *T.sericella***sp. nov.**, and *T.thunbergella***sp. nov.** The following new combinations are also established: *T.salva* (Meyrick, 1932), **comb. nov.** and *T.sampitella* (Lvovsky, 2014), **comb. nov.** The type of *Athrypsiastissalva* Meyrick is confirmed as lost and so a neotype and paraneotype of this species are designated. A published mitogenome of “*Linoclostisgonatias*” is shown to be correctly identified as *T.salva*, and references to *L.gonatias*, identified in some literature as a pest of Theaceae, are likely misidentified. The genus *Topiris* is divided into three groups, the *candidella* group, the *salva* group, and the *albidella* group, based on characters in the male genitalia. The *candidella* group and *albidella* group are supported sub-clades of *Topiris*. The phylogenetic placement of *Topiris* and *Athrypsiastis* within ‘core’ Xyloryctidae (as subtended by its type species, *X.luteotactella*) is confirmed by analysis of COI and seven nuclear genes, whereas the genera *Eumenodora* Meyrick, 1906 and *Izatha* Walker, 1864 do not fall within this clade. The morphology of *Athrypsiastisphaeoleuca* Meyrick, 1910 (the type species of *Athrypsiastis*; Xyloryctidae) is more fully described. The following new species authored by Sterling and Lees are described: *Athrypsiastischeesmanae***sp. nov.**, *A.edelweissella***sp. nov.**, and *A.penumbrella***sp. nov.** Two taxa are newly combined: *Athrypsiastishalmaherella* (Lvovsky, 2014), **comb. nov.** and *Paralectarosiflora* (Meyrick, 1930), **comb. nov.**

## ﻿Introduction

Xyloryctidae is a large family of palaeotropical Gelechioidea containing approximately 542 accepted described species (Bánki et al. 2024). Many of these occur in Australia and a large number are undescribed. Knowledge of their diversity in Southeast Asia to New Guinea is poor. This revision is part of a wider examination by the authors of the substantial holdings at the NHMUK of a group of — principally white — xyloryctid moths from the Oriental region and New Guinea. Due to the size of this project (these holdings comprise, in aggregate, almost 1000 specimens and at least 70 undescribed species) we have divided our study into two parts. In this first part of the study, we examine specimens that we have filtered from this large group of material on the basis that R3 is present and M3 and CuA1 are stalked in the forewing. This group has never previously been studied in any detail.

Edward Meyrick described a genus of xyloryctid moth, *Athrypsiastis* Meyrick, 1910 (type species *Athrypsiastisphaeoleuca* Meyrick, 1910), containing species of principally white xyloryctid moths from these regions which exhibit both the above characters in the forewing venation (the three other members of the genus being *Athrypsiastissymmetra* Meyrick, 1915, *A.rosiflora* Meyrick, 1930, and *A.salva* Meyrick, 1932). John F. Gates Clarke (Clarke 1955) refers to these species without treating them in detail. Although there are a few references to *Athrypsiastias* in pest literature which we cite below, the only taxonomic literature since Meyrick and Gates Clarke on *Athrypsiastis* is the description of two species, *Athrypsiastiaschionodes* Diakonoff, 1954 and *A.delicata* Diakonoff, 1954, from single specimens, both from New Guinea (Diakonoff 1954). Meyrick refers to these forewing venation characters in his description of *Athrypsiastis*. These characters differ from the forewing venation characters described by Meyrick for *Metathrinca* Meyrick, 1908 and *Linoclostis* Meyrick, 1908 in that, in those two genera, R3 (vein 9 in Meyrick’s notation) is absent from the forewing.

Apart from *Athrypsiastis*, the only other genus containing a white xyloryctid moth from these regions with our selected forewing venation criteria is *Topiris* Walker, 1863. The only described species in this genus is *Topiriscandidella* Walker, 1863. The type of this species is a holotype by monotypy.

The principal aim of this part of the study was to examine the diversity and phylogeny of the species considered in our broader study with the two forewing characters referred to above. All these species, and the described species of *Athrypsiastis*, display the morphological characters for Xyloryctidae described in Common (1990: 227). Family placement is therefore not controversial. Generic placement, however, involved clarifying the relationships of *Topiris* with respect to *Athrypsiastis* and its type species (*A.phaeoleuca*), and whether the taxa we examined were referable to either genus or whether a new genus or genera needed to be established.

Morphological study of the type of *T.candidella* has long been problematic. The holotype must have been more or less intact when it was described, as Walker refers to characters in the head and the abdomen as well as to its hindwings (Walker 1863: 521–522) but prior to 1929 it had become badly damaged. Fletcher (1929: 227) wrote of this specimen as follows: “Unidentifiable: Meyrick notes (in litt. May 1927) that he has twice examined the type, which may be an *Athrypsiastis*, but has been mended by having the hindwings of a *Hyponomeuta* [sic!] attached; better neglected”.

All that remains of the specimen today are the pin, part of the thorax, two middle legs, the coxa of one of the hindlegs and the forewings (Fig. 4). The hindwings referred to by Fletcher have been detached at some stage from the forewings (Fig. 87) and have now been separately labelled and mounted.

We nevertheless noted early on while embarking on our study that many of the previously unexamined specimens with the above venational characteristics bore a striking resemblance also in size, shape, and complete absence of markings on white forewings, to the holotype of *T.candidella*. There is also a single specimen among these holdings in the NHMUK accessions, presented by the Commonwealth Institute of Entomology in 1948, which has attached to it a label stating “*Topiriscandidella*, Walk. E. Meyrick det.”.

When we started this project in 2017, there was no realistic prospect of standard DNA sequencing techniques using the usual DNA barcode primers for Lepidoptera producing any data. However, in November 2022 we submitted a tissue sample of this type (which was probably collected between December 1855 and January 1856: see Remarks on *T.candidella*) to a pilot study at the NHMUK to test a recently adopted genome skimming technique (see e.g., Trevisan et al. 2019), which provides a low level fragmentary coverage across the genome, biased towards mitochondrial data especially in old and museum specimens. The results of the analysis of this tissue sample had a transformative effect on the output from this study, finally enabling us to evaluate Meyrick’s suggestion for *Topiris* that the only exemplar was “better neglected”.

We also sought to clarify the provenance of the holotype of *T.candidella.* Based on its white circular label with the three handwritten letters ‘SAR’, this specimen appeared to have been collected by Alfred Russel Wallace during his stay in Sarawak (Malaysian Borneo) and labelled by his assistant Charles Allen (Cranbrook and Mann 2016). It is an additional species of insect described from Alfred Russel Wallace’s Sarawak collections to those listed in Polaszek and Cranbrook (2006), who, however, did not focus on microlepidoptera. The moths in the NHMUK corresponding to the light collection of Wallace which he tabulated (Wallace 1869: 96) have mostly not been catalogued.

We show that *Topiris* is a genus which is widely distributed throughout the Oriental Region from Sulawesi to northern Thailand, and which extends northwards as far as Hangzhou, China. *Athrypsiastis* occurs from Sulawesi to New Guinea. We also show that both *Athrypsiastis* and *Topiris* fall within a clade of core Xyloryctidae which includes *Xyloryctaluteotactella* (Walker, 1864).

## ﻿Materials and methods

The abbreviations used throughout for depositories are as follows:

**NHMUK**Natural History Museum (formerly British Museum of Natural History), London, UK;

**NBC**Naturalis Biodiversity Centre, Leiden, Netherlands.

The specimens examined for this paper, including all type material, are held at the NHMUK unless otherwise expressly stated. All types are adults which were deposited in the main NHMUK collection on or prior to 24 January 2024.

External examination of materials was carried out using a Nikon SMZ800 microscope. The illustrated material (other than Figs 74–76, 83) was photographed using a Canon EOS 5DSR camera and MP-E 65 mm lens equipped with a Stackshot system operated by Helicon Remote software (version 3.8.4 W); the images were combined with Helicon Focus software (v. 6.7.1). Figs 74–76, 83 were photographed using a Zeiss Axioskop microscope with a Canon EOS 700D camera attached. The images were stacked with Helicon Focus software (v. 6.7.1).

Genitalia dissection and mounting followed Robinson (1976). All genitalia preparations were also made using the same Nikon SMZ800 microscope. Descriptions of the genitalia follow Klots (1970) and Kristensen (2003).

Descriptions of wing venation follow the Comstock-Needham method as summarised in Miller 1970 with the modifications adopted in Common (1994: 14). Venation preparations were made as follows:

1. The right-hand pair of wings were dampened with a single drop of 100% ethanol or isopropyl alcohol and removed from the thorax using fine forceps;
2. The wings were transferred to 5% sodium hypochlorite solution for a maximum of five minutes;
3. The wings were then transferred to water (all transfers made with a fine paintbrush) and immediately thereafter to a staining block containing 1% aqueous eosin and macerated overnight;
4. The wings were then transferred to staining blocks containing, respectively, water, 50% alcohol, 100% alcohol and euparal essence in that order. Any scales which remained following the maceration process were carefully removed with a mounted pin feather of a Woodcock (*Scolopaxrusticola* Linnaeus, 1758) when the wings were in 100% alcohol;
5. Finally, the wings were transferred to a slide dampened with Euparal essence and then mounted in Euparal.

Specimens sent to BOLD for Sanger sequencing with failure tracking (see below) were sorted by locality and physical appearance and pre-1960 specimens excluded. The selected specimens were sampled by removing one or two legs, where possible from the left-hand side of a specimen, and placing them in an individual well containing three drops of absolute ethanol to minimise electrostatic movement. Care was taken to wipe forceps with tissue paper between each handling to minimise risk of cross contamination. Plates were then sent to BOLD for DNA sequencing following standardised procedures described in Ratnasingham and Hebert (2007) to try to assemble bidirectionally a full length 658 bp DNA barcode of COI-5P using the Folmer-modified primers LepF1 and LepR1. In many cases, because of the age of the specimens, this was done using primer pairs amplifying around or just more than half length (overlapping fragments approximately 307–407 bp using primer pairs MlepF1/LepR1 and LepF1/MlepR2). We refer here to this procedure as ‘failure tracking’. In cases where only one of these fragments was sequenced, they were not enough to generate a Barcode Index Number (BIN) but more than enough to identify the species to a known sequence (Hajibabaei et al. 2006; Meusnier et al. 2008).

A small number of specimens collected in the 1930s were also sent to BOLD for next generation sequencing (Shokralla et al. 2014).

Variable length sequences obtained from BOLD were aligned using MAFFT online (https://mafft.cbrc.jp/alignment/server) and checked by eye in Bioedit v. 7.2.5 (Hall 1999).

### ﻿Genome skimming

We used the same protocol to recover old, degraded DNA from the Walker type specimen (NHMUK010219690) as reported in Sterling et al. (2023). However, the bioinformatic approach used here differs from that which succeeded for two 1880’s specimens, as the assembly here proved more challenging. We therefore explored another approach that has proved useful for old museum specimens (see e.g., Bernstein and Ruane 2022), namely the program MITObim v. 1.9.1 (Hahn et al. 2013).

Raw sequence data were first processed with fastp v. 0.23.4 (Chen et al. 2018; Chen 2023), with default quality filtering enabled, trimming adapters, and discarding reads below 15 bp or above 46 bp in length. These longer reads were discarded due to several highly over-represented adapter-like sequences more than 46 bp. The mitochondrial genome was then assembled using the --quick option of MITObim v. 1.9.1 (Hahn et al. 2013), using a previously published Xyloryctidae mitochondrial genome (Su et al. 2020; GenBank: MT547768) as a reference, with kmer baiting set to 21, and maximum iterations set to 30. The resulting mitochondrial assembly was annotated using MITOS2 (Bernt et al. 2013; Arab et al. 2017; Donath et al. 2019) on the Galaxy cluster (https://usegalaxy.eu/; The Galaxy Community 2022). It is important to note here, considering that genome skims were sensitive to possible contaminants, that no other *Topiris* species related to the tissue fragment of NHMUK010219690 were sequenced alongside that sample in the NHMUK laboratory; all the DNA barcoding of modern specimens was done separately at Guelph, Canada (BOLD). COI identified by MITOS2 from the mitogenome assembly was then aligned with modern sequences (MT547768 and the modern DNA barcodes obtained from *Topiris* species) and checked for any differences using Bioedit v. 7.2.5.

### ﻿Pairwise distances

Pairwise distances were calculated using Bioedit v. 7.2.5 for comparable nucleotides as described in Sterling et al. (2023).

### ﻿Phylogenetics

During ‘Neighbor Joining’ searches on BOLD using the tree view and image building options, we discovered another sequence belonging to *Topiris*: BIN, BOLD:AAL9269 (*Topiris* sp. specimen/slide number ID RMNH.INS.20000, Field ID EvN2005195, Accession no. HM877537). We did not physically examine this specimen, but we include an image and a short note in the morphological systematics section. This approach also enabled us to discover a complete mitochondrial genome (MT547768) for an exemplar from China labelled as ‘*Linoclostisgonatias*’ but representing *Topirissalva* (see Discussion).

The morphological matrix was generated in and transposed using MS Excel (Suppl. material 1) and formatted for MrBayes input as a nexus file, in which both missing and inapplicable data were treated as missing (question marks). For the purpose of coding character 49, the right hand valva (ventral view) was measured from base to apex (except for *A.edelweissella* for which the left valva was measured) and the aedeagus measured in sections, where necessary, to take account of curvature.

The morphological and molecular COI-5P data were formatted as Nexus files with the following settings for independent and combined analyses (independent matrices were also run using the model specified with the lset command applicable to that dataset). They were run with command line execution using MrBayes v. 3.2.7 for Windows 64 using two partitions (morphology under gamma rate model, COI with invgamma rate model, nst = 6, combined under two partitions with these respective models), state frequencies, shape, revmat and pinvar variables all unlinked, 10 million generations with a burn-in of 25%. The resulting consensus tree file was plotted in FigTree v. 1.4.3, to show posterior support values with higher than 0.940 posterior credibility. The tree was output as Newick format and imported in TNT format into WinClada (Nixon 2002). From MrBayes output, a tree was generated in FigTree v. 1.4.3 (http://tree.bio.ed.ac.uk). and then branches were transformed to a cladogram and the tree exported in Newick format and then simplified in a text editor in order to remove branch lengths and taxon names were edited down to the taxon numbers that had been tagged to the end of each taxon label. The morphological matrix in the order of these taxon numbers was opened in WinClada. The — now greatly simplified — Newick tree was also opened (after duplicating it in that file) in WinClada and exported as .emf.

In Phyml 3.0 online (Guindon et al. 2010; http://www.atgc-montpellier.fr/phyml) and in IQTree online (http://iqtree.cibiv.univie.ac.at/) the COI-5P only matrix was run under an ML model. Questionmarks were scored as N for missing data, and the matrix was run under a GTR + Gamma model (as automatically implemented under the Akaike Information Criterion), i.e., with defaults settings except for ABayes supports. In IQTree the matrix was run under default settings with Ultrafast bootstrap (1000 iterations).

For the cladistic combined matrix, which, unlike the DNA only tree, is expressly chimaeric, sometimes including different sexes, a male exemplar of each taxon, where available, was selected for scoring external and genitalia characters and states in MS Excel (dataset in Suppl. material 1). All characters and their states were checked using the character diagnoser in WinClada v. 1.00.08. COI data, where available, for the same male exemplar was also used. Female exemplars, where available, were selected as follows. The female of *T.salva* (Sample ID MSterling045, Museum ID NHMUK010219702) shared the same haplotype as the respective male (NHMUK010922992) so only the last sequence was used (representing BINBOLD:ADS0105). For *Topiris* sp. (BOLD:ADR9781) (NHMUK010922995, NHMUK010923170, NHMUK010923171) both haplotypes were used in at least one tree. Both haplotypes of *T.schneeweissella* were used (the female, NHMUK010923174, is 0.6%, i.e., three nucleotides, pairwise divergent from the male (NHMUK010922999) that was used for morphological scoring and in the combined analysis. The female of *Metathrincaargentea*Wang et al., 2000 (BOLD:ADR9782; Process ID METAT047-18; Sample ID MSterling047, Museum ID NHMUK010923245) was 0.3% (i.e., two nucleotides) divergent from the male exemplar used (MSterling046, NHMUK010219680) for COI and scoring morphological characters. The female exemplar of *T.albidella* is topotypical with the male exemplar (both were collected in October 1985) but all five sequence fragments represented different haplotypes. The COI data used here for the outgroup of *Xyloryctaluteotactella* is a sequence on BOLD (Process ID ANICV389-11) corresponding to BINBOLD:AAF7533, representing a specimen (ANIC Database No. 31 047649 from Sydney (Cabramatta, -33.895, 150.936, KF400939) reared from fruit of *Hakeasericea* Schrad. & J.C. Wendl. (Proteaceae). The male and female exemplars of *X.luteotactella* used for scoring morphological characters are both from Sydney, New South Wales (NHMUK014584797 and NHMUK014171179 respectively). The type material of *X.luteotactella* is from Sydney. The male and female exemplars of *Metathrincatsugensis* (Kearfott, 1910) used for morphological scoring are both from Japan but we used the sequence on GenBank from South Korea (HM377859) for the molecular/combined trees (BINBOLD:AAK7146; on the basis it did not differ from the 407 bp we sequenced (Process ID METAT054-18) from a Japanese specimen, NHMUK010923020). *M.tsugensis* is the only species of *Metathrinca* Meyrick, 1908 known in Japan and Korea. The male of *Linoclostisgonatias* Meyrick, 1908 is unknown and we have no morphological data on the exemplar used by Su et al. 2020 for extraction of the mitochondrial genome (MT547768) described in their paper although, for the reasons set out in the Discussion section, we show that this exemplar does not represent *L.gonatias*.

In order to provide a phylogenetic scaffold in which we could check the placement of *Topiris*, we aligned one mitochondrial gene (COI) and four-seven nuclear genes for six taxa, namely *Martyringa* sp. NKU-WQY0093 (BIN not available), ‘*Xylorycta*’ sp. MM-2011 (‘BOLD:AAD7429’), *Eumenodoraencrypta* Meyrick, 1906b (‘BOLD:AAM4364’), *Izathaaustera* (Meyrick, 1883), *I.peroneanella* (Walker, 1864), *Tymbophorapeltastis* Meyrick, 1890 (‘BOLD:AAH7726’), and *Metathrincatsugensis* (these genes were EF-1a in two segments; Wingless; RpS5; CAD; MDH; GAPDH; IDH) downloaded from GenBank from the studies of Kaila et al. (2011) and Wang and Li (2020). 1500 bp of COI were used from the mitogenomic assembly from MITObim for the holotype of *Topiriscandidella*. These data were aligned separately using MAFFT (online) and then concatenated against exemplar numbers in MS Excel. We added unique haplotypes from COI-5P from our own DNA barcoding for two species of *Athrypsiastis*, eight species of *Topiris*, and the whole of COI for ‘*Linoclostisgonatias*’ (MT547768) but omitted sequences below 200 bp and near-identical ones for this analysis. The total alignment represented 6405 nucleotides including gaps between genes to aid visual checks in protein format using Bioedit v. 7.2.5 (Hall 1999). We then ran a tree in a maximum likelihood setting using Phyml 3.0 online (Guindon et al. 2010; http://www.atgc-montpellier.fr/phyml) and ran it using automatic model test (Akaike Information Criterion; resulting under model GTR+G) with SPR pruning-rerooting and with support displayed according to the ABayes algorithm. This tree was rooted in FigTree on Martyringacf.xeraula.

The trimmed genome skim data and mitogenome assembly for NHMUK010219690 have been deposited in the European Nucleotide Archive (https://www.ebi.ac.uk/ena/browser/view), Sample Accession: ERS22934821; Mitogenome Accession: ERZ25037365). The DNA barcodes and sequence fragments of *Topiris* and *Athrypsiastis* and two *Metathrinca* are available in BOLD dataset DS-TOPIRIS (http://v4.boldsystems.org/index.php/MAS_Management_DataConsole?codes=DS-TOPIRIS). The DNA barcode of *Topiriscandidella* (accession number PP131475) and the whole of COI (accession number PQ676473) assembled from the genome skim are also provided in the last dataset. A table of all taxa and accession numbers used is included as Suppl. material 2.

## ﻿Results

### ﻿DNA barcoding

Successful COI sequences were obtained from a total of 21 exemplars within our selected materials. Sequences of *Metathrincatsugensis* and *Metathrincaargentea* (two species within *Metathrinca* for which full DNA barcodes are available) were included in the analysis and a sequence of *Xyloryctaluteotactella* (KF400939) was used as the outgroup. Fifteen of these sequences were obtained by Sanger sequencing with ‘failure tracking’ at BOLD, from specimens collected in or after 1985, 12 full DNA barcodes of 658 bp being obtained, while the remaining three sequences represented partial fragments of 307–368 bp (BOLD project METAT). Four short sequences were obtained by a mixture of failure tracking and next generation sequencing (BOLD dataset DS-DEPAL) from specimens collected in the mid to late 1930s, of 133–325 bp.

### ﻿Genome skimming

**Figure 1.**
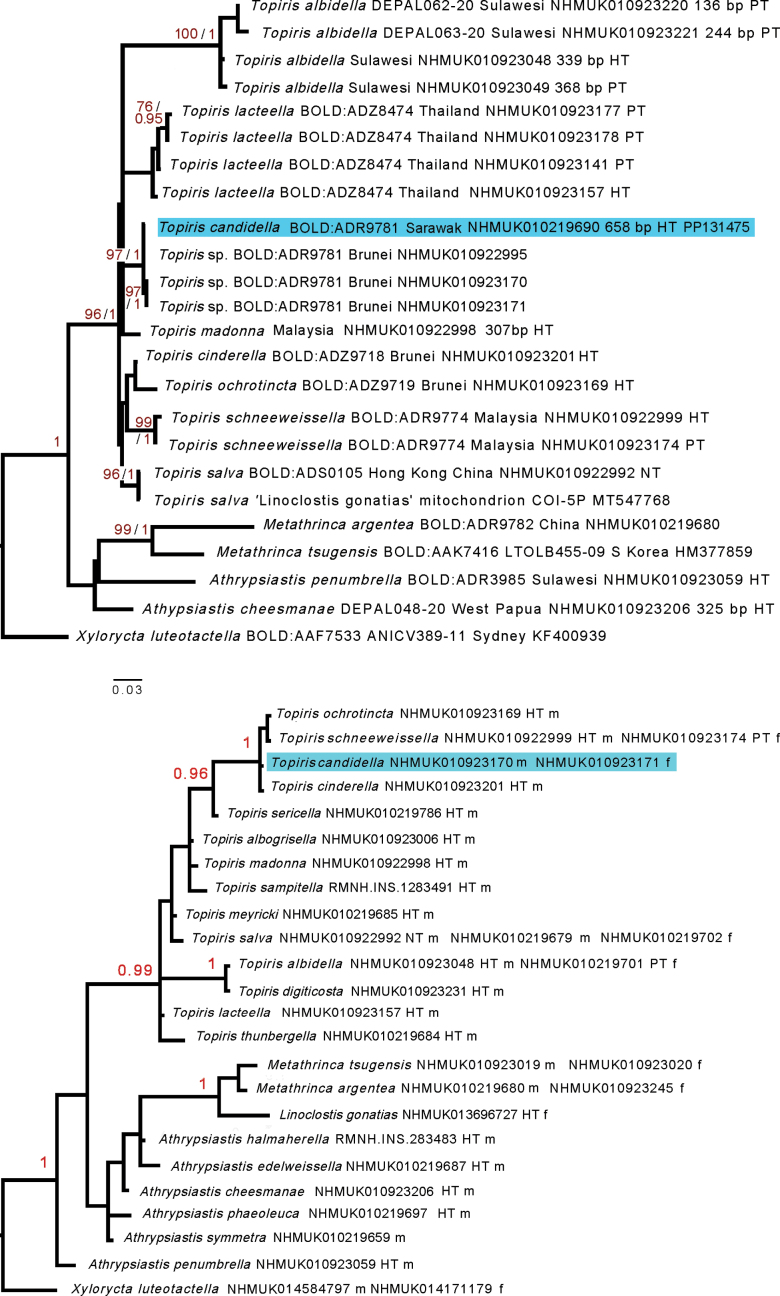
**a** analysis using IQTree (UFbootstrap values as percentage of 1000) and MrBayes 3.2.7 (/posterior probabilities) of DNA barcodes of *Topiris*, including all unique haplotypes sequenced (except one 133 bp sequence of *Topirisalbidella*), showing their relationship to the genus *Athrypsiastis* and two exemplars of *Metathrinca*, rooted by *Xylorycta* (type species: *X.luteotactella*). The reconstructed DNA barcode *Topiriscandidella* (NHMUK010219690, highlighted in blue) from Sarawak falls in a clade with specimens of other members of the BIN cluster BOLD: ADR9781 from which it differs by 1-3 bp. *Topiris* is recovered as monophyletic. HT = holotype, PT = paratype, NT = neotype; bp = no of nucleotides sequenced, if not 658 **b** morphological dataset (65 characters) as analysed for 24 taxa in MrBayes 3.2.7 under a gamma model, rooted by the type species of *Xylorycta* (*X.luteotactella*). The specimen numbers and sexes of the exemplars used for describing the morphological characters are set out in this figure. Where possible, the holotype (HT) [or neotype (NT) was used for scoring. PT = paratype, NT = neotype, m = male, f = female. The male of *Topiriscandidella* (highlighted in blue) is represented by a modern specimen (NHMUK019223170), since the holotype is incomplete. *T.candidella* is morphologically cryptic with *T.cinderella* except for small differences in the male genitalia.

A total of 63.9 million paired-end reads were sequenced for Walker’s type specimen (NHMUK010219690). Trimming and quality filtering in fastp resulted in 25.8 million paired-end reads after removal of all fragments below 15bp or more than 46bp. MITObim successfully reconstructed a mitochondrial genome in a single contig of 15,651 bp, with an average coverage of 112×, from a total of 51,230 reads (mean coverage across the DNA barcode region was 62.4× (range: 6–112), assembled from 1,384 sequence reads; mean coverage across the whole of COI was 56.1× (range: 0–364). The 658 bp DNA barcode sequence assembled from the type specimen (Process ID METAT287-24) differs by 1–3 bp from the two modern haplotypes (Process IDs METAT027-24; METAT211-19) found in Brunei (Fig. 1a).

### ﻿COI data

Fig. 1a, using DNA barcode data only, recovers both *Metathrinca* exemplars (*M.tsugensis* and *M.argentea*) (UFboot = 99; pp = 1) and *Topiris* (UF boot = 96; pp = 1) as clades, whereas *Athrypsiastis* remains paraphyletic. All species with multiple exemplars are fully supported, with the exception of *T.lacteella*, which in some trees was paraphyletic with respect to *T.albidella*. These clades include *Topirissalva* (UF boot = 96; pp = 1); the COI-5P of the exemplar identified as the ‘*Linoclostisgonatias*’ mitochondrion (MT547768) is divergent by two nucleotides (0.3%) from NHMUK010922992, representing BINBOLD:ADS0105 (the specimen we here designate as the neotype of *Topirissalva* (Meyrick, 1932) (NT in Fig. 1a), as well as our other sequences of *T.salva* (not shown because they are identical haplotypes). The key results here are that *Topiris* (UF boot = 96; pp = 1) is monophyletic and that the *Topiriscandidella* holotype from Sarawak (highlighted in blue) and modern exemplars of BINBOLD:ADR9781 from Brunei also come together (UFboot = 97; pp = 1). Both sexes of *T.schneeweisella* are also supported as conspecific (UFboot = 99; pp = 1).

### ﻿Morphological data

Fig. 1b shows the output from running (in MrBayes 3.2.7) the data from the 65 morphological characters which are contained in the cladistic combined matrix (Suppl. material 1) separately from the COI data. This analysis contains data from a further 11 taxa for which morphological data, but no COI, is available, including the holotype of *Linoclostisgonatias* Meyrick, 1908, which is included for the purposes of testing whether it forms a clade with *Topirissalva*.

*Topiris* is recovered as a supported clade (pp = 0.99) of 14 taxa. Within *Topiris* there are three supported sub-clades: *T.cinderella* + *T.candidella* + *T.schneeweissella* + *T.ochrotincta* (pp = 1); this sub-clade + *T.sericella* (pp = 0.96) and *T.digiticosta* + *T.albidella* (pp =1). *Metathrinca* and the true *Linoclostisgonatias* form a clade (pp = 1); notably, *L.gonatias* does not form a clade with *Topirissalva*.

### ﻿Combined Data

**Figure 2.**
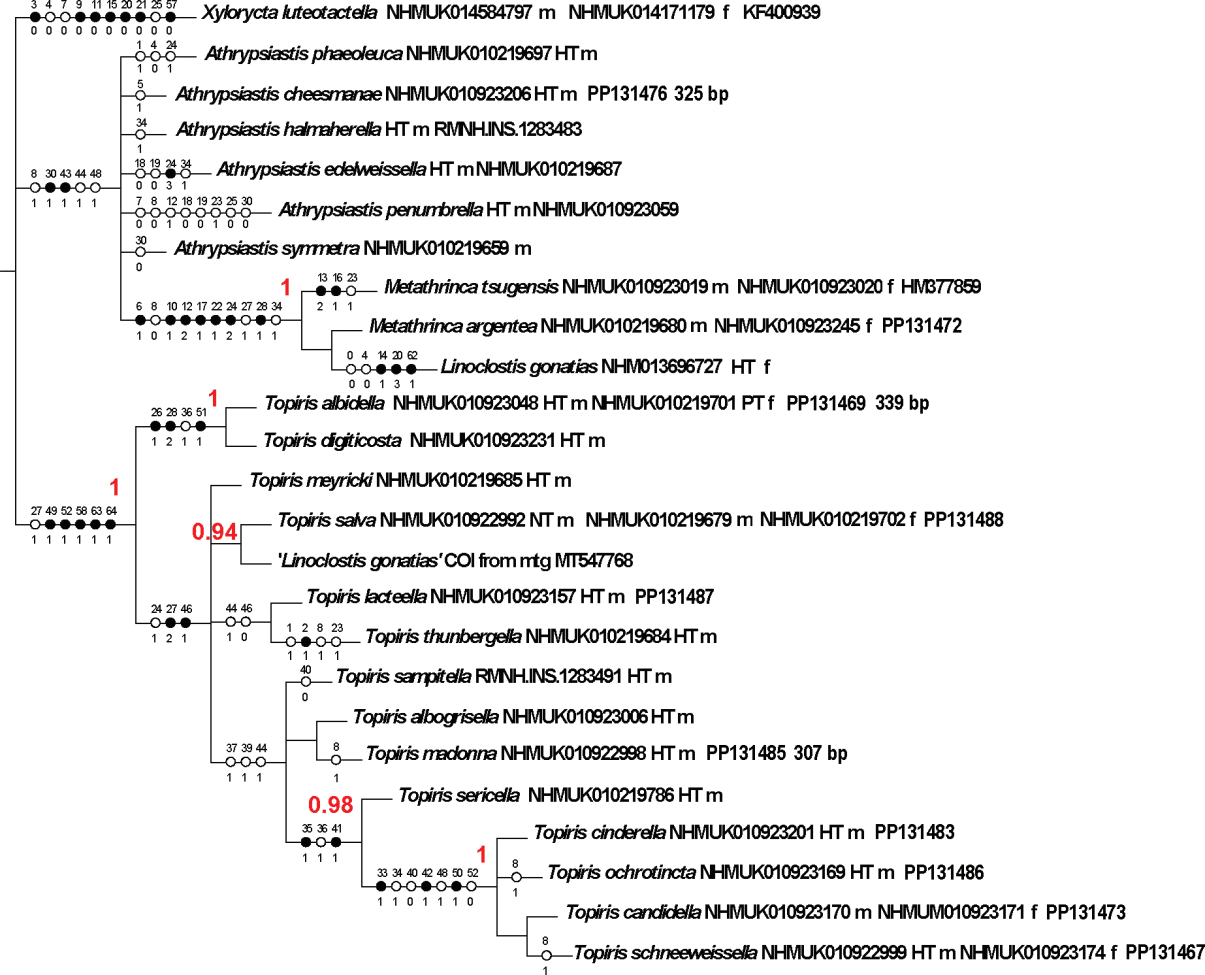
Combined morphological and molecular tree run in MrBayes 3.2.7 under a split-partition gamma and inverse gamma model (with posterior supports where > 0.94) and with the morphological part of the matrix mapped onto this tree using WinClada v. 1.00.08. This tree shows supporting characters (filled circles show forward nonhomoplasious changes, which may be either synapomorphies or autapomorphies; unfilled circles show forward changes with homoplasy or reversal) for *Topiris* and other ingroup species. The support values in red are posterior probabilities of at least 0.94 for a two partition run in MrBayes 3.2.7 for the combined 723 character matrix. HT = holotype, NT = neotype, PT = paratype, m = male, f = female. Character numbers appear above the circles. State numbers appear below the circles.

Fig. 2 shows the topology resulting from running the combined DNA barcode and morphological data in MrBayes 3.2.7, and plotting the posterior probabilities from the consensus tree of 14476 trees sampled for supported nodes, with morphological character mapping in Winclada.

The *Topiris* clade of 14 taxa is supported (pp = 1). Also supported are the three sub-clades of *T.cinderella* + *T.ochrotincta* + *T.schneeweissella* + *T.candidella* (= *Topiris* sp.) (pp = 1), this sub-clade + *T.sericella* (pp = 0.98); and *T.digiticosta* + *T.albidella* (pp = 1). *Metathrincatsugensis* + *M.argentea* + *Linoclostisgonatias* form a clade (pp = 1), albeit with a long branch (only female morphological data is available for the holotype of *L.gonatias*). *Athrypsiastis* + the *Metathrinca*/*Linoclostis* clade form a polytomy with the outgroup. Named species of *Athrypsiastis* are paraphyletic with respect to the type species, *A.phaeoleuca.* There is only submarginal support (pp = 0.94) for a clade of *Topirissalva* and COI-5P from the so called ‘*Linoclostisgonatias*’ mitochondrion (MT547768), but this is explained by full morphological data being available for the former whereas no morphological data is available for the latter.

The informative characters for the genus *Topiris* and its supported sub-clades are all in the genitalia. Filled circles show forward nonhomoplasious changes, whereas unfilled circles show forward changes with homoplasy or reversal. By default, Winclada only maps characters that can be unambiguously optimised for their placement on the tree and 13 of the 65 morphological characters were thereby excluded from being plotted. The informative characters and states for genus *Topiris* are: aedeagus sheath with recurved filament-like distal projection (Fig. 2, character 49 state 1; and Figs 68, 69); aedeagus sheath with small distal thickening (Fig. 2, character 52 state 1; and Fig. 68), this state being reversed in the *T.candidella* sub-clade; sclerotised part of antrum long and narrow (Fig. 2, character 58 state 1; and Fig. 72) and corpus bursae large, elongate and without signum (Fig. 2, character 63 state 1 and character 64 state 1; and Fig. 73). It should however be noted that, in relation to these female characters, the female genitalia of *Athrypsiastis* are unknown.

Fig. 2 also shows informative characters in the male genitalia for three supported sub-clades. In the *T.albidella* sub-clade these are: gnathos not fused medially, gnathos with two narrow lateral posterior projections (Fig. 2, character 26 state 1 and character 28 state 2; Figs 47, 74) and aedeagus with small sclerite medially (Fig. 2, character 51 state 1; Fig. 70). In the *T.candidella* sub-clade these are: vinculum diverging strongly distad of saccus (Fig. 2, character 33 state 1; Fig. 77), sacculus with strong mesad shoulder (Fig. 2, character 42 state 1; Fig. 79) and distal projection of aedeagus short (Fig. 2, character 50 state 1; Fig. 69). For *T.candidella* sub-clade + *T.sericella* these are: valva with sclerotised setose process at base of ventral membrane from the costa (Fig. 2, character 35 state 1; Fig. 78) and distal half of valva tapered (Fig. 2, character 41 state 1; Fig. 80).

The *Metathrinca* + *Linoclostis* clade is distinguished from both *Topiris* and *Athrypsiastis* by informative characters in the head: labial palps short (less than 2× diameter of eye) (Fig. 2, character 6 state 1); wing venation: R3 absent and R4 pre-apical in forewing (Fig. 2, character 10 state 1 and character 12 state 2); wing pattern: forewing with subterminal line and with iridescent silver patches between subterminal line and termen (Fig. 2, character 17 state 1 and character 22 state 1); and male genitalia: uncus posteriorly bifid and laterally rounded and gnathos with two broad lateral posterior projections (Fig. 2, character 24 state 1 and character 28 state 1).

### ﻿Framework tree including nuclear genes as relating to previous studies of Xyloryctidae phylogeny

**Figure 3.**
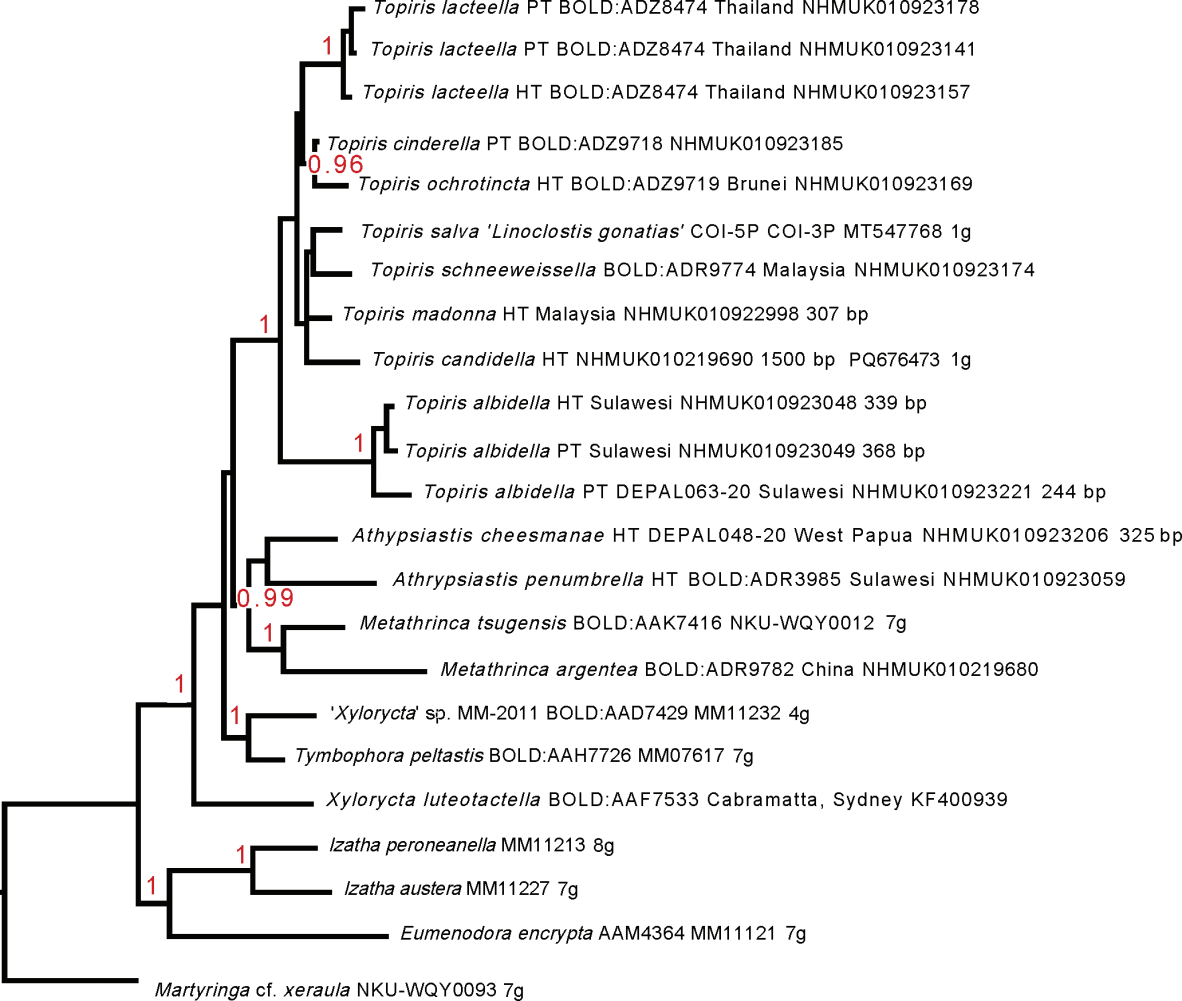
‘Framework’ tree showing position of *Topiris*, *Metathrinca* and *Athrypsiastis* falling within ‘core Xyloryctidae’ (i.e. with the clade including the type species of *Xylorycta*, *X.luteotactella*), rather than with the genera *Eumenodora* plus *Izatha* (part of Xyloryctidae*sensu lato*, rooted by *Martyringa* (Lecithoceridae)). The combined matrix with the COI data was analysed in the context of multi-gene data (for six terminals that include labels ‘MM’ and ‘NKU’; all of COI plus between four-seven nuclear genes) using Phyml 3.0 (nodes are shown with ABayes support values in red). The total number of genes is shown (‘g’) for taxa with multiple loci, where not only COI-5P. COI sequences below 200 bp or near-identical are excluded. Support values shown are ABayes values > 0.94 from analysis in Phyml 3.0 (online) under a GTR+G model.

The topology of Xyloryctidae with respect to the previous studies of Kaila et al. (2011), Heikkilä et al. (2014) and Wang and Li (2020) is analysed in Fig. 3. This multigene analysis suggests the presence of two xyloryctid clades (pp = 1; note that these two clades share at least four genes; see Fig. 3, Suppl. material 2) within Xyloryctidae, one including the New Zealand genus *Izatha* and the Australian taxon *Eumenodoraencrypta*, and the other including ‘core xyloryctids’ (*Xyloryctaluteotactella*, ‘*Xylorycta*’ sp. + *Tymbophorapeltastis* (pp = 1). *Metathrincatsugensis* (used in the prior studies) + *M.argentea* fall together (pp = 1), and *Metathrinca* + *Athrypsiastis*is also supported (pp = 0.99). *Topiris* is monophyletic (pp = 1). The analysis lends support (pp = 1) to *Topiris* belonging within the ‘core xyloryctids’ rather than a clade within Xyloryctidae (*sensu lato*) which includes *Eumenodora* (Australian) and *Izatha* (New Zealand).

### ﻿Checklist of treated species

#### ﻿*Topiris* Walker, 1863

*candidella* group

*Topiriscandidella* Walker, 1863 (type species).

*Topiriscinderella* Sterling & Lees, sp. nov.

*Topirisochrotincta* Sterling & Lees, sp. nov.

*Topirisschneeweissella* Sterling & Lees, sp. nov.

*Topirissericella* Sterling & Lees, sp. nov.

*Topiris* ‘RMNH.INS.20000’ [BIN, BOLD:AAL9269].

*salva* group

*Topirissalva* (Meyrick, 1932; *Athrypsiastis*), comb. nov.

*Topirisalbogrisella* Sterling & Lees, sp. nov.

*Topirislacteella* Sterling & Lees, sp. nov.

*Topirismadonna* Sterling & Lees, sp. nov.

*Topirismeyricki* Sterling & Lees, sp. nov.

*Topirissampitella* (Lvovsky, 2014; *Metathrinca*), comb. nov.

*Topiristhunbergella* Sterling & Lees, sp. nov.

*albidella* group

*Topirisalbidella* Sterling & Lees, sp. nov.

*Topirisdigiticosta* Sterling & Lees, sp. nov.

#### ﻿*Athrypsiastis* Meyrick, 1910

*Athrypsiastisphaeoleuca* Meyrick, 1910 (type species).

*Athrypsiastischeesmanae* Sterling & Lees, sp. nov.

*Athrypsiastischionodes* Diakonoff, 1954

*Athrypsiastisdelicata* Diakonoff, 1954

*Athrypsiastisedelweissella* Sterling & Lees, sp. nov.

*Athrypsiastishalmaherella* (Lvovsky, 2014; *Metathrinca*), comb. nov.

*Athrypsiastispenumbrella* Sterling & Lees, sp. nov.

*Athrypsiastissymmetra* Meyrick, 1915.

#### ﻿*Paralecta* Turner, 1898 (type species is *Xyloryctatinctoria* Lucas, 1894)

*Paralectarosiflora* (Meyrick, 1930; *Athrypsiastis*), comb. nov.

### ﻿Morphological systematics

**Keys**

#### ﻿Key to differentiate *Athrypsiastis*, *Topiris*, and *Metathrinca/ Linoclostis*

Note: The relationship between *Metathrinca* and *Linoclostis* is complex, as both genera require substantial revision. This will be the subject of a separate paper. However, in *Linoclostis*, M3 and CuA1 are stalked in the forewing whereas, at least in the type species of *Metathrinca* (*Ptochoryctisancistrias* (Meyrick, 1906a)), M3 and CuA1 are separate in the forewing.

**Table:** 

1	Forewing without iridescent silver patches between subterminal line and termen	**2**
–	Forewing with iridescent silver patches between subterminal line and termen	***Metathrinca/Linoclostis***
2	Medial plate of gnathos strongly sclerotised and strongly projecting posteriorly from the lateral arms (Fig. 76)	***Athrypsiastis***
–	Medial plate of gnathos lightly sclerotised and weakly projecting posteriorly from lateral arms, or absent (Figs 75, 74)	***Topiris***

#### ﻿Key to species of *Topiris* based on external features

Note: Some members of the *candidella* and *salva* groups cannot be distinguished from each other based on external features and require examination of the genitalia for species level determination. This key based on external features distinguishes the species insofar as this is possible.

**Table:** 

1	Forewings with dark brown terminal line	**2**
–	Forewings without dark brown terminal line	**3**
2	Terminal line continuing until after tornus	***T.albidella***
–	Terminal line terminating prior to tornus	***T.digiticosta***
3	Forewing ground colour ochreous	**4**
–	Forewing ground colour not ochreous	**5**
4	Forewing ground colour pale ochreous	***T.ochrotincta***
–	Forewing ground colour golden ochreous	***T.thunbergella***
5	Flagellum of male antenna covered with white metallic scales on dorsal surface and laterally for most of length of pectinations	***T.madonna***
–	Flagellum of male antenna otherwise	**6**
6	Hindwing with indistinct greyish terminal line	***T.sericella***
–	Hindwing without indistinct greyish terminal line	**7**
7	Forewing with narrow, well-defined brownish-grey dorsal patch	***T.albogrisella***
–	Forewing with dorsal patch less well-defined or no dorsal patch	**8**
8	Forewing dull white without dorsal patch	***T.meyricki***
–	Forewing otherwise	***T.lacteella* , *T.salva* , *T.candidella* , *T.schneeweissella* , *T.cinderella* , or *T.sampitella***

#### ﻿Key to species of *Topiris* based on male genitalia

Note: *Topirissampitella* is omitted from male genitalia key due to lack of data.

**Table:** 

1	Long sclerotised process arising from base of ventral membrane from costa of valva (Fig. 78)	**2**
–	Base of ventral membrane from costa of valva without long sclerotised process	**6**
2	Long sclerotised process setose throughout	**3**
–	Long sclerotised process setose only on apex (Fig. 37A)	***T.sericella***
3	Postmedial part of valva narrow	**4**
–	Postmedial part of valva comparatively broad	**5**
4	Ventral membrane from costa of valva distally raised (Fig. 81)	***T* . *cinderella***
–	Ventral membrane from costa of valva distally flat (Fig. 82)	***T.candidella***
5	Distal hook of saccular process long and narrow (Fig. 36A)	***T.schneeweissella***
–	Distal hook of saccular process shorter and broad (Fig. 35A)	***T* . *ochrotincta***
6	Gnathos not fused medially	**7**
–	Gnathos fused medially	**8**
7	Distal margin of valva strongly concave with two prominent distal lobes (Fig. 46A)	***T.digiticosta***
–	Distal margin of valva straight and without distal lobes (Fig. 45A)	***T.albidella***
8	Saccular process claviform	**9**
–	Saccular process not claviform	**10**
9	Saccular process with brush of dark bristles postmedially (Fig. 43A)	***T.meyricki***
–	Saccular process without brush of dark bristles postmedially (Figs 38A, 39A)	***T.salva***
10	Saccular process with strong bristles	**11**
–	Saccular process without strong bristles	**12**
11	Saccular process shorter, terminating postmedially (Fig. 41A)	***T.lacteella***
–	Saccular process longer, terminating pre-apically (Fig. 44A)	***T.thunbergella***
12	Saccular process with thick, long, straight anterior margin (Fig. 42A)	***T.madonna***
–	Saccular process with thin, shorter, curved anterior margin (Fig. 40A)	***T.albogrisella***

#### ﻿Key to species of *Athrypsiastis* based on external features

Note: Some members of this genus cannot be determined to species level without examination of genitalia (see 5 below).

**Table:** 

1	Antenna of male bipectinate	**2**
–	Antenna of male filiform	***A.penumbrella***
2	Ground colour white or off white	**3**
–	Ground colour yellowish ochreous with pink tinge	***A.phaeoleuca***
3	Terminal line present in forewing and hindwing	**4**
–	Terminal line not present in forewing and hindwing	**5**
4	Forewings and hindwings without dark brown points at end of veins	***A.delicata***
–	Forewings and hindwings with dark brown points at end of veins	***A.halmaherella***
5	Smaller species, wingspan 14 mm	***A.chionodes***
–	Larger species, wingspan ≥ 18 mm	***A.cheesmanae* , *A.edelweissella* , or *A.symmetra***

#### ﻿Key to species of *Athrypsiastis* based on male genitalia

**Table:** 

1	Aedeagus with small cornutus/i in vesica	**2**
–	Aedeagus without small cornutus/i in vesica	**5**
2	Aedeagus with single ridge-like cornutus in vesica	**3**
–	Aedeagus with small patch of short spines in vesica (Fig. 52B)	***A.penumbrella***
3	Saccular process strongly curved after base	**4**
–	Saccular process almost straight after base (Fig. 53A)	***A.symmetra***
4	Uncus tapering towards posterior apex (Fig. 50A)	***A* . *cheesmanae***
–	Uncus spatulate and broad at apex (Fig. 49A)	***A* . *phaeoleuca***
5	Saccular process not extending beyond costa of valva	**6**
–	Saccular process extending beyond costa of valva (Fig. 51A)	***A.edelweissella***
6	Valva uniformly broad throughout (Fig. 56)	***A.delicata***
–	Valva not uniformly broad throughout	**7**
7	Valva broad at base, elongate distally (Fig. 55)	***A.chionodes***
-	Valva narrow at base, broadening medially, not distally elongate (Fig. 54)	***A.halmaherella***

##### ﻿Genus Topiris

Taxon classificationAnimaliaLepidopteraXyloryctidae

Walker, 1863

9BC8E1F9-998D-585B-A2C3-68B7598E336C

Topiris Walker, 1863: 521. Type species Topiriscandidella Walker, 1863 by original monotypy.

###### Note.

*Topiris* is a genus which displays strong morphological characters in both male and female genitalia. We divide the genus into three informal groups based on characters in the male genitalia. Two of these groups are supported sub-clades within *Topiris*. All three groups exhibit constant and easily recognisable characters in the male genitalia.

###### Diagnosis.

Smallish xyloryctid moths, unicolourous and almost completely unmarked. In both sexes the labial palps are long (at least 2.5× diameter of the eye) and recurved (Figs 30, 31). R3 is present in the forewing, R3, R4, and R5 have a common stalk, and M3 and CuA1 are stalked (Figs 62, 64). This forewing venation distinguishes *Topiris* from other small whitish Oriental xyloryctids in *Deloryctis*, *Linoclostis*, *Metathrinca* and *Ptochoryctis* in which R3 is not present (Fig. 65). In the male genitalia of all species the aedeagus has a recurved, filament-like distal projection (long in the *salva* and the *albidella* groups, generally short in the *candidella* group) (Figs 68, 69). In the female genitalia the antrum is long, straight, narrow and sclerotised almost throughout (Fig. 72) and the corpus bursae is large and elongate with no signum (Fig. 73). Additionally, in the *candidella* group the valva has a large, sclerotised process at the base of the costal ventral membrane and the distal half of the valva is tapered (Figs 78, 80) and in the *albidella* group the gnathos is not fused medially and has two narrow lateral posterior projections and there is a small sclerite medially in the aedeagus (Figs 47, 70, 74). The forewing venation characters of *Topiris* are shared with *Athrypsiastis.* However, in the male genitalia, *Athrypsiastis* lack the filament-like distal projection of the aedeagus and the medial posterior projection of the gnathos strongly projects posteriorly and is strongly sclerotised, whereas the medial posterior projection of the gnathos in *Topiris* is weakly sclerotised and weakly projects posteriorly (Figs 75, 76), or is absent in the case of the *albidella* group (Figs 47, 74). *Topiris* are known to occur from Thailand to Sulawesi whereas *Athrypsiastis* are more easterly, known from Sulawesi to New Guinea.

###### Description.

**Adult. *Head***: Ocelli absent. Frons with appressed scales, vertex with a tuft of long, narrow scales projecting away from the base of the antennae, with further tufts of long scales projecting upwards from the occiput and/or from the sides of the occiput projecting inwards and posteriorly, overlaying a collar of broad lamellate scales projecting posteriorly from the anterior margin of the prothorax. Pilifers short and broad, bristled. Maxillary palps very small. Labial palps long (> 2.5× diameter of eye) and strongly recurved. Haustellum with basal half covered with silver-white scales. Antennae ¾ length of forewing, scape thickly scaled, no pecten, pedicel short and broad, flagellum in male with dark pectinations, reducing at ¾ with apical portion filiform, pectinations with small white sensillae; flagellum in female filiform throughout. ***Thorax***: Thorax with appressed lamellate scales. Tegulae short. Foreleg with tibial epiphysis. Tibial spurs 0–2–4. Hind legs with substantial tuft of long scales. Frenulum of male a single bristle from base of hindwing coupling with retinaculum under a scaled flap towards the base of Sc on the forewing. Forewing venation: R1 from ~ ½ discal cell, R3 present, R3, R4 and R5 with a common stalk, R3 pre-apical or to apex, R4 and R5 stalked, both post-apical. M1, M2 and M3 parallel and evenly spaced. M3 and CuA1 stalked. CuP present. Hindwing venation: Sc and Rs widely spaced, M2 and M3 sometimes closely approximated, M3 and CuA1 stalked (Figs 62, 64). Forewings broad, hindwings at least as broad. Dorsal surface of forewings unicolourous and usually without any markings except for a small line of dark brown scales at the edge of the base of the costa. In some species and forms there is a patch of thicker scaling on the dorsum, appearing greyish or brownish. The forewing cilia are without lines. The pre-genital abdomen is concolourous with forewings with patches of short thick orange-brown tergal spines pointing posteriorly on posterior part of T2–T7 and occasionally a small patch on T8 (Fig. 66).

***Male genitalia*.** Uncus anteriorly broad with anterior margin of dorsal surface weakly emarginate or almost straight, apical section spatulate or slightly bilobed (but tapering towards posterior apex in *albidella* group), small additional sclerites present in some members of *salva* group. Gnathos fused medially, with small, lightly sclerotised medial plate with single medial projection, weakly projecting posteriorly (Fig. 75) (*albidella* group: gnathos not fused medially with two narrow lateral posterior projections (Figs 47, 74)). Band of tegumen broad, strongly or moderately arched, lateral extensions of tegumen equal to or longer than width of tegumen band. Vinculum U shaped (but diverging strongly distad of saccus in *candidella* group (Fig. 77)), generally projecting substantially beyond base of valvae (but projecting weakly in *candidella* group). Saccus generally large (but small in *candidella* group). Anellus lobes broad. Valva with setose ventral membrane from costa (with a long, sclerotised setose process from the base in *candidella* group (Fig. 78)), basal projection of costal margin setose where present, apex of valva often with tuft of bristles, small elongate ventral sclerite near saccular margin, usually with a projection joining to base of saccular process. Sacculus large, longer than broad. Saccular process developing from distal part of sacculus, folded ventrad, large and distinctive, generally commencing close to costa of valva, straight towards base, generally without strong bristles apically. Aedeagus with recurved, filament-like distal process. Bulbus ejaculatorius long, generally two coils and a long hood.

***Female genitalia*.** Papillae anales short and broad. Apophyses posteriores longer than apophyses anteriores. Ostium small and circular. S8 variable. Antrum long straight and narrow, sclerotised almost throughout and also usually scobinate. Ductus bursae long and thin with contrasting membranous posterior and scobinate anterior sections. Corpus bursae large elongate, without signum.

###### Biology and early stages.

The biology and early stages of members of the genus are almost unknown, with the exception of *T.salva*, which (as a species of *Athrypsiastis*) has been reported as a pest of the genera *Citrus* (Rutaceae) and *Morus* (see account of *T.salva* for details). Also on the basis of the similarities in the COI of *T.salva* and the exemplar identified as “*Linoclostisgonatias*” for the mitogenome analysed by Su et al. 2020, we consider that the species there described as a pest of tea *Camelliasinensis* (L.) Kuntze and Oil-seed Camellia*C.oleifera* C. Abel (Theaceae) is *T.salva* and not *Linoclostisgonatias* Meyrick (for which Robinson et al. 2001: 246 cite *C.sinensis* as a host). Males of many of the species of *Topiris* have been collected at light. Females have been collected occasionally at light. *Topiris* have been found in lowland, mangrove, montane and secondary forest. The tectiform resting posture of *T.salva* is illustrated in Figs 30, 31, and in numerous pictures on iNaturalist (under *Athrypsiastissalva*) (https://www.inaturalist.org/observations?taxon_id=1391516) from China and Hong Kong.

###### Distribution.

The genus *Topiris* is known from China, Thailand, Indonesia (Borneo and Sulawesi), Malaysian Borneo (Sarawak), Brunei, and Peninsular Malaysia.

###### Status and conservation.

*Topirissalva* is the correct identity of the records of “*Linoclostisgonatias*” as a pest species in China and possibly Taiwan (Sonan 1939). Only *T.salva*, *T.schneeweissella*, and *T.* sp. RMNH.INS.20000 (BIN, BOLD:AAL9269) have been recorded post-2000.

###### Note on Walker’s etymology.

Francis Walker sometimes used ancient place names of no particular significance as generic names (Hoare 2010). ‘Topiris’ is referred to in classical literature as a town in the province of Thrace, situated near Abdera and the mouth of the river Nestus, Greece.

### ﻿The *candidella* group

In the male genitalia of this group the valva has a long, sclerotised, setose process from the base of the costal ventral membrane and the distal half of the valva is tapered (Figs 78, 80). In the female genitalia the posterior margin of S8 projects caudally and has two small digitate processes posterio-medially (Fig. 84).

#### ﻿ Topiris candidella

Taxon classificationAnimaliaLepidopteraXyloryctidae

Walker, 1863

45B02AE5-1170-537C-9292-453D8D500106

Figs 4–6 , 32A, B , 33A, B , 57 , 62 , 66 , 79 , 80 , 82 , 84–87

Topiris candidella Walker 1863: 522.

##### DNA barcodes.

BIN, BOLD:ADR9781 (Process IDs METAT027-18, METAT210-19, METAT211-19, METAT287-24).

##### Type material.

Malaysian Borneo: ***Holotype*** • ♂, Sarawak, Saunders Collection, fwl 7.5 mm, specimen no. NHMUK010219690 (labels: Fig. 85A, B). Holotype by monotypy.

##### Diagnosis.

Indistinguishable externally from other small white species and forms of *Topiris*. In the male genitalia, the postmedial section of the valva is narrower than that of *T.ochrotincta* and *T.schneeweissella.* In *T.cinderella* the setose costal ventral membrane is distally raised whereas in *T.candidella* it is distally flat and the process at the base of the costal ventral membrane arises at an acute angle in *T.cinderella* whereas in *T.candidella* the angle is more obtuse (Figs 81, 82). In the female genitalia, *T.albidella* and *T.salva* lack the digitate posterio-medial processes on S8 and in *T.schneeweissella* the antrum lacks scobination (Figs 57–60, 84).

##### Description.

**Figures 4–21.**
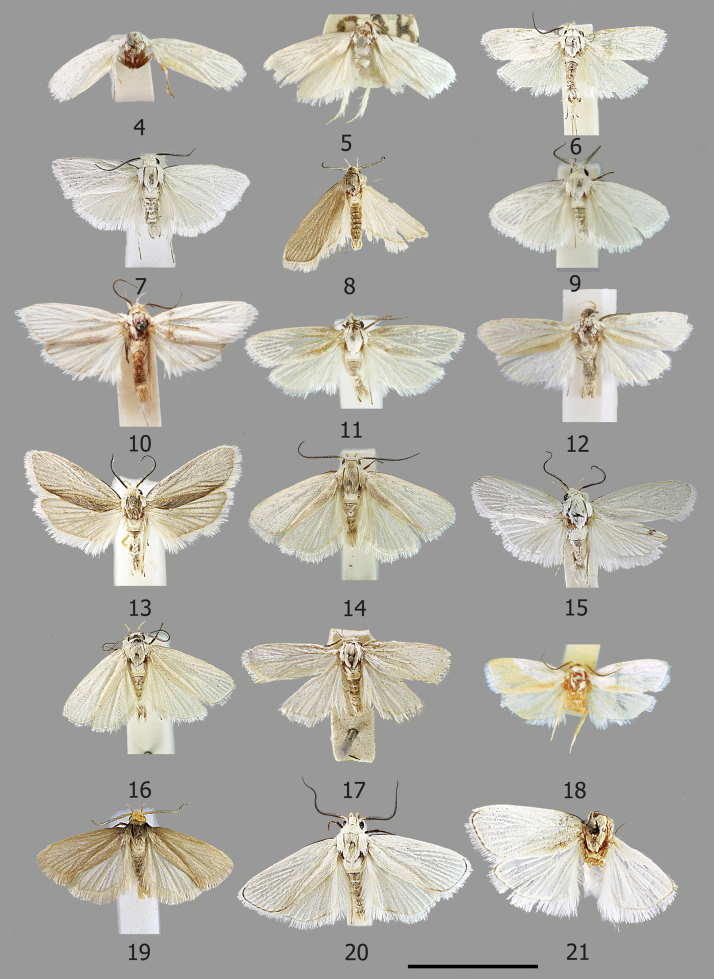
Dorsal images of *Topiris* spp. **4***Topiriscandidella* Walker, 1863, holotype ♂ **5***T.candidella*, Sarawak, leg. A.R. Wallace, ♂ **6***T.candidella*, Brunei, ♂ **7***T.cinderella* sp. nov., paratype ♂ **8***T.ochrotincta* sp. nov., holotype ♂ **9***T.schneeweissella* sp. nov., holotype ♂ **10***T.sericella* sp. nov., holotype ♂ **11***T.salva* (Meyrick, 1932), comb. nov., neotype ♂ **12***T.salva* (Meyrick, 1932), comb. nov., paraneotype ♂ **13***T.salva*, ♂ **14***T.albogrisella* sp. nov., holotype ♂ **15***T.lacteella* sp. nov., paratype ♂ **16***T.madonna* sp. nov., holotype ♂ **17***T.meyricki* sp. nov., holotype ♂ **18***T.sampitella* (Lvovsky, 2014), comb. nov., holotype ♂ **19***T.thunbergella* sp. nov., holotype ♂ **20***T.albidella* sp. nov., holotype ♂ **21***T.digiticosta* sp. nov., holotype ♂. Scale bar: 10 mm.

**Male** (Figs 4–6). Forewing length 6.5–7.5 mm, wingspan 14.5–16.5 mm. ***Head***: frons with pure white appressed scales; vertex with tuft of long white scales pointing upwards and away from base of antennae, further long pure white scales pointing posteriorly from sides of occiput and from posterior margin of occiput, overlaying a collar of appressed broad white scales pointing posteriorly from anterior margin of prothorax; maxillary palps white. Labial palps long (>2.5× diameter of eye), strongly recurved; small tuft of pale ochreous scales on basal segment; second segment longer than third, strongly curved, pale ochreous outer side, white on inner side; third segment slightly curved with appressed scales, ochreous mixed white. Haustellum with white scaling on basal part. Antenna ¾ length of forewing, bipectinate; scape with appressed scales white mixed ochreous; flagellum with dark pectinations for > ½ length covered with short white sensillae, ochreous scaling on dorsal surface of basal flagellomeres, otherwise dark brown, apical portion filiform. ***Thorax***: snow white; tegulae short, snow white; foreleg with femur white, tibia and tarsus brown, broad tibial epiphysis; mid and hind legs white, hind legs with short and broad white scale tuft. Forewing broad, costa slightly rounded at base, thereafter straight, apex obtusely rounded, termen slightly angled inwards, tornus obliquely angled, snow white, unmarked except for small line of brown scales from base of costa to 1/6. Hindwing as broad as forewing, apex very slightly projecting, white, unmarked. Ventrally, forewing with brown scaling in area between costa and Sc and pale brown scaling along veins; hindwings white.

**Female.** Similar to male, slightly larger, forewing length 9 mm, wingspan 19–20 mm, antenna filiform throughout.

***Pre-genital abdomen*** (Fig. 66). White, anal tuft white. Tergal spines on posterior parts of T2–T7, T8 weakly sclerotised anteriorly, sternites weakly sclerotised. Apodemes almost straight; venulae slightly sinuate.

***Male genitalia*** (Figs 32A, B, 33A, B, 79, 80, 82). Uncus broad, anterior margin of dorsal surface very weakly emarginate, apically slightly bilobed, almost rectangular, strongly sclerotised laterally. Gnathos fused medially, lateral arms thin and lightly sclerotised, medial plate lightly sclerotised, weakly projecting posteriorly from lateral arms. Tegumen band broad and strongly arched, lateral extensions of tegumen same length as width of tegumen band. Vinculum short, robust, strongly sclerotised, strongly diverging distad of saccus, U shaped basally, base slightly projecting anteriorly beyond base of valvae. Saccus short. Juxta with base plate strongly sclerotised, V-shaped, anellus lobes almost as broad as long. Valva long, basally broad, substantially tapering postmedially, apically narrow, costal ventral membrane confined to basal half of valva, rugose and with long thin setae, distally flat, from base of which arises, at an obtuse angle, a long, setose process, apex of valva thin and rounded with tuft of bristles, saccular margin curved, strong ventral sclerite postmedially, with projection joining to base of saccular process. Sacculus very large with strong mesad shoulder, slightly longer than broad. Saccular process developing from distal part of sacculus, commencing close to costa of valva, strongly sclerotised and melanised, broad and straight at base, narrowing towards apex, apical portion a shortish curved hook with a narrow apical point, short fine setae present. Aedeagus short, thin, slightly curved, small slightly recurved filament-like distal projection. Bulbus ejaculatorius long with elongate hood.

***Female genitalia*** (Figs 57, 84). Papillae anales short and broad. Apophyses posteriores longer than apophyses anteriores. S8 with anterior margin strongly recessed and strongly arched ventrad, posterior margin projecting caudally, covering the ostium, with two small digitate posterio-medial lateral processes. Ostium small and circular. Antrum long, straight and narrow, strongly sclerotised and melanised almost throughout, weakly scobinate. Ductus bursae long and thin, posteriorly membranous, anteriorly finely scobinate. Corpus bursae large and elongate, without signum.

##### Biology and early stages.

Early stages unknown. Adults have been recorded from mangrove forest, coastal swamp forest and kerangas forest at elevations of 0–20 m in January/February, April, and September/October. Wallace’s specimens are likely to have been found on a small hill overlooking rainforest at approximately 380 m elevation.

##### Distribution.

Sarawak (Malaysian Borneo), Brunei.

##### Additional material examined.

(18♂, 2♀) 1♂ Sarawak, Moore coll., 94–106, specimen number NHMUK013700123; • 1♂ Sarawak, Moore coll., 94–106, specimen number NHMUK013700125, slide no. NHMUK014331342; • 1♂ Sarawak, E.W. Janson coll. 74.69, specimen no. NHMUK013700124; • 1♂ 3km WSW of Muara, Kampong Kapok, edge of mangrove forest, 1 m, i–ii.1992, E.W. Classey leg., specimen no. NHMUK010922995, slide no. NHMUK010316360, Process ID METAT027-18; • 1♂ 3km WSW of Muara, Kampong Kapok, edge of mangrove forest, 1m, i–ii.1992, E.W. Classey leg., specimen no. NHMUK010923142, slide no. NHMUK014331341; • 4♂ 3 km WSW of Muara, Kampong Kapok, edge of mangrove forest, 1 m, i–ii.1992, E.W. Classey leg., specimen nos. NHMUK010922994, NHMUK013700122, NHMUK013700121, NHMUK013700120, 1♂ Brunei, 3 km WSW of Muara, Kg Kapok, edge of *Rhizophora* forest, 1 m, 21.ix.–3.x.1997, G.S. Robinson leg., specimen no. NHMUK010923170, slide no. NHMUK010316445, Process ID METAT210-19; • 1♂ Brunei, 3 km WSW of Muara, Kg Kapok, edge of *Rhizophora* forest, 1 m, 21.ix.–3.x.1997, G.S. Robinson leg., specimen no. NHMUK013700116; • 1♂, Brunei: 20 ft., Seria, coastal swamp forest, 11.ii.1982, G.S. Robinson leg., specimen no. NHMUK010923136, slide no.NHMUK014331343; • 2♂, Brunei: 20 ft., Seria, coastal swamp forest, 11.ii.1982, G.S. Robinson leg., specimen nos. NHMUK013700119, NHMUK013700118; • 1♂, Brunei: 10 ft., Seria, coastal swamp forest, 18.iv.1988, G.S. Robinson leg., specimen no. NHMUK013700117; • 2♂, Brunei: Telisai, Kerangas Forest, 10 ft., 8.iv.1988, G.S. Robinson leg., specimen nos. NHMUK013700115, NHMUK013700114; • 1♂, Sandakan, Br. N. Borneo, 9.ii.1893, Leg. Green, specimen no. NHMUK010219787, slide no. NHMUK014331344; • 1♀ Brunei, 3km WSW of Muara, Kg Kapok, edge of *Rhizophora* forest, 1 m, 13.ix–1.x.1992, G.S. Robinson leg., specimen no. NHMUK010923171, slide no. NHMUK010316864, Process ID METAT211-19; • 1♀ Telisai, Kerangas Forest, 10 ft., 8.iv.1988, G.S. Robinson leg., specimen no. NHMUK013700130, slide no. NHMUK014331340.

##### Remarks.

Both this species and *T.cinderella* have been found in Brunei. This species has been recorded principally from mangrove and other coastal forests whereas *Topiriscinderella* has only been found in dipterocarp forest. The pairwise divergence between these two species is 3.52–3.82%. *Topiriscandidella* is 4.74–5.05% pairwise divergent from *T.ochrotincta*, which has been found at altitude in Brunei, and 3.67–4.28% pairwise divergent from *T.schneeweissella*, which has been found in Peninsular Malaysia and Thailand.

The only differences in morphology between *T.cinderella* and *T.candidella* are in the distal part of the costal fold of the valva (illustrated in Figs 81, 82) and the angle of projection of the process at the base of the costal fold.

The derivation of Walker’s specific name, *candidella*, is from *candida* (lat.) white, innocent or pure.

The type specimen became chimaeric at the time that hindwings of another species were glued on this specimen. The hindwings are now separated from the holotype and separately mounted. We note that these hindwings lack a basal hyaline area and neither do they belong to the holotype nor to the genus *Yponomeuta* Latreille, [1796] (as suggested by Meyrick). It is not clear to which micromoth specimen or species the hindwings belong.

The data labels for the type specimen of *T.candidella* are illustrated in Fig. 85A, B. The specimen has a label which is a round white disc on which is written “SAR”. Walker’s original description states that the specimen is in Mr. Saunders’ collection, and this is also reflected by the NHMUK accession label, which states: Sarawak Saunders Coll. 94–68.

Alfred Russel Wallace visited Sarawak between November 1854 and January 1856 (Tuen and Das 2005). He spent his time in Sarawak as a guest of Rajah James Brooke. Between 13 December 1855 and 18 January 1856 (with a break for Christmas between 20 December and 30 December), he stayed at a bungalow owned by Rajah Brooke at Peninjau Hill (1.4293, 110.2236, 380 m elevation), near Kuching (Wallace 1869: 95–97; Moulton 1912; Idris and Azhar 2010). During his stay at the Peninjau bungalow, Wallace made a collection of a total number of 1386 moths. These specimens were collected by Wallace himself and labelled either by him or his assistant, Charles Allen (Wallace 1869). Although 100% confirmation is not possible, it is likely that Peninjau provided a high proportion of his total collection of Lepidoptera (Polaszek and Cranbrook 2006: 433, 434). Charles Allen placed circular white card labels with “SAR” for Sarawak on the specimens (Cranbrook and Mann 2016: 19; see also Baker 1996; Baker 2001). The *Topiriscandidella* label has been confirmed as definitively a Wallace label written by Charles Allen (George Beccaloni pers. comm. to DCL 14 November 2023). When Wallace returned to England in 1862 his insects, other than Coleoptera and butterflies, became part of the collection of William Wilson Saunders (Wallace 1869).

The three similar specimens of *T.candidella* subsequently found by DCL in the NHMUK collections (one of which is illustrated at Fig. 5) have similar white card disc labels bearing the letters “SAR” (Fig. 86). Wallace stated that the main object of his journeys in the Malay Archipelago was to obtain specimens of natural history, both for his private collection and to supply duplicates to museums and amateurs (Wallace 1869: xi). Wallace’s collections of insects were divided, before consignment, into (among other things) series reserved for his private collection and series intended for sale. Wallace records sending six consignments of insects from his stay in Sarawak (Baker 2001). We assume that the specimens which found their way into the collections of Janson and Moore were from Wallace’s sale specimens.

From this we conclude that the type (and the three subsequently discovered specimens) were collected by Alfred Russel Wallace during his stay in Sarawak and that it is most likely that they were taken at the Peninjau bungalow between 13 December 1855 and 18 January 1856.

*Topiriscandidella* is not listed in Polaszek and Cranbrook (2006) as an insect species described from Alfred Russel Wallace’s Sarawak collections, but this paper does not list microlepidoptera species.

Walker’s original description does not expressly state that it is made from a single specimen. However, his species description states: ‘in Mr. Saunders’ collection’. Two of the other three subsequently discovered specimens bearing Wallace’s collecting labels (NHMUK0137000123 and NHMUK0137000125) are labelled Moore Coll. (Fig. 86) and the third is labelled Janson collection. The accession dates for these specimens in each case post-date Walker’s description. Only one of Wallace’s specimens is from the Saunders collection. Baker (2001: 65) also notes that the Sarawak specimens acquired by Saunders were treated by Walker. We conclude that the example in the Saunders collection was the only specimen available to Walker when he wrote his description.

#### ﻿ Topiris cinderella

Taxon classificationAnimaliaLepidopteraXyloryctidae

Sterling & Lees sp. nov.

C74F1336-7359-5E70-87AA-0B784EFC7317

https://zoobank.org/3C24E83F-2A86-4DE7-8C22-8CC9AD59D3ED

Figs 7 , 34A, B , 69 , 77 , 81

##### DNA barcodes.

BIN, BOLD:ADZ9718 (Process IDs METAT222-19, METAT225-19, METAT247-10).

##### Type material.

Brunei: ***Holotype*** • ♂, Brunei, Bt. Bedawan, LP263, GR343958, ridge dipterocarp forest, 1700 ft., 20–24.iv.1988, G.S. Robinson leg., fwl 7 mm, specimen no. NHMUK010923201, slide no. NHMUK010316400, Process ID METAT247-19. ***Paratypes*** (9♂) 1♂ same collection data as holotype, specimen no. NHMUK013700355, slide no. NHMUK010316408; • 1♂ same collection data as holotype, specimen no. NHMUK010923200; • 1♂, Brunei, Rampayoh Road, 100 m, LP 195B, GR 960785, Lowland dipterocarp forest, 26–29.ix.1997, G.S. Robinson leg., specimen no. NHMUK010923182, slide no. NHMUK010316447, Process ID METAT222-19; • 1♂ Brunei, Rampayoh Road, LP291B GR951801, Lowland Dipterocarp Forest 11–15.iv.1988, G.S. Robinson leg., specimen no. NHMUK010923135, slide no. NHMUK010316446; • 2♂ Brunei, Rampayoh Road, LP291B, GR951801, Lowland Dipterocarp Forest 11–15.iv.1988, G.S. Robinson leg., specimen nos., NHMUK013700113, NHMUK013700112; • 1♂, Brunei, Lamunin, Sg Burong, disturbed lowland forest, 60 m, 15–20.iv.1993, G.S. Robinson leg. specimen no. NHMUK010923185, slide no. NHMUK010316448, Process ID METAT225-19; • 1♂ Brunei, Rampayoh R., LP291B, GR951801, lowland dipterocarp forest, 150 m, 11–15.iv.1988, G.S. Robinson leg., specimen no. NHMUK010923135; slide no. NHMUK010316446; • 1♂ Brunei, Ulu Temburong, Kuala Belalong FSC, lowland dipterocarp forest, 100 m, 6.vii.1991, G.S. Robinson leg., specimen no. NHMUK010923133, slide no. NHMUK010315368.

##### Diagnosis.

Externally indistinguishable from other small white species and forms of *Topiris*. The comparatively thin postmedial section of the valva and shorter bristles at the apex distinguishes this species from *T.ochrotincta* and *T.schneeweissella*. The distinctions in the male genitalia between *T.cinderella* and *T.candidella* are given in the diagnosis of *T.candidella*.

##### Description.

**Male** (Fig. 7). Forewing length 7–7.5 mm, wingspan 15–17 mm. ***Head***: frons with silver white appressed scales; vertex with a small tuft of silvery white scales pointing upwards and away from the base of antennae, thick tuft of long white lamellate scales pointing inwards and posteriorly from sides of occiput, overlaying collar of broad white scales pointing posteriorly from anterior margin of prothorax; pilifers small with small tufts of dark bristles; maxillary palps white. Labial palps long (> 2.5× diameter of eye), strongly recurved; basal segment with small scale tuft; second segment longer than third, strongly curved, thinly scaled with pale ochreous and white scales; third segment almost straight, thinly scaled ochreous white. Haustellum with basal portion scaled white. Antenna ¾ length of forewing, bipectinate; scape silvery white with some ochreous scaling towards pedicel, flagellum with short broad dark pectinations for ¾ of its length covered in short white sensillae, ochreous on dorsal surface for a short distance at base, thereafter dark brown, apical portion filiform. ***Thorax***: covered in snow white lamellate scales, tegulae snow white; foreleg with femur white, tibia and tarsus brown, moderately broad tibial epiphysis, mid and hindlegs white, hindleg with tuft of long white scales. Forewing broad, costa slightly rounded at base, otherwise straight, apex slightly projecting, termen angled slightly inwards, tornus obtusely angled, snow white with no markings except for a small line of dark brown scales at base of costa to ~ 1/5. Hindwing as broad as forewing, very slightly pointed at apex otherwise round and broad, white with no markings. Ventrally, surface of forewing with area between costa and Sc with brown scaling and ochreous scaling along the veins, hindwing white.

**Female**. Unknown.

***Pre-genital abdomen*.** White, anal tuft white. Tergal spines on posterior parts of T2–T7, T8 weakly sclerotised anteriorly; sternites weakly sclerotised. Apodemes almost straight, venulae sinuate.

***Male genitalia*** (Fig. 34A, B). Uncus broad, anterior margin of dorsal surface weakly emarginate, apically slightly bilobed. Gnathos fused medially, lateral arms thin and lightly sclerotised, medial plate small and lightly sclerotised, weakly projecting posteriorly from lateral arms. Tegumen band broad, strongly arched, lateral extensions of tegumen same length as width of tegumen band. Vinculum robust, strongly sclerotised, strongly diverging distad of saccus (Fig. 77), U shaped basally, base barely projecting anteriorly beyond base of valvae. Saccus short. Juxta with base plate strongly sclerotised, anellus lobes broad. Valva long, basally broad, substantially tapering apically, costal ventral membrane confined to inner half of valva, rugose, raised distally (Fig. 81), bearing long fine setae, from base of which arises, at an acute angle, a long, sclerotised process which is setose throughout, apex thin and rounded with apical tuft of bristles, saccular margin curved, strong ventral sclerite postmedially with projection joining to base of saccular process. Sacculus very large with strong mesad shoulder, slightly longer than broad. Saccular process developing from distal part of sacculus, commencing close to costa of valva, strongly sclerotised and melanised, broad and straight at base, narrowing towards apex, apical portion a long, curved hook with a narrow apical point, short fine setae present. Aedeagus short, thin, slightly curved, small recurved, filament-like distal projection (Fig. 69). Bulbus ejaculatorius long with two coils and a broad elongate hood.

##### Biology and early stages.

Early stages unknown. Adults have been found in dipterocarp forest in April, July, and September.

##### Distribution.

Brunei.

##### Etymology.

*cinderella* — named after the heroine of the fairy tale of that name. A closely related species to Snow White (see below, *T. schneeweissella*). The epithet is a noun in apposition.

#### ﻿ Topiris ochrotincta

Taxon classificationAnimaliaLepidopteraXyloryctidae

Sterling & Lees sp. nov.

E240DE12-1408-5EE6-9BC1-A3043200C861

https://zoobank.org/3F90C1FD-ED48-44F9-833B-C81FE01A34E7

Figs 8 , 35A, B , 78

##### DNA barcode.

BIN, BOLD:ADZ9719 (Process ID METAT209-19).

##### Type material.

Brunei: ***Holotype*** • ♂, Brunei, Bukit Pagon, LP308, upper montane forest, 1800 m, 15–20.ii.1982, G.S. Robinson leg., fwl 9 mm, specimen no. NHMUK010923169, slide no. NHMUK010316401, Process ID METAT209-19.

##### Diagnosis.

This is the only described member of the *candidella* group which is not snow white in colour although *Topiris* ‘RMNH 20000’ is a similar colour. It is also slightly larger than other species within the group. In the male genitalia the postmedial part of the valva is broader than *T.candidella* or *T.cinderella* but is of similar width to *T.schneeweissella*. The saccular process of *T.ochrotincta* is shorter and the apical hook of the saccular process is shorter and broader than that of *T.schneeweissella*.

##### Description.

**Male** (Fig. 8). Forewing length 9 mm. ***Head***: frons with dark cream appressed scales; vertex with tuft of long pale ochreous scales pointing upwards and away from base of antennae, further long pale ochreous scales pointing posteriorly from sides of occiput, long greyish ochreous scales pointing posteriorly from posterior margin of occiput, overlaying a collar of broad lamellate greyish ochreous scales pointing posteriorly from anterior margin of prothorax; pilifers small with moderate tufts of bristles. Labial palps long (~ 2.5× diameter of eye), strongly recurved; small tuft of dirty cream scales on basal segment; second segment strongly curved, thinly covered with appressed dirty cream scales; third segment shorter than second, slightly curved with a thin covering of dirty cream scales. Haustellum with cream scales on basal portion. Antenna ¾ length of forewing, bipectinate; scape dark cream, flagellum with short broad pectinations covered with short white sensillae for ~ ¾ of length, apical portion filiform, dark cream scaling of the anterior margin of the dorsal surface of flagellum for most of length, otherwise dark brown. ***Thorax***: covered in pale greyish ochreous appressed scales; tegulae short, same colour as thorax; foreleg with some white scaling on femur, tibia and tarsus pale brown, moderately long tibial epiphysis, mid legs and hind legs white, hind legs with long tuft of pale cream scales. Forewing broad, costa slightly curved at base, thereafter straight, apex slightly pointed, termen angled slightly inwards, tornus obtusely rounded, pale ochreous, unmarked except for a faint line of dark brown scales from costa to ~ 1/5, cilia silvery white with some pale ochreous reflections. Hindwing as broad as forewing, rounded with a slight projection at apex, silvery white unmarked. Ventrally, surface of forewing with costa ochreous, area between costa and Sc with dark brown scaling and brown scaling along veins, hindwing pale grey.

**Female**. Unknown.

***Pre-genital abdomen*.** Very pale ochreous. Tergal spines on posterior parts of T2–T7, T8 weakly sclerotised anteriorly; sternites weakly sclerotised.

***Male genitalia*** (Fig. 35A, B). Uncus broad, anterior margin of dorsal surface weakly emarginate, narrowing medially, apically slightly spatulate. Gnathos fused medially, lateral arms thin and lightly sclerotised, medial plate small and lightly sclerotised, weakly projecting posteriorly from lateral arms. Tegumen band broad and moderately arched; lateral extensions of tegumen longer than width of tegumen band. Vinculum short, robust, strongly sclerotised, diverging strongly distad of saccus, U shaped basally, base slightly projecting anteriorly from base of valvae. Saccus short. Juxta with a heavily sclerotised broad basal plate, anellus lobes broad. Valva long, broad at base, tapering gradually to moderately narrow rounded apex, costal ventral membrane confined to inner half of valva, rugose, sclerotised and with long setae, from base of which arises a long, sclerotised process which is setose throughout (Fig. 78), apex of valva with tuft of bristles which are long towards costal margin, saccular margin of valva slightly curved, strong ventral sclerite postmedially with projection joining to base of saccular process. Sacculus very large with strong mesad shoulder, slightly longer than broad. Saccular process developing from distal part of sacculus, commencing near costa of valva, broad and straight at base, narrowing towards apex, apical portion a relatively short, broad, curved hook with a narrow apical point, short fine setae present. Aedeagus short, thin, slightly curved, small, recurved, filament-like, distal projection. Bulbus ejaculatorius long, two coils, broad elongated head.

##### Biology and early stages.

Early stages unknown. Adult found in upper montane forest in Brunei in February.

##### Distribution.

Brunei.

##### Etymology.

*ochrotincta* — from *ochros* (gr.), ochreous yellow; *tinctus* (lat.), dyed; from the pale ochreous tinge to the whitish forewings of this species. The epithet is an adjective in the nominative singular.

##### Remarks.

This species is 2.14% pairwise divergent from *T.cinderella.* Both species have been found in Brunei. However, the forewings of *T.ochrotincta* are a different colour from *T.cinderella* and the specimen was found in upper montane forest whereas *T.cinderella* has been found in dipterocarp forest at lower levels. Also, in the male genitalia of *T.ochrotincta*, the postmedial section of the valva is broad compared to *T.cinderella* and the apical part of the saccular process is shorter and more acutely hooked than that of *T.cinderella*.

#### ﻿ Topiris schneeweissella

Taxon classificationAnimaliaLepidopteraXyloryctidae

Sterling & Lees sp. nov.

D892D676-9844-5960-B07B-EE9F9E624773

https://zoobank.org/F70E5491-F603-4C64-940A-4D18ADC9726F

Figs 9 , 36A, B , 58 , 75 , 83

##### DNA barcodes.

BIN, BOLD:ADR9774 (Process IDs METAT031-18, METAT214-19).

##### Type material.

Peninsular Malaysia, Thailand. ***Holotype*** • ♂, W. Malaysia, Selangor, Bangi, UKM campus, lowland dipterocarp forest, 70 m, 25–26.vii.1991, G.S. Robinson leg., fwl 6 mm, specimen no. NHMUK010922999, slide no. NHMUK010316358, Process ID METAT031-18. ***Paratypes*** (7♂, 1♀): • 1♂ same collection details as holotype, specimen no. NHMUK010923152, slide no. NHMUK014331345; • 1♂ Malaysia, West Pahang, Genting Tea Estate, 2000ft, 11–29.11.1981, H.S. Barlow leg., specimen no. NHMUK010923173, slide no. NHMUK010316449, Process ID METAT213-19 (307 bp); • 1♂ W. Malaysia, Trengganu, 12 km S of Kuala Dungun, Bukit Bauk, dry lowland dipterocarp forest, 100 m, 3.viii.1991, G.S. Robinson leg., specimen no. NHMUK010219698; slide no. NHMUK010316450; • 1♂, Malaysia, West Pahang, Genting Tea Estate, 610 m, 10.x.1976, H.S. Barlow leg., specimen no. NHMUK013699873, slide no. NHMUK013691346; • 1♂, Malaysia, West Pahang, Genting Tea Estate, 610 m, 01.01.1982, H.S. Barlow leg., specimen no. NHMUK013700111; • 1♂ S. Thailand, Narathiwat, km 17 on Ban Tabing Tingngi to Sri Sakhon Rd., 3.xii.1991, I.J. Kitching & A.M. Cotton leg., specimen no. NHMUK010219804, slide no. NHMUK014331347; • 1♂, Thailand, Narathiwat km 17, Ban Tabing Tingngi, to Sri Sakhon road, 220 m, 13.viii.1990, I.J. Kitching & A.M. Cotton leg., specimen no. NHMUK013700110; • 1♀ Malaysia, West Pahang, Genting Tea Estate, 2000ft, 29.11.1994, G.S. Robinson leg., specimen no. NHMUK010923174, slide no. NHMUK010316451, Process ID METAT214-19.

##### Diagnosis.

Indistinguishable externally from other white species and forms of *Topiris*. In the male genitalia the broader postmedial section of the valva (Fig. 83) distinguishes this species from *T.candidella* and *T.cinderella.* Its snow-white colour and longer, less hooked, apical part of the saccular process distinguishes it from *T.ochrotincta* (Figs 9, 36). In the female genitalia the antrum lacks scobination (Fig. 58).

##### Description.

**Male** (Fig. 9). Forewing length 5.5–8 mm, wingspan 12.5–17.5 mm. ***Head***: frons with appressed silver white scales; vertex with tuft of pure white scales pointing upwards and away from base of antennae, a thin tuft of long white scales from sides of occiput pointing upwards and a ruff of long white scales pointing posteriorly from posterior margin of occiput, overlaying a collar of broad flat white scales from anterior margin of prothorax; pilifers small, cylindrical with short tufts of bristles; maxillary palps white. Labial palps strongly recurved, long (> 2.5× diameter of eye), first segment with small white scale tuft, second segment longer than third, strongly curved, thinly scaled ochreous mixed with some white, third segment almost straight, thinly covered with white scales. Haustellum with basal portion scaled silver white. Antenna ¾ length of forewing, bipectinate; scape with appressed white scaling at base, ochreous scaling towards pedicel, flagellum with shortish broad dark pectinations covered with short white sensillae for ¾ of length, ochreous scaling on dorsal surface for ¼ of length, thereafter dark brown, apical portion filiform. ***Thorax***: white lamellate scales, tegulae short, white; foreleg with femur silver white, tibia and tarsus brown, long tibial epiphysis; mid and hind legs white, hind legs with thick tuft of white scales. Forewing broad, costa slightly rounded at base, very slightly convex thereafter, apex obtusely rounded, termen angled slightly inwards, tornus obtusely angled; snow white, slightly iridescent, unmarked except for a line of dark brown scales from edge of base of costa to ~ 1/5. Hindwing slightly broader than forewing, apex slightly projecting, white, unmarked. Ventrally, surface of forewings dark cream, veins scaled dark cream; hindwing white.

**Female.** Similar to male except forewing length 8 mm, wingspan 17.5 mm; antennae filiform throughout; thicker scale patch on dorsum tinged dirty white.

***Pre-genital abdomen*.** White, long white anal tuft, patches of tergal spines on posterior parts of T2–T7, T8 with slight sclerotisation, apodemes almost straight, moderately long, venulae slightly sinuate.

***Male genitalia*** (Figs 36A, B, 83). Uncus broad, anterior margin of dorsal surface weakly emarginate, apex slightly bilobed. Gnathos fused medially, lateral arms thin and lightly sclerotised, medial plate small and lightly sclerotised, weakly projecting posteriorly from lateral arms (Fig. 75). Tegumen band broad and strongly arched, lateral extensions of tegumen same length as width of tegumen band. Vinculum short, robust, well sclerotised, strongly diverging distad of saccus, U shaped basally, base of vinculum barely projecting anteriorly beyond base of valvae. Saccus short. Juxta with broad V shaped basal plate, anellus lobes broad. Valva (Fig. 83) moderately long, broad at base, tapering to moderately narrow rounded apex, costal ventral membrane confined to inner half of valva, slightly rugose, long fine setae, from base of which arises a long, sclerotised process which is setose throughout, apex of valva with tuft of bristles which are long on costal margin, saccular margin of valva slightly curved, broad ventral sclerite postmedially. Sacculus very large with strong mesad shoulder, slightly longer than broad. Saccular process developing from distal part of sacculus, commencing close to costa of valva, strongly sclerotised and melanised, broad and straight at base, narrowing towards apex, apical part a long, curved hook terminating in a sharp apical point, short fine setae present. Aedeagus short, thin, slightly curved, small slightly recurved filament-like distal projection. Bulbus ejaculatorius long, coiled, elongate hood.

***Female genitalia*** (Fig. 58). Papillae anales short and broad. Apophyses posteriores longer than apophyses anteriores. S8 with posterior margin projecting caudally, covering the ostium, with two small digitate posterio-medial processes, anterior margin strongly recessed and strongly arched ventrad. Ostium small, circular. Antrum long, straight, and narrow, well sclerotised almost throughout. Ductus bursae long and thin, posteriorly membranous, anteriorly finely scobinate. Corpus bursae large and elongate, without signum.

##### Biology and early stages.

Early stages unknown. Adults have been found in lowland dipterocarp forest in West Malaysia and Southern Thailand in July, August, October, November, and December at elevations of 70–600 m. Males and one female collected at mercury vapour light.

##### Distribution.

West Malaysia, Southern Thailand.

##### Etymology.

*schneeweissella* — from *schnee*, snow; and *weiss*, white (German). This is a reference to the glistening snow white colour of the wings. The epithet is an adjective in the nominative singular.

#### ﻿ Topiris sericella

Taxon classificationAnimaliaLepidopteraXyloryctidae

Sterling & Lees sp. nov.

6E6EFC4D-6561-5384-8A7E-90266FC4F0D8

https://zoobank.org/9F3639FC-00F2-4B3B-A8E6-9B488A6C128F

Figs 10 , 37A, B

##### DNA barcode.

N/A.

##### Type material.

Philippines: ***Holotype*** • ♂, Taytay, Palawan. plains. 22.iv.1913, A.E. Wileman leg., fwl 8.5 mm, specimen no. NHMUK010219786, slide no. NHMUK010316873. ***Paratype*** • ♂, same collection data as holotype, specimen no. NHMUK010219788, slide no. NHMUK010316878.

##### Diagnosis.

The hindwings of this species are slightly grey towards the termen and have an indistinct greyish terminal line. In the male genitalia, the vinculum is almost straight above the saccus, the valvae are postmedially narrow and uniformly sclerotised, the costal ventral membrane of the valva is smooth, almost without setae and with a substantial, strongly curved, sclerotised process arising from its base. The aedeagus is long and has a long filament-like distal projection with a small distal thickening.

##### Description.

**Male** (Fig. 10). Wingspan 18–19 mm, forewing length 8–8.5 mm. ***Head***: frons with shining white appressed scales; vertex with white appressed scales, two tufts of long white scales laterally on vertex pointing inwards and posteriorly, remains of tuft of long white scales on posterior part of occiput, overlaying a thick collar of broad white and pale ochreous scales from anterior margin of prothorax; pilifers cylindrical with small tuft of short bristles; maxillary palps whitish. Labial palps long (almost 3× diameter of eye), strongly recurved, white, projecting away from head; basal segment pale ochreous; second segment long, strongly curved; third segment long and thin with white and some pale ochreous appressed scales. Haustellum with basal third scaled silver white. Antenna 2/3 length of forewing, bipectinate, scape ochreous white, flagellum dorsally ochreous white for a few segments, otherwise dark brown, pectinations black, densely covered with short white sensillae, reducing at ¾, apical portion filiform. ***Thorax***: (worn) ochreous white mixed with white, tegulae shining silver white; foreleg with femur white, long thin tibial epiphysis, tibia and tarsus greyish brown, mid leg ochreous whitish, hind leg white with thin tuft of long white scales. Forewing moderately broad, costa slightly arched towards base, thereafter straight, apex broadly rounded, termen angled inwards, tornus very obtusely angled, shining silver white, unmarked except for a small line of brown scales from base of costa to 1/5, a patch of thicker silver scaling along length of dorsum on one specimen, cilia long, silver white. Hindwing rounded with apex slightly projecting; silvery white but with some darker tipped scales, particularly towards the termen giving a slightly greyish appearance and with indistinct greyish terminal line. Ventrally, surface of forewing dark brown towards costa, otherwise pale greyish ochreous, veins pale greyish ochreous; hindwings white.

**Female.** Unknown.

***Pre-genital abdomen*.** White, long white anal tuft. Tergal spines on posterior part of T2–T7, T8 almost unsclerotised, sternites weakly sclerotised. Apodemes almost straight; venulae slightly curved.

***Male genitalia*** (Fig. 37A, B). Uncus broad, anterior margin of dorsal surface very weakly emarginate, slightly narrowing medially, apically almost rectangular, slightly spatulate. Gnathos fused medially, lateral arms thin and lightly sclerotised, medial plate small and lightly sclerotised, weakly projecting posteriorly from lateral arms. Tegumen band broad and strongly arched, lateral extensions of tegumen longer than width of tegumen band. Vinculum large and robust, U shaped, arms parallel distad of saccus, base significantly projecting anteriorly beyond base of valvae. Saccus large. Juxta with small sclerotised basal plate, anellus lobes broad. Valva long, broad at base, strongly sclerotised postmedially and strongly tapering apically, apex of valva thin and rounded with tuft of short bristles, costal ventral membrane confined to basal half of valva, surface smooth and with few setae, long curved sclerotised process arising from base of ventral membrane from costa, broad at base, no setae apart from brush of shortish hairs at apex, saccular margin of valva slightly convex, strong ventral sclerite postmedially. Sacculus large, well sclerotised, longer than broad. Saccular process commencing near costa of valva, strongly sclerotised, broad and straight at base, short and hooked, terminating in a sharp point, no setae or bristles. Aedeagus long, slightly recurved with undulating distal filament-like projection of sheath and small, pointed, distal thickening. Bulbus ejaculatorius long with two coils and a large, elongated hood.

##### Remarks.

The male genitalia of this species displays some of the characteristic features of the *salva* group (e.g., aedeagus with long filament-like distal projection and with a distal thickening and vinculum not diverging strongly distad of saccus) and some of the characteristics of the *candidella* sub-clade (e.g., costal ventral membrane with long sclerotised process arising from base and valva tapering apically). The combined analysis (Fig. 2, which contains morphological data only from *T.sericella*) shows that *T.sericella* + the *T.candidella* sub-clade form a supported sub-clade (pp = 0.98) and we place *T.sericella* in the *candidella* group.

##### Biology and early stages.

Early stages unknown. Adults found in April.

##### Distribution.

Known from Palawan, Philippines.

##### Etymology.

*sericella* — from *sericus* (lat.), silky. A reference to the silky white gloss on the forewings. The epithet is an adjective in the nominative singular.

### ﻿The *salva* group

This group does not form a supported sub-clade within *Topiris*. However, members of the group can be distinguished in the male genitalia by the combination of the gnathos, which is fused medially, the valva which is broad throughout, and the aedeagus with a long, recurved, filament-like distal process and small distal thickening.

#### ﻿ Topiris salva

Taxon classificationAnimaliaLepidopteraXyloryctidae

(Meyrick, 1932) comb. nov.

3F7F5D9B-5850-5E4C-BB1A-A38CEDF12C30

Figs 11–13 , 30 , 31 , 38A, B , 39A, B , 59 , 64 , 72 , 73

Athrypsiastis salva Meyrick, 1932 (Meyrick in Caradja 1931–1933: 158).

##### DNA barcodes.

BIN, BOLD:ADS0105 (METAT020-18, METAT021-18, METAT022-18, METAT024-18, METAT025-18); MT547768 (whole mitogenome).

##### Type material.

***Type*.** Meyrick designated a type specimen but this is lost (see Discussion). ***Neotype*** • ♂ Hong Kong, Victoria Peak, July 1993, 400 m, leg. Kent Li, specimen no. NHMUK010922992, slide no. NHMUK014331348, Process ID METAT020-18. ***Paraneotype*** • ♂ Hangchow (= Hangzhou), China. JFC 5.7.25, specimen no. NHMUK010219696, slide no. NHMUK010316441.

##### Diagnosis.

This species has both all white forms and forms which are off white with darker thickened scaling dorsally (Figs 11–13). In Hong Kong both these forms occur together at the same time of year. In the male genitalia, *T.salva* has a broad, claviform saccular process. The only other species which has a similarly shaped saccular process is *T.meyricki*. The saccular process of *T.salva* is narrower than that of *T.meyricki* and lacks the patch of short bristles postmedially. In *T.salva* the apical part of the valva is rounded whereas in *T.meyricki* the distal margin of the valva is strongly emarginate with a large, digitate projection apically and a smaller sclerotised thorn like projection beneath this (Figs 38A, 39A, 43A). In the female genitalia, the posterior margin of S8 is projected caudally, covering the ostium, but lacks the posterio-medial projections of *T.candidella* and *T.schneeweissella* (Figs 72, 84).

##### Description.

**Male** (Figs 11–13). Forewing length 6.5–8 mm, wingspan 14–16 mm. ***Head***: frons with appressed silver white scales; vertex with long narrow cream scales projecting away from the base of the antennae with further such scales projecting posteriorly over occiput, overlaying a collar of broader white appressed scales projecting posteriorly from anterior margin of prothorax; pilifers small with tufts of moderately long bristles; maxillary palps white. Labial palps strongly recurved, long (3× diameter of eye); basal segment with small white scale tuft; second segment longer than third, strongly curved with thick covering of appressed silver white scales; third segment long, almost straight, thinly covered in appressed silver white scales. Haustellum with silver white scaling in basal portion. Antenna ¾ length of forewing, bipectinate; scape with appressed cream and white scales, flagellum with short dark pectinations covered in short white sensillae for ¾ length, silver scaling on dorsal surface basally, thereafter dark brown, apical portion filiform. ***Thorax***: with broad silver white scales; tegulae silver white; foreleg with femur silver white, tibia and tarsus brown with silver reflections in some lights, tibial epiphysis long and broad, mid legs and hind legs white with thin tufts of long white scales. Forewing broad, costa gently rounded at base, otherwise straight, apex slightly pointed, termen angled inwards, tornus obtusely angled, costa gently rounded at base otherwise straight, apex slightly pointed, termen angled inwards, tornus obtusely angled, silver white to off white, some forms entirely unmarked and silver white except for a line of brown scales from costa to ~ 1/6, in other forms where the ground colour is off white, the thicker scaling on the dorsum is tinged greyish ochreous or grey, forming an indistinct coloured patch. Hindwing as broad as forewing, rounded, white, unmarked. Ventrally, forewings pale brown, veins lined with brown scales, base of costa dark; hindwings white.

**Female.** Similar to male but more likely to have obscure colouration in the dorsal patch and slightly larger, forewing length 8.5–10 mm, wingspan 18–22 mm; antennae filiform throughout.

***Male genitalia*** (Figs 38A, B, 39A, B). Uncus broad, anterior margin of dorsal surface very weakly emarginate, two small lateral carinae basally, apically spatulate, apex rounded. Gnathos fused medially, lateral arms thin and lightly sclerotised, medial plate small and lightly sclerotised, weakly projecting posteriorly from lateral arms. Tegumen band broad and strongly arched, lateral extensions of tegumen substantially longer than width of tegumen band. Vinculum long, robust, U shaped, base substantially projecting anteriorly beyond base of valvae. Saccus large. Juxta with U-shaped basal plate, anellus lobes broad and moderately short. Valva long and broad, apex rounded, costal margin substantially projecting inwards and setose at base, costal ventral membrane long and broad with substantial long dark setae, saccular margin of valva slightly curved, long ventral sclerite postmedially, apex of valva with tuft of short bristles. Sacculus as broad as valva and longer than broad. Saccular process developing from distal part of sacculus, commencing near costa of valva, broad, claviform, one or two small sclerotised teeth on upper margin. Aedeagus long with a long, recurved, filament-like distal projection and a small distal thickening. Bulbus ejaculatorius long, two coils, hood broad.

***Female genitalia*** (Figs 59, 72, 73). Papillae anales short and broad, apophyses posteriores substantially longer than apophyses anteriores. Ostium small and circular. S8 with posterior margin projecting caudally and covering ostium, anterior margin almost straight. Ostium small and circular. Antrum long, straight, narrow, sclerotised and scobinate almost throughout. Ductus bursae long and thin, posteriorly membranous, anteriorly finely scobinate. Corpus bursae large and elongate, without signum.

***Pre-genital abdomen*.** White with white anal tuft. Tergal spines on posterior parts of T2–T7, visible on dried specimens; weak sclerotisation of anterior part of T8 and sternites. Apodemes long and straight, venulae slightly sinuate.

##### Biology and early stages.

*Topirissalva* (as a species of *Athrypsiastis*) has been reported as a pest of *Citrus* L. (CASI 1994; Li et al. 1997) and Mulberry (*Morus* sp.) (Ertian 2003: 397). This species also appears to have been misidentified in Han et al. (2007) and Su et al. (2020) as *Linoclostisgonatias*. The species described in that literature is a pest of tea (*C.sinensis*) and *C.oleifera* (Theaceae), the larva boring into the shoots. Male adults, and less frequently females, attracted to lights at night. Found recently in secondary woodland and at the edge of a village adjoining secondary woodland. Adults have been found in May, July, and September. The adult has a strongly tectiform resting posture (Figs 30, 31).

##### Distribution.

China. The type and Meyrick’s other two specimens were from Shanghai and Hangzhou. Though misidentified, this is the species which has been reported (see paragraph above) as a pest in China. It is also widely distributed in Hong Kong.

##### Additional material examined

**(12**♂, **4**♀) . • 1♂ Nam Chung Valley, Hong Kong, China, 22.512N, 114.21E, 11 May 2017, M. Sterling leg., specimen no. NHMUK010219679, slide no. NHMUK010316399, MSterling043, Process ID METAT023-18 (307 bp); • 1♂ Hong Kong, Victoria Peak, viii.1993, leg. A. Galsworthy, specimen no. NHMUK010923140, slide no. NHMUK014331349; • 1♂ Hong Kong, Tai Po Kau Headland, New Territories, 22.436N 114.192E, 65 m, 06 May 2017, leg. M.J. Sterling, specimen no. NHMUK013700108, slide no. NHMUK014331350, MSterling044, Process ID METAT024-18; • 1♂ Hong Kong, Tai Yeung Che, Tai Po, New Territories, 22.447N 114.128E, 65 m, 5 May 2017, leg. M.J. Sterling, specimen no. NHMUK013700109, slide no. NHMUK014331351; • 2♂ Hong Kong, Victoria Peak, May 1993, leg. A. Galsworthy, specimen nos. NHMUK010922993, NHMUK013700107; • 1♂ Hong Kong Tai Yeung Che, Tai Po, New Territories, 22.447N 114.128E, 65 m, 1 May 2017, leg. M.J. Sterling, specimen no. NHMUK013700106, MSterling042, Process ID METAT022-18; • 1♂ Hong Kong, Ng Tung Chai, N. Territories, 22.436N 114.124E, 135 m, 8 June 2018, leg. M.J. Sterling, specimen no. NHMUK010219723, slide no. NHMUK014331358; • 2♂ Hong Kong, Tai Mong Sai, Sai Kung, 22.405N 114.303E, 140 m, 24 April 2013, M.J. Sterling leg., specimen nos. NHMUK013700104, NHMUK013700105; • 1♂ Hong Kong, Tai Tam, Hong Kong Island, 20 m, 10.09.1995, leg. M.J. Sterling, specimen no. NHMUK013700103; • 1♂ Hong Kong, Ng Tung Chai, N. Territories, 22.436N 114.124E, 135 m, 2 May 2017, leg. M.J. Sterling, specimen no. NHMUK013700102; • 1♀ Hong Kong, China, Kadoorie Institute, New Territories, 22.428N 114.113E, 200 m, leg. M.J. Sterling, specimen no. NHMUK010219702, slide no. NHMUK014331352, MSterling045, Process ID METAT025-18; • 1♀ Hangchow (= Hangzhou), China. JFC 5.7.25, specimen no. NHMUK010219725, slide no. NHMUK014331353; • 1♀ Hong Kong, China, Tai Yeung Che, Tai Po, New Territories, 22.447N 114.128E, 65 m, 5 May 2017, leg. M.J. Sterling, specimen no. NHMUK010923242, slide no. NHMUK014331355; • 1♀ Hong Kong, China, Tai Lam Wu, Sai Kung, 22.405N 114.303E, 90 m, 31.05.2018, leg. M.J. Sterling, specimen no. NHMUK010923248, slide no. NHMUK014331354.

##### Remarks.

The type of *Athrypsiastissalva* Meyrick, 1932 was a male from Shanghai, China. The type was designated by Meyrick and, according to the original description, it is in the Caradja collection. Following the death of Prince Aristide Caradja, his collection was deposited with the Muzeul National de Istorie Naturala “Grigore Antipa” in Bucharest, Romania. Popescu-Gorj (1992) produced a catalogue of the Lepidoptera types held in the collections of this museum and there is no reference to that type in this catalogue. The museum also has on its website a list of the Lepidoptera types which it holds and there is no reference to this type on the website. We have corresponded with Dr Mihai Stanescu, the current collection manager for Lepidoptera at the museum, who kindly sent us a copy of the original description of *A.salva* which was annotated by Popescu-Gorj while preparing his Catalogue. The words “Atrypsiastis salva sp. nov.” [sic] are underlined in red; the words “Type in coll. Caradja” are underlined in pencil; and the word “Holotype” has been inserted in the margin in pencil. Popescu-Gorj was therefore clearly aware that this type should have been held at this museum and the absence of any reference to it in his Catalogue indicates that he was not able to locate it. Dr Stanescu has conducted a further search for the specimen but has not found it.

Meyrick’s original description mentions a further example from China which was probably this species, although it contains no further details of the specimen. There are two specimens in the NHMUK from Meyrick’s collection which are labelled *Athrypsiastissalva* (a male and a female), both collected from Hangchow (= Hangzhou), China. The only other white xyloryctid moth from China in the NHMUK collection, acquired from the Meyrick collection, is the type of *Metathrincaintacta* Meyrick, 1938. It can therefore be strongly presumed that one of the ‘*Athrypsiastissalva*’ from the Meyrick collection is the other specimen referred to by Meyrick in his original description.

We have dissected both the male and the female and both have the distinctive *Topiris* genitalia (in the case of the male typical of those of the *salva* group). Also, both the genitalia of the male and the female are identical to the male and female genitalia of recent materials within the study group, collected from Hong Kong, China, for which DNA barcodes have been obtained. We have designated a barcoded male from Hong Kong as the neotype of *Topirissalva* (Meyrick, 1932) and the male specimen from the Meyrick collection as a paraneotype.

The small white forms with no significant markings appear externally to be different to the larger off-white-coloured forms with greyish colouration in the thicker scaling on the dorsum but the genitalia are the same and the sequenced specimens of both forms provided the same haplotype.

In our view Meyrick’s etymology is as follows: *salva* (Meyrick, 1932) — from *salvare* (lat.) to save; *salva* is the present active imperative, e.g., ‘salva me fons pietatis’ from the Latin requiem mass. It is however likely to be a reference to Christ the Saviour, who is traditionally depicted clothed in white. Although this species is slightly variable, in Meyrick’s original description the type specimen was white.

*Athrypsiastissalva* Meyrick, 1932 falls within the *Topiris* clade (Figs 1–3). We therefore combine *A.salva* Meyrick, 1932 as *T.salva* (Meyrick, 1932).

For the reasons set out in the Discussion section, the Chinese pest species on *Camelliaoleifera* (Theaceae) and tea reported as *Linoclostisgonatias* in Han et al. (2007) and Su et al. (2020) is *Topirissalva* (Meyrick) and not *L.gonatias* Meyrick (the latter being known only from the female type specimen).

#### ﻿ Topiris albogrisella

Taxon classificationAnimaliaLepidopteraXyloryctidae

Sterling & Lees sp. nov.

B980BDC8-A9B2-5FFA-811A-BAA0487F9070

https://zoobank.org/B2EB8EF1-6803-4030-9D5A-DAF343D7246A

Figs 14 , 40A, B

##### DNA barcode.

N/A.

##### Type material.

Malaysian Borneo: ***Holotype*** • ♂, Sabah, Gunong Monkobo, 116.56E, 5.48N, Dipterocarp Forest, 7–13.viii.1987, K.R. Tuck leg., fwl 8 mm, specimen no. NHMUK010923006, slide no. NHMUK010316356. ***Paratype*** • ♂, same collection data as holotype, specimen no. NHMUK010923005, slide no. NHMUK010316437.

##### Diagnosis.

The slightly off-white colour of the forewings and brownish grey tinge to the thicker scaling on the dorsum may distinguish this species externally from other *Topiris* but external determination cannot be made with certainty. In the male genitalia, the only other species with a long filament-like distal projection of the aedeagus with similar genitalia is *T.madonna.* This species can be distinguished from *T.madonna* by the emarginate apical margin of the valva and the apex of the uncus which is broad and rounded in this species but narrower and rectangular in *T.madonna*. (Figs 40A, 42A).

##### Description.

**Male** (Fig. 14). Forewing length 8 mm; wingspan 17.5 mm. ***Head***: frons with appressed dirty white scales; vertex with long narrow white scales projecting away from base of antennae, further tufts of white scales projecting sideways from sides of occiput, two tufts of long white scales projecting posteriorly from posterior margin of occiput, overlaying in part a collar of broader white appressed scales projecting posteriorly from anterior margin of prothorax; pilifers short, cylindrical, with tufts of short dark bristles; maxillary palps whitish. Labial palps long (almost 3× diameter of eye); strongly recurved; basal segment with small scale tuft; second segment longer than third, strongly curved with thin appressed matt white scales; third segment slightly curved with thin tightly appressed white scales. Haustellum with basal portion scaled silver white. Antenna ¾ length of forewing, bipectinate; scape with appressed pale ochreous scales; flagellum with short dark pectinations covered in short sensillae for ¾ of length, apical portion filiform; basal part of dorsal surface scaled pale ochreous for a short distance, thereafter dark with scattered silver scaling. ***Thorax***: greyish ochreous with silver iridescence; tegulae short, paler. Foreleg with femur white, tibia and tarsus with dark brown scaling mixed with white; broad tibial epiphysis; mid legs and hind legs white; hind legs with dense white scale tuft. Forewing broad, costa gently curved at base, thereafter almost straight, apex rounded, termen angled inwards, tornus obtusely angled; white with a pale greyish ochreous tinge, unmarked except for a line of brown scales from the base of the costa to ~ 1/5, thicker scaling on dorsum terminating well before tornus, tinged greyish ochreous though indistinct, cilia white. Hindwing as broad as forewing, rounded and broad with a very slightly pointed apex; white. Ventrally, forewing with brown scaling between costa and Sc, veins lined with pale brown scales; hindwing dull white.

**Female.** Unknown.

***Pre-genital abdomen*.** White. Tergal spines on posterior part of T2–T7; sternites and part of T8 weakly sclerotised. Apodemes curved anteriorly, venulae short.

***Male genitalia*** (Fig. 40A, B). Uncus broad, anterior margin of dorsal surface weakly emarginate, broadening sub-apically, apically spatulate, with two small lateral processes on dorsal surface. Gnathos fused medially, lateral arms thin and lightly sclerotised, medial plate small and lightly sclerotised, weakly projecting posteriorly from lateral arms. Tegumen broad and strongly arched, lateral extensions of tegumen longer than width of tegumen band. Vinculum robust, well sclerotised, U shaped, base substantially projecting anteriorly beyond the base of the valvae. Saccus large. Juxta with narrow V-shaped basal plate, anellus lobes broad at base, moderately long. Valva broad throughout, broadening distally, apical margin slightly emarginate, costal ventral membrane with long setae and a small tuft of bristles distally, apex of valva with tuft of short bristles, saccular margin of valva slightly curved, strong ventral sclerite postmedially with projection attaching to base of saccular process. Sacculus almost as broad as valva, longer than broad. Saccular process commencing near costa of valva, very broad, almost square in appearance, without setae or bristles, with a short fat strongly sclerotised hook apically. Aedeagus long, strongly curved, with a long, recurved, filament-like distal projection and a small distal thickening. Bulbus ejaculatorius long with two coils and a long hood.

##### Biology and early stages.

Early stages unknown. Adult found in dipterocarp forest at 975 m in August.

##### Distribution.

Sabah, Malaysia.

##### Etymology.

*albogrisella* — from *albus* (lat.), white; and *griseus* (lat.), grey. The forewings of this species are white with a slight grey tinge. The epithet is an adjective in the nominative singular.

#### ﻿ Topiris lacteella

Taxon classificationAnimaliaLepidopteraXyloryctidae

Sterling & Lees sp. nov.

970319F3-E10F-50F9-813F-8F087AB27BF6

https://zoobank.org/6FC7D7ED-4FAB-4C92-B2DC-7D97FFA22C5A

Figs 15 , 41A, B

##### DNA barcodes.

BIN, BOLD:ADZ8474 (Process IDs METAT196-19, METAT217-19, METAT218-19, METAT234-19).

##### Type material.

Thailand: ***Holotype*** • ♂, W. Thailand, Kanchanburi District, Than Lodh, 400 m, 16.v.1987, M.G. Allen leg., fwl 8.5 mm, specimen no. NHMUK010923157, slide no. NHMUK010316362, Process ID METAT196-19. ***Paratypes*** (3♂): • 1♂ Chiang Mai, Samoeng/Hang Dong Rd, km 15, NW Thailand, 700 m, 05.vi–12.vi.1988, I.J. Kitching leg., specimen no. NHMUK010923141, slide no. NHMUK010316438, Process ID METAT234-19; • 1♂ W. Thailand, Kanchanburi District, 300 m, 24.v.1987, Col M.G. Allen leg., specimen no. NHMUK010923177, slide no. NHMUK010316439, Process ID METAT217-19; • 1♂ W. Thailand, Uthai Thani, Khao Nang Rum, 400 m, 16.vi.1986, Col. M.G. Allen leg., specimen no. NHMUK010923178, slide no. NHMUK010316440, Process ID METAT218-19.

##### Diagnosis.

The largest white member of the *salva* group. In the male genitalia the uncus is indented sub-apically and apically curved, the valva is rounded at the apex, the saccular process is broad, triangular and setose with a bristle brush commencing towards its apex and the costal margin of the valva is strongly projecting inwards at the base (Fig. 41A).

##### Description.

**Male** (Fig. 15). Forewing length 8–8.5 mm; wingspan 17–18 mm. ***Head***: frons with appressed pure white scales; vertex with long white scales projecting away from the base of the antenna and further such scales projecting upwards from occiput, overlaying in part a collar of broader white appressed scales pointing posteriorly from anterior margin of prothorax; pilifers small with small tufts of dark bristles; maxillary palps white. Labial palps long (2½× diameter of eye), strongly recurved; basal segment with small tuft of white scales; second segment longer than third, white; third segment white. Haustellum scaled silver white basally. Antenna > ¾ length of forewing, bipectinate; scape white, flagellum with moderately sized dark pectinations covered with short white sensillae for > 1/2 length; dark throughout but with scattered white scales, apical portion filiform. ***Thorax***: all specimens examined worn, but with shortish lamellate silvery white scales; tegulae silvery white; forelegs with femur whitish, tibia and tarsus brown; moderate tibial epiphysis, mid legs and hind legs white, with thin white tufts of long scales. Forewing broad, costa very slightly rounded at base, thereafter straight, apex gently curved, termen angled slightly inwards, tornus obtusely rounded, white, unmarked except for a line of brown scales from the base of the costa to ~ 1/5. Hindwing as broad as forewing, rounded and broad with a very slightly pointed apex, white, unmarked. Ventrally, surface of forewing with patch of white scales at base of costa, pale brown scaling between costa and Sc on forewing, forewing veins lined with dark cream scales; hindwing white.

**Female.** Unknown.

***Pre-genital abdomen*.** White with white anal tuft. Tergal spines on posterior part of T2–T7, T8 weakly sclerotised in part; sternites almost unsclerotised. Apodemes almost straight, venulae sinuate.

Male genitalia (Fig. 41A, B). Uncus broad, anterior margin of dorsal surface weakly emarginate, apically short and broad, spatulate, indented sub-apically, apical margin curved. Gnathos fused, lateral arms thin and lightly sclerotised, medial plate small and lightly sclerotised, slightly projecting posteriorly from lateral arms. Tegumen band broad and strongly arched, lateral extensions of tegumen substantially longer than width of tegumen band. Vinculum broad, robust, U shaped, base substantially projecting anteriorly beyond base of valvae. Saccus large. Juxta with broad basal plate, anellus lobes short and broad. Valva long and broad, costal margin strongly projecting inwards at base, projection setose, large setose ventral membrane from costa, pre-apical area of valva with fine setae, apex rounded, saccular margin slightly curved, strong ventral sclerite postmedially. Sacculus almost as broad as valva, longer than broad. Saccular process developing from distal part of sacculus, commencing close to costa of valva, broad, triangular, setose throughout with an inwardly curved bristle brush commencing pre-apically and projecting beyond apex of process. Aedeagus long with long, recurved, filament-like projection and small, rounded, distal thickening. Bulbus ejaculatorius long, two coils, broad elongated hood.

##### Biology and early stages.

Early stages unknown. The adult has been found in forest habitats at 300–700 m elevation in May and June.

##### Distribution.

West and northwest Thailand.

##### Etymology.

*lacteella* — from *lacteus* (lat.) milky. This is a reference to the milky white colour of the wings of this species. The epithet is an adjective in the nominative singular.

#### ﻿ Topiris madonna

Taxon classificationAnimaliaLepidopteraXyloryctidae

Sterling & Lees sp. nov.

6F6ECC46-4822-5AFE-9201-C8CFB0D24189

https://zoobank.org/3FD1C3B0-086E-49C6-A676-EAC8D527CA60

Figs 16 , 42A, B

##### DNA barcode.

BIN: N/A. The sequence fragment obtained (PP131485) was too short to qualify for a BIN.

##### Type material.

Peninsular Malaysia: ***Holotype*** • ♂ W. Malaysia, Selangor, Bangi, UKM campus, lowland dipterocarp forest, 70 m, 25–26.vii.1991, G.S. Robinson leg., fwl 6.5 mm, specimen no. NHMUK010922998, slide no. NHMUK010316357, Process ID METAT030-18 (307 bp).

##### Diagnosis.

The only white member of the *salva* group in which the flagellum of the antenna is thickly scaled white for most of its length. In the male genitalia the broad, triangular saccular process with a sclerotised hook at the apex distinguishes this species from its congeners, although the saccular process slightly resembles the saccular process of *T.albogrisella*. For differences in the male genitalia of these two species see Diagnosis for *T.albogrisella* above and compare Fig. 40A and Fig. 42A.

##### Description.

**Male** (Fig. 16). Forewing length 6.5 mm, wingspan 14 mm. ***Head***: frons with appressed white scales with some iridescence; vertex with a tuft of white scales projecting away from the base of the antennae, occiput with some further white scales projecting upwards and posteriorly from the sides, overlaying in part remains of a collar of broad white scales projecting posteriorly from the anterior margin of prothorax; pilifers small with small tufts of bristles; maxillary palps not visible. Labial palps long, > 2.5× diameter of eye, strongly recurved; basal segment with small white scale tuft, second segment longer than third, strongly curved with a moderate covering of appressed scales, some ochreous colouration at sides; third segment almost straight with a thin covering of white appressed scales. Haustellum with white scaling on basal portion. Antenna ¾ length of forewing, bipectinate; scape with appressed white scales; flagellum with short dark pectinations for ¾ of length, pectinations covered in short white sensillae, dorsal surface and sides with thick white scaling for most of length, apical portion filiform. ***Thorax***: covered with white appressed scales; tegulae fairly short, white; femur white, tibia and tarsus brown, moderately large tibial epiphysis, mid legs and hind legs white, hind legs with tuft of white scales. Forewing broad, costa slightly rounded at base, otherwise straight, apex obtusely rounded, termen angled very slightly inwards, tornus obliquely angled, white with silvery iridescence, unmarked except for a line of brown scales from the base of the costa to ~ ¼. Hindwing as broad as forewing, rounded with a very slightly pointed apex, white. Ventrally, forewings with area between costa and Sc pale ochreous, pale ochreous scaling along veins, hindwings white.

**Female.** Unknown.

***Pre-genital abdomen*.** White, white anal tuft.

***Male genitalia*** (Fig. 42A, B). Uncus broad, anterior margin of dorsal surface weakly emarginate, apically spatulate, almost rectangular, apical margin straight. Gnathos fused medially, medial plate small and lightly sclerotised, slightly projecting posteriorly from lateral arms. Tegumen band broad and strongly arched, lateral extensions of tegumen longer than width of tegumen band. Vinculum long, robust, U shaped, well sclerotised, base substantially projecting anteriorly beyond base of valvae. Saccus moderately large. Juxta with U-shaped basal plate, anellus lobes broad, moderately long. Valva long, broad throughout, costal margin distally curved, long setose ventral membrane from costa, apex of valva with tuft of short bristles, saccular margin of valva curved at base, otherwise straight, strong, thin ventral sclerite postmedially. Sacculus longer than broad. Saccular process developing from distal part of sacculus, commencing near costa of valva, broad, triangular, basal margin long, thick, straight, and well sclerotised, remainder of process well sclerotised with a large, strongly sclerotised hook at apex. Aedeagus long with a long, recurved, filament-like distal projection and pronounced distal thickening.

##### Biology and early stages.

Early stages unknown. Adult recorded from lowland dipterocarp forest in July.

##### Distribution.

Selangor, Malaysia.

##### Etymology.

*madonna* (modern lat.) means, among other things, an artistic depiction of the Virgin Mary. The Madonna is often depicted in religious iconography as dressed in white. The epithet is a noun in the nominative singular.

#### ﻿ Topiris meyricki

Taxon classificationAnimaliaLepidopteraXyloryctidae

Sterling & Lees sp. nov.

3344D494-A140-5E52-808E-20046747AE15

https://zoobank.org/B8D1C8C4-9601-4EF5-B791-5F646029A779

Figs 17 , 43A, B , 68

##### DNA barcode.

N/A.

##### Type material.

Thailand: ***Holotype*** • ♂, Siam, W.R.S. Ladell leg., fwl 7.5 mm, specimen no. NHMUK010219685, slide no. NHMUK010316359.

##### Diagnosis.

Indistinguishable externally from other white species of *Topiris*. In the male genitalia, the valva is strongly projecting apically with a smaller, sclerotised, thorn like subapical projection below the apical projection. The saccular process is broad and claviform with a large patch of short bristles medially and a small sclerotised tooth like posterio-medial projection.

##### Description.

**Male** (Fig. 17). Forewing length 7.5 mm, wingspan 16 mm. ***Head***: frons with appressed white scales, some cream scales at sides; vertex with tufts of long white scales laterally pointing upwards and away from base of antennae, further long white scales laterally pointing inwards, overlaying the remains of a collar of broader white scales on anterior margin of prothorax pointing posteriorly; pilifers small with small tufts of bristles; maxillary palps whitish. Labial palps, long (> 2.5× diameter of eye), strongly recurved; basal segment white, second segment strongly curved, longer than third segment, thinly covered in appressed white and ochreous scales; third segment thin and pointed, almost straight, thinly covered in white appressed scales. Haustellum with white scaling at base. Antenna ¾ length of forewing, bipectinate; scape white, flagellum with moderate black pectinations, white scaling on basal part (apical part of both antennae missing). ***Thorax***: cream coloured, tegulae fairly short, white. Foreleg with femur and tibia pale buff, tarsus damaged, mid legs missing, hindleg with remains of tuft of long white scales. Forewing broad, costa slightly rounded at base, thereafter almost straight, apex obtusely rounded, termen angled slightly inwards, tornus obtusely rounded, dullish white, unmarked apart from traces of brown scaling at edge of base of costa to ~ 1/5. Hindwing as broad as forewing, very slightly pointed at apex, otherwise rounded, white, unmarked. On ventral surface forewings with pale brown scaling between costa and Sc and along veins, hindwings white.

**Female.** Unknown.

***Pre-genital abdomen*.** Cream coloured, white anal tuft. Tergal spines on posterior parts of T2–T7, part of T8 and sternites weakly sclerotised. Apodemes almost straight.

***Male genitalia*** (Figs 43A, B, 68). Uncus broad, anterior margin of dorsal surface weakly emarginate, broadening subapically, apically spatulate, dorsal surface with large medial carina and bow shaped plate basally. Gnathos fused medially, lateral arms thin and lightly sclerotised, medial plate small and lightly sclerotised, very slightly projecting posteriorly from lateral arms. Tegumen band broad and strongly curved, lateral extensions of tegumen substantially longer than width of tegumen band. Vinculum long, robust, well sclerotised, U shaped, base substantially projecting anteriorly beyond base of valvae. Saccus large. Juxta with basal plate V-shaped, anellus lobes broad and moderately short. Valva broad and long, costal margin with substantial medial projection at base, also strongly projecting apically with a smaller, sclerotised, thorn like subapical projection below the apical projection, saccular margin of valva curved at base, otherwise straight, thin ventral sclerite postmedially. Sacculus large, longer than broad, a few sparse setae. Saccular process developing from distal part of sacculus, commencing near costa of valva, broad, claviform, small sclerotised posterio-medial tooth, thick brush of short bristles medially. Aedeagus long with long, recurved, filament-like distal projection and a prominent distal thickening with pointed tip (Fig. 68).

##### Biology and early stages.

Unknown.

##### Distribution.

Thailand.

##### Etymology.

*meyricki* — named in honour of the microlepidopterist and systematist Edward Meyrick (1854–1938), who described the majority of the known species of white Oriental xyloryctids. The epithet is a noun in the genitive case.

##### Remarks.

The labels for the specimen contain limited collection data. However, its collector, Major William Richard Simpson Ladell, was the Chief of the Division of Chemistry and Entomology at the Siamese Department of Agricultural Research during the 1930s and it can be assumed, as its identity was determined by Meyrick, that it was collected in the 1920s or 1930s. The specimen was determined by Meyrick as *Topiriscandidella* Walker, but its genitalia (not examined by Meyrick) are very different from that species (Figs 32, 33, 43).

#### ﻿ Topiris sampitella

Taxon classificationAnimaliaLepidopteraXyloryctidae

(Lvovsky, 2014) comb. nov.

9D59D67A-EC04-5D73-9692-DE6CE2DE8CDA

Figs 18 , 48

Metathrinca sampitella Lvovsky, 2014: 196.

##### DNA barcode.

N/A.

##### Type material.

Indonesian Borneo [Kalimantan]: ***Holotype*** • ♂, South Borneo, Sampit, 0–50 m, 20.ii.1950, W. Buyn leg., specimen no. RMNH.INS.1283491, slide no. Gen Prep No. 63, A. Lvovsky det. The holotype is held at NBC.

##### Material examined.

Photographic images of the adult, original description including Alexander Lvovsky’s drawing of the male genitalia. The genitalia slide was not located. The original description does not contain an image of the adult.

##### Remarks.

It is apparent from a low-resolution image of the ventral surface of the adult that, in the forewing, R3 is present, R3, R4, and R5 have a common stalk and M3 and CuA1 are stalked. The data which was apparent from the material examined was included in our analyses, although the genitalia drawing does not show the saccus, vinculum, sacculus or tegumen characters and, importantly, according to the original description the aedeagus is missing. However, the drawing of the gnathos shows a typical *Topiris* gnathos, with a medial plate weakly projecting posteriorly from the lateral arms (Fig. 48) and is not *Athrypsiastis*. The species appears in Figs 1b, 2 within the *Topiris* clade and not within the *candidella* or *albidella* groups. It does not form a clade with *Metathrinca*. It is therefore placed within the *T.salva* group.

#### ﻿ Topiris thunbergella

Taxon classificationAnimaliaLepidopteraXyloryctidae

Sterling & Lees sp. nov.

6B445B7B-5556-5B3A-A175-26B9F00DCA3F

https://zoobank.org/5F607815-1B49-46E2-B912-063591532690

Figs 19 , 44A, B

##### DNA barcode.

N/A.

##### Type material.

Thailand. ***Holotype*** • ♂, Thailand, Chiang Mai, Doi Suthep-Pui National Park, San Kuu, 1540 m, 22.iv–6.v.1994, I.J. Kitching leg., fwl 7 mm, specimen no. NHMUK010219684, slide no. NHMUK010316361. ***Paratypes*** (2♂): • 1♂, same collection data as holotype, specimen no. NHMUK010219692, slide no. NHMUK010316444; • 1♂, Thailand, Khao Yai NP, 1200 m, 17.iv.1987, Col M.G. Allen leg., specimen no. NHMUK010219789, slide no. NHMUK010316876.

##### Diagnosis.

The strong ochreous colour and dark yellow scaling on the vertex distinguishes this species from all other species of *Topiris*. In the male genitalia the long, narrow, saccular process, which has long setae basally and medially and which is bristled apically, is not found elsewhere in *Topiris*. The species is externally similar to an undescribed species of *Ptochoryctis* and an undescribed species close to *Metathrincaceromorpha* (Meyrick, 1923) (see Remarks below), both of which have been found in the same part of Thailand.

##### Description.

**Male** (Fig. 19). Forewing length 7–9 mm, wingspan 15–20 mm. ***Head***: frons thickly covered in appressed dark yellow scales; vertex with thick tuft of long dark yellow scales projecting upwards and away from base of antennae, further dark yellow scales projecting posteriorly from occiput, overlaying a collar of broad, brown, appressed scales projecting posteriorly from anterior margin of prothorax; pilifers small with moderate tufts of bristles; maxillary palps pale ochreous. Labial palps long (> 2.5× diameter of eye), strongly recurved; basal segment with small tuft of light brown scales; second segment longer than third, strongly curved, pale yellow on the inner side and brown mixed with pale yellow on the outer side towards base; third segment slightly curved, thickly covered with pale yellow appressed scales. Haustellum with pale ochreous scales basally. Antenna ¾ length of forewing, bipectinate; scape with appressed dark yellow scales, flagellum with black pectinations for ¾ of length, covered in short white sensillae, dorsal surface of most of flagellum covered with yellow scales, apical portion filiform. ***Thorax***: dark brown anteriorly, ochreous posteriorly, tegulae three coloured, dark brown anteriorly, graduating to paler brown and becoming pale ochreous posteriorly, mid legs and hind legs scaled pale ochreous with thin tufts of pale ochreous scales. Forewing broad, costa almost straight throughout, apex slightly pointed, termen angled slightly inwards, tornus obtusely rounded, uniformly golden ochreous, unmarked except for a row of dark scales at base of costa to < ¼; hindwing as broad as forewing, silvery white; cilia pale ochreous. Ventrally, surface of forewing with brown scaling between costa and Sc and along veins, hindwing pale brown.

**Female.** Unknown.

***Pre-genital abdomen*.** Pale ochreous, anal tuft pale ochreous. Tergal spines on the posterior parts of T2–T7; a few tergal spines on T8; sternites weakly sclerotised. Apodemes long, straight, venulae almost straight.

***Male genitalia*** (Fig. 44A, B). Uncus broad, anterior margin of dorsal surface weakly emarginate, narrowing postmedially, apically spatulate, apical margin curved. Gnathos fused medially, lateral arms thin, medial plate small and lightly sclerotised, slightly projecting posteriorly beyond lateral arms. Tegumen band broad and strongly arched, lateral extensions of tegumen substantially longer than width of tegumen band. Vinculum robust, well sclerotised, U-shaped, base substantially projecting anteriorly beyond base of valvae. Saccus large. Juxta with V-shaped basal plate, anellus lobes broad and fairly short. Valva long, broad throughout, costal margin projecting medially and setose, long setose ventral membrane from costa extending for almost entire length of costal margin, apex obtusely rounded, saccular margin of valva slightly indented medially, long thin ventral sclerite postmedially. Sacculus long, substantially longer than broad, slightly setose. Saccular process developing from distal part of sacculus, commencing near costa of valva, long and narrow, almost straight, thickly setose for > 1/2 length, terminating in curved bristle brush projecting beyond apex of process. Aedeagus long, distal projection of aedeagus sheath slightly angulated, recurved, distal thickening of projection small with a small, sclerotised, pointed tip. Bulbus ejaculatorius long, two coils.

##### Biology and early stages.

Early stages unknown. Adults found from 1200–1540 m elevation in April/May.

##### Distribution.

Northern Thailand.

##### Etymology.

*thunbergella* — named in honour of the climate change activist Greta Thunberg, in recognition of her work in raising consciousness of the potentially catastrophic pressures on the natural environment in places where this species and the other species reviewed in this paper occur. The epithet is a noun in apposition.

##### Remarks.

Three externally similar species of unicolourous, ochreous, xyloryctid moth occur in the northern part of Thailand, this species, an undescribed species of *Ptochoryctis* and a further undescribed species close to *Metathrincaceromorpha*. In the two latter species, R3 is absent in the forewing.

### ﻿The *albidella* group

In these two species from Sulawesi, the adult has a dark brown terminal line in the forewing, the gnathos is not fused medially and has two narrow lateral posterior projections (Figs 45A, 47, 74), the setose ventral membrane from the costa of the valva is confined to the basal half of the valva (Fig. 45A) and the aedeagus, which has a long, recurved, filament-like, distal projection, also has a small sclerite medially (Fig. 70).

#### ﻿ Topiris albidella

Taxon classificationAnimaliaLepidopteraXyloryctidae

Sterling & Lees sp. nov.

F8F38414-BA1E-5CE0-9AC7-70954CBDEA85

https://zoobank.org/21B66305-1719-4596-A403-9D15EB273D57

Figs 20 , 45A, B , 47 , 60A, B , 70

##### DNA barcode.

N/A. All five sequence fragments (METAT082-18, METAT083-18, DEPAL051-20, DEPAL062-20, DEPAL063-20: see Suppl. material 2) were too short to qualify for a BIN (see Remarks).

##### Type material.

Indonesia, Sulawesi: ***Holotype*** • ♂, Indonesia, Sulawesi, Utara, Dumoga-Bone National Park, ‘Edwards’ camp, lowland forest, 664m, October 1985, Project Wallace, fwl 8.5 mm, specimen no. NHMUK010923048, slide no. NHMUK010316354, Process ID METAT082-18 (339 bp). ***Paratypes*** (11♂, 2♀): • 1♂ Indonesia, Sulawesi, Utara, Dumoga-Bone National Park, plot B, ca. 300 m, lowland forest, September 1985, Project Wallace specimen no. NHMUK010923049, slide no. NHMUK010316433, Process ID METAT083-18 (368 bp) ; • 1♂ Indonesia, W. Celebes, G. Rangkoenau, 900ft., Nov. 1936, J.P.A. Kalis leg., specimen no. NHMUK010923209, slide no. NHMUK010316434, Process ID DEPAL051-20 (133 bp); • 1♂; Indonesia, W. Celebes, Koelawi Paloe, 3100ft., Mar. 1937, J.P.A. Kalis leg., specimen no. NHMUK010923220, slide no. NHMUK010316435, Process ID DEPAL062-20 (136 bp); • 1♂ Indonesia, W. Celebes, Koelawi Paloe, 3100 ft., Mar. 1937, J.P.A. Kalis leg., specimen no. NHMUK010923221; slide no. NHMUK010316436, Process ID DEPAL063-20 (244 bp); • 1♂ Indonesia, W. Celebes, Lindoe Paloe, 3700ft., Apr. 1937, J.P.A. Kalis leg., specimen no. NHMUK010219660, slide no. NHMUK010316866; • 1♂ Indonesia, W. Celebes, G. Tompoe, 2700ft., Feb. 1937, J.P.A. Kalis leg., specimen no. NHMUK010219693, slide no. NHMUK010316848; • 1♂ Indonesia, Sulawesi, Utara, Dumoga-Bone National Park, ‘Hog’s Back’ Camp, Lowland Forest, 492 m, Project Wallace, September 1985, specimen no. NHMUK010219699, slide no. NHMUK014331359; • 1♂ W. Celebes, Koelawi Paloe, 3100ft., Mar. 1937, J.P.A. Kalis leg., specimen no. NHMUK010219658, slide no. NHMUK014331360; • 1♂ Indonesia, W. Celebes, Loda, Paloe, 4000 ft., May 1937, J.P.A. Kalis leg., specimen no. NHMUK010219763, slide no. NHMUK014331362; • 1♂ Indonesia, W. Celebes, G. Tompoe, Paloe, W. Celebes, 2700 ft., Jan. 1937, J.P.A. Kalis leg., specimen no. NHMUK010923230, slide no. NHMUK014331363; • 1♂ Indonesia, Sulawesi, Utara, Dumoga-Bone National Park, Lowland Forest, 664 m, October 1985, Project Wallace, specimen no. NHMUK010923148, slide no. NHMUK014331364; • 1♀ Indonesia, Sulawesi, Utara, Dumoga-Bone NP, G. Mogogonipa summit 1008m, 18–20.x.1985, Project Wallace specimen no. NHMUK010219701, slide no. NHMUK010316865; • 1♀ G. Rangkoenau, W. Celebes, 900 ft., Nov. 1936, leg. J.P.A. Kalis, specimen no. NHMUK013698481, slide no. NHMUK014331361.

##### Diagnosis.

This species is similar externally to *T.digiticosta*, but in *T.digiticosta* the apex of the forewing is slightly more angulated and the terminal line terminates prior to the tornus. In the male genitalia the distal margin of the valva of *T.albidella* is almost straight, the apex is pointed posteriorly but not projecting and there is a small process towards the basal margin, whereas in *T.digiticosta* the apical margin is strongly concave, forming two distal lobes, the posterior lobe long and digitate, the anterior lobe smaller and curved.

##### Description.

**Male** (Fig. 20). Forewing length 8–9.5 mm; wingspan 17.5–20 mm. ***Head***: frons white with small area of ochreous scaling laterally; vertex with small tuft of white scales projecting forward at base of antennae with thick tufts of long white lamellate scales projecting sideways and diagonally from sides of the vertex, overlaying in part a collar of broad white scales projecting posteriorly from anterior margin of prothorax; pilifers short with small tuft of dark bristles; maxillary palps white. Labial palps long (3× diameter of eye), strongly recurved, basal segment covered in fine ochreous scales, second segment longer than third, strongly curved, white, third segment almost straight, covered in appressed pale ochreous scales. Haustellum with basal half scaled silver white. Antenna ¾ length of forewing, bipectinate, scape thickly scaled, white at base and on much of dorsal surface, otherwise dark cream, flagellum with pectinations for ¾ of length, apical portion filiform, basal flagellomeres scaled dark cream on dorsal surface, otherwise black. ***Thorax***: white with metallic reflections, tegulae short, white; femur of foreleg pale ochreous; tibia and tarsus dark brown intermixed ochreous; moderate tibial epiphysis; mid legs and hind legs white, hind legs with thick tuft of long white scales with metallic reflections. Forewing broad, costa gently rounded at base, otherwise almost straight, apex rounded, termen almost straight, tornus slightly rounded, hindwing broad and rounded; glossy white, unmarked except for small line of dark brown scales at the base of the costa to ~ 1/5 and a dark brown terminal line commencing just prior to apex and terminating after tornus; dorsal patch sometimes visible. Hindwing as broad as forewing, white with a faint terminal line near 1+1A. Ventrally, forewing with brownish grey scaling between costa and Sc on the forewing, veins lined with brownish scales, terminal line also present though not as distinct as the forewing, hindwing white.

**Female.** Similar to male, slightly larger, forewing length 9.5–11 mm; wingspan 20–24 mm, antenna filiform throughout.

***Pre-genital abdomen*.** White with white anal tuft. Tergal spines on posterior part of T2–T7; weak sclerotisation of part of T8 and sternites. Apodemes slightly curved, venulae slightly sinuate.

***Male genitalia*** (Figs 45A, B, 47). Uncus anteriorly broad, anterior margin of dorsal surface weakly emarginate, lateral edges tapering towards posterior apex. Gnathos not fused medially and with two narrow, strongly sclerotised lateral posterior projections. Tegumen band broad, strongly arched, lateral extensions of tegumen longer than width of tegumen band. Vinculum long, robust, U shaped, base substantially projecting anteriorly beyond base of valvae. Saccus large. Juxta with broad V-shaped base plate, anellus lobes broad, tapering. Valva broad, slightly tapering distally, costal margin almost straight, setose ventral membrane from costa confined to inner half of valva, small ridge along anterior margin, distal margin almost straight, pointed posterio-apically, a small, slightly sclerotised process close to angle of distal and saccular margin, saccular margin curved towards base, otherwise straight, long thin ventral sclerite postmedially, projection attaching to basal part of saccular process. Sacculus as broad as valva, longer than broad, with scattered setae. Saccular process arising from distal part of sacculus, commencing medially in valva, straight and narrow, setose for > 1/2 length with brush of long bristles apically. Aedeagus long with long, recurved, filament-like distal projection and pointed distal thickening, with small sclerite postmedially (Fig. 70). Bulbus ejaculatorius long with two coils and large broad hood.

***Female genitalia*** (Fig. 60A, B). Papillae anales short and broad, apophyses posteriores 1½ × length of apophyses anteriores. S8 with posterior and anterior margins medially recessed. Ostium small and circular. Antrum long, straight and narrow, sclerotised, melanised, and strongly scobinate almost throughout. Ductus bursae long and thin, anteriorly coiled around posterior part of corpus bursae, posteriorly thin and membranous, anteriorly finely scobinate. Corpus bursae large and elongate, without signum.

##### Biology and early stages.

Early stages are unknown. The Dumoga-Bone specimens were taken at light at night. The species has been found in February, March, September, October, November, and December in forest habitat from 300–1000 m elevation.

##### Distribution.

Known from the area around Palu in the late 1930s and from the Dumoga-Bone National Park in the mid-1970s. Both are in Sulawesi, Indonesia.

##### Etymology.

*albidella* — from *albidus* (lat.), white. The adult moth of this species is almost entirely white in colour. The epithet is an adjective in the nominative singular.

##### Additional material examined

(57♂): • 24♂ W. Celebes, G. Rangkoenau, 900 ft., Nov. 1936; • 2♂ W. Celebes, G. Rangkoenau, 1800 ft., Dec. 1936; • 3♂ W. Celebes, G Tompoe, 2700 ft., Jan. 1937; • 8♂ W. Celebes, G Tompoe, 2700 ft., Feb. 1937; • 1♂ W. Celebes, Koelawi Paloe, 3100 ft., Mar. 1937; • 3♂ W. Celebes, Lindoe Paloe, 3700 ft., Apr. 1937, (all J.P.A. Kalis leg); • 2♂ Indonesia, Sulawesi, Utara, Dumoga-Bone National Park, 7/8.ii.1985, 7.9.1985 J.D. Holloway leg.; • 3♂ Indonesia, Sulawesi, Utara, Dumoga-Bone National Park, Project Wallace, March 1985; 11♂ Indonesia, Sulawesi, Utara, Dumoga-Bone National Park, Project Wallace, September 1985. The additional material was not examined in detail and does not form part of the type material.

##### Remarks.

No full COI sequences were obtained from any specimen. Short sequences of 133–368 bp were obtained from five specimens (two failure tracking, three next generation sequences). Specimen nos. NHMUK010923048 and NHMUK010923049 produced sequences of 339 and 368 bp respectively. These specimens were both taken in 1985. Specimen nos. NHMUK010923209, NHMUK010923220 and NHMUK010923221 (taken in 1936 and 1937 respectively) were submitted for next generation sequencing and produced sequences of 133 bp, 136 bp and 244 bp respectively. Sequences for NHMUK010923220 and NHMUK010923221 are almost 3% pairwise divergent from NHMUK010923048 and NHMUK010923049. All these specimens are males. Their external characters are the same. Each specimen has been dissected and their genitalia are indistinguishable. We have included all these specimens within *T.albidella* on the basis of the identity in their morphological characters and the substantially incomplete information from their sequences. It remains possible, however, that *T.albidella* contains a species complex. *Topirisdigiticosta* is externally cryptic with *T.albidella* although its male genitalia are readily distinguishable. We have therefore included all the additional material which appear to be this taxon on external examination, without dissection or sequencing, as *T.albidella*, but not included this material in the type material.

#### ﻿ Topiris digiticosta

Taxon classificationAnimaliaLepidopteraXyloryctidae

Sterling & Lees sp. nov.

428FCF1C-77E7-5629-9B72-7F57400D4E78

https://zoobank.org/D1743AA4-29F1-416F-8182-CAB0BA84C86F

Figs 21 , 46A, B

##### DNA barcodes.

N/A.

##### Type material.

Indonesia. ***Holotype*** • ♂, Indonesia, West Celebes, Paloe, 2700 ft., Feb. 1937, J.P.A. Kalis leg., fwl 10 mm, specimen no. NHMUK010923231, slide no. NHMUK010316355.

##### Diagnosis.

See diagnosis for *T.albidella* above.

##### Description.

**Male** (Fig. 21). Forewing length 10 mm; wingspan 21.5 mm. ***Head*** missing. ***Thorax*** white with some pale ochreous scaling; tegulae missing; femur of foreleg pale ochreous; femur of mid leg pale ochreous, tibia and tarsus white; femur of hind leg with white scale tuft. Forewing broad, costa gently rounded at base, otherwise straight, apex rounded, termen angled slightly inwards, tornus rounded; white and unmarked except for a line of dark brown scales at the base of the costa to 1/5 and a terminal line of dark brown scales commencing just prior to apex and ending just before tornus. Hindwing as broad as forewing, white, unmarked apart from a small brown terminal line around 1A+2A. Ventrally, forewing with brownish grey scaling between costa and Sc on the forewing, veins lined with brownish scales, terminal line also present though not as distinct as the forewing, hindwing white.

**Female.** Unknown.

***Pre-genital abdomen*.** White with white anal tuft. Tergal spines on posterior part of T2–T7; weak sclerotisation of part of T8 and sternites. Venulae long, somewhat sinuate.

***Male genitalia*** (Fig. 46A, B). Uncus anteriorly broad, anterior margin of dorsal surface weakly emarginate, lateral edges tapering towards posterior apex. Gnathos not fused medially and with two narrow, strongly sclerotised lateral posterior projections. Tegumen band broad, strongly arched, lateral extensions of tegumen longer than width of tegumen band. Vinculum long, robust, U shaped, base substantially projecting anteriorly beyond base of valvae. Saccus large. Juxta with a broad V-shaped basal plate, anellus lobes broad, tapering. Valva broad, costal margin slightly concave, setose ventral membrane from costa confined to inner half of valva, with long fine setae and ridge at anterior margin, distal margin strongly concave, forming two distal lobes, posterior lobe long and digitate, anterior lobe smaller and curved, saccular margin of valva slightly curved, long thin ventral sclerite postmedially. Saccular process commencing medially in valva, straight and narrow, well sclerotised, strongly setose for > ½ length with brush of long bristles apically. Aedeagus long, with long, recurved, filament-like distal projection, small ovate distal thickening and small sclerite medially.

##### Biology and early stages.

Early stages unknown. Adult found in February.

##### Distribution.

Sulawesi, Indonesia. Known only from the holotype which was found by J.P.A. Kalis in the same area as some of his specimens of *T.albidella*.

##### Etymology.

*digiticosta* — from *digitus* (lat.), a finger; and costa, the distal margin of a wing or a valva. This species is named after the digitate lobe projecting from the costa of the valva in the male genitalia. The epithet is a noun in apposition.

#### ﻿ Topiris

Taxon classificationAnimaliaLepidopteraXyloryctidae

sp. “RMNH.INS.20000”

C9AF036F-7DC6-5B37-B323-CD0AB2D55234

Fig. 22

##### Note.

**Figures 22–31.**
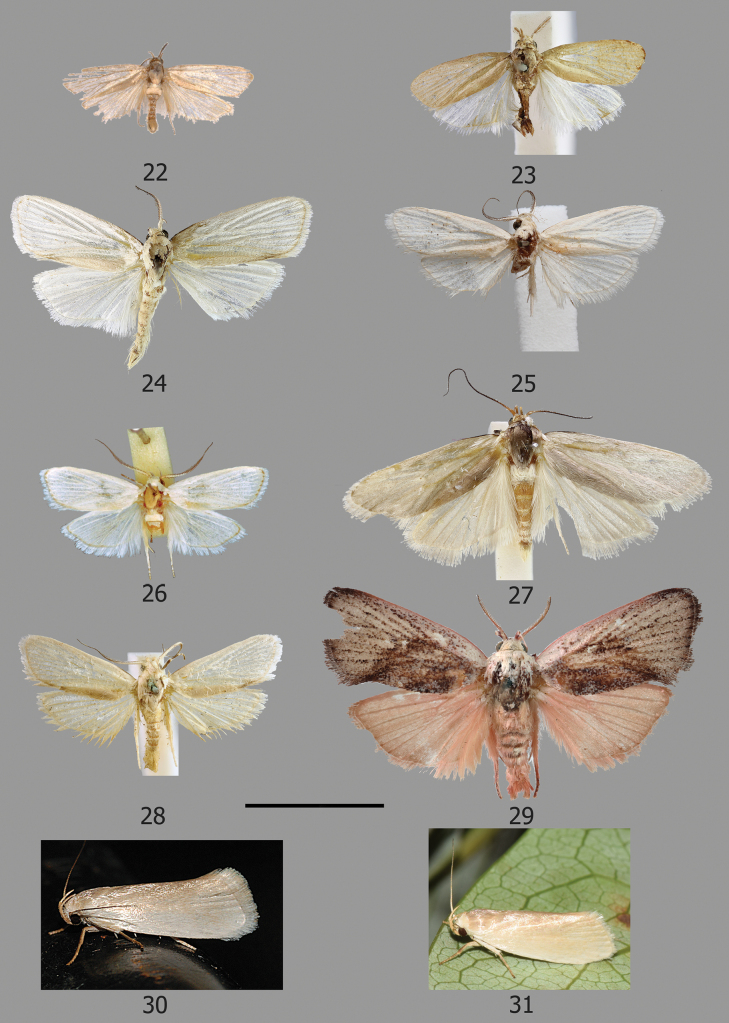
Dorsal images of *Topiris*, *Athrypsiastis* and *Paralecta* spp. **22***Topiris* RMNH.INS.20000 **23***Athrypsiastisphaeoleuca* Meyrick, 1910, holotype ♂ **24***A.cheesmanae* sp. nov., paratype ♂ **25***A.edelweissella* sp. nov., holotype ♂ **26***A.halmaherella* (Lvovsky, 2014), comb. nov., holotype ♂ **27***A.penumbrella* sp. nov., holotype ♂ **28***A.symmetra* Meyrick, 1915, ♂ **29***Paralectarosiflora* (Meyrick, 1930), comb. nov., holotype **30***Topirissalva* (Meyrick, 1932) ♀ **31***T.salva* ♀. Scale bars: 10 mm (**22–29**).

The specimen of which we are aware was found in Kalimantan, Indonesia (Indonesian Borneo), coordinates -1.414, 115.976 at 650 m elevation on 20 November 2005 by Erik van Nieukerken and E. Gasso. The specimen (Museum ID RMNH.INS.20000) has been sequenced (Process ID LEPKM312-10) and belongs to BIN, BOLD:AAL9269. It has a pairwise divergence of 1.37% from *T.cinderella*, whose DNA barcode BIN is BOLD:ADZ9718. The adult is illustrated as Fig. 22. The specimen is a male and has bipectinate antennae. In the forewing, R3, R4, and R5 share a common stalk and R4 and R5 are both post-apical. Although the specimen is very worn, it would appear that it is not iridescent white in colour. The specimen has been dissected with slide number RMNH.INS.20000. An image of the adult was examined by us, but its genitalia slide has not been located. The external characters of this exemplar are consistent with both *Topiris* and *Athrypsiastis* but its barcode places it within *Topiris*. The pairwise distance between this exemplar and *T.cinderella* suggest that it may be a different haplotype of *T.cinderella*. However, we note that BOLD have allocated this exemplar a different BIN from *T.cinderella*.

#### ﻿Genus Athrypsiastis

Taxon classificationAnimaliaLepidopteraXyloryctidae

Meyrick, 1910

F221AB60-43A6-52C7-98A2-7235C7018788

Athrypsiastis Meyrick, 1910: 457.

##### Type species.

*Athrypsiastisphaeoleuca* Meyrick, 1910: 458.

##### Note.

Apart from the limited materials in the NHMUK, the only specimens of *Athrypsiastis* of which we are aware are the holotypes, both by monotypy, of *Athrypsiastiaschionodes* Diakonoff, 1954 and *A.delicata* Diakonoff, 1954. We have examined all the material described or previously identified as *Athrypsiastis* at the NHMUK. We produce here a fuller description of *A.phaeoleuca*. The type of *A.salva* designated by Meyrick is lost, but one of the two specimens from the Meyrick collection must be the other specimen referred to in the original description, and therefore syntypic. The genitalia of both these specimens clearly places them within *Topiris* and the taxon is combined as *Topirissalva* (Meyrick, 1932). *Athrypsiastisrosiflora* is manifestly misplaced to genus (see Figs 22–29 and the species description). Genitalia examination shows that the material identified by Edward Meyrick as *A.symmetra* contained three different species. We describe the morphology of the putative male of *A.symmetra* based on topotypical material from Rossel Island, New Guinea and we describe the other two species, both from Papua New Guinea. We also describe some previously unexamined material from Sulawesi as *A.penumbrella* sp. nov.

##### Diagnosis.

Small to medium sized xyloryctid moths. R3 is present in the forewing and R3, R4, and R5 have a common stalk. M3 and CuA1 are also stalked. The presence of R3 in the forewing distinguishes *Athrypsiastis* from *Linoclostis* and *Metathrinca*. In the male genitalia of *Athrypsiastis*, the medial plate of the gnathos strongly projects posteriorly from the lateral arms, is strongly sclerotised and in most species the projection is posteriorly scobinate. The saccular process is strongly curved towards the saccular margin. The aedeagus sheath is short, does not have a recurved, filament-like, distal projection and one or more cornuti are generally present. These characters distinguish *Athrypsiastis* from *Topiris*.

##### Description.

**Adult. *Head***: Ocelli absent. Frons with appressed scales. Vertex with appressed scales medially, tufts of longer scales laterally and on posterior part of occiput, overlaying collar of long scales pointing posteriorly. Maxillary palps very short. Pilifers short and bristled. Labial palps long to very long (> 2.5–3.5× diameter of eye), strongly recurved. Haustellum scaled basally. Antenna 2/3 to ¾ length of forewing, scape thickly scaled, no pecten, pedicel short, male usually with dark pectinations, reducing at ¾ with apical portion filiform, but occasionally filiform throughout. ***Thorax***: Thorax with appressed lamellate scales. Tegulae short. Foreleg with tibial epiphysis. Tibial spurs 0–2–4. Hindlegs with tuft of long scales. Frenulum of male a single bristle from base of hindwing coupling with retinaculum under a scaled flap towards the base of Sc on the forewing. Forewing venation: R1 from ~ ½ discal cell, R3 present, R3, R4 and R5 with a common stalk, R3 pre-apical or apical, R4 and R5 stalked, R4 usually post-apical, R5 post-apical. M1, M2 and M3 parallel and evenly spaced. M3 and CuA1 stalked. CuP present. Hindwing venation: Sc and Rs widely spaced, M3 and CuA1 connate or stalked (Fig. 63). Forewings broad, hindwings at least as broad as forewings. Dorsal surface of forewings unicolourous except for a small line of dark brown scales at the edge of the base of the costa and, in some species, a faint darker terminal line. In some species there is also a patch of thicker scaling on the dorsum which appears greyish or brownish. Forewing cilia without lines. The pre-genital abdomen is concolourous with forewings with patches of short thick orange-brown tergal spines pointing posteriorly on posterior part of T2–T7 and occasionally a small patch on T8 (Fig. 67).

***Male genitalia*.** Uncus broad anteriorly with anterior margin of dorsal surface usually weakly emarginate or straight, occasionally strongly emarginate, generally tapering strongly towards posterior apex, sometimes posteriorly spatulate or bifid. Gnathos fused medially with large, strongly sclerotised medial plate with single medial projection, strongly projecting posteriorly (Fig. 76). Band of tegumen of variable width, lateral extensions of tegumen generally long, occasionally short. Vinculum U or V shaped, generally projecting substantially beyond base of valvae. Valva large and broad with setose ventral membrane from costa, sometimes with ridge at anterior margin. Sacculus large, longer than broad. Saccular process developing from distal part of sacculus, folded ventrad, large and distinctive, basally curved strongly towards saccular margin, setose and strongly bristled. Aedeagus short and simple, generally with small cornutus/i.

***Female genitalia*.** Unknown.

##### Biology and early stages.

*Athrypsiastissymmetra* is reported from Cinnamon *Cinnamonumverum* J. Presl (= *zeylanicum* Blume) (Lauraceae), Rambutan *Nepheliumlappaceum* L. (Sapindaceae) and Cocoa *Theobromacacao* L. (Sterculiaceae) (Yunus and Ho 1980; Robinson et al. 2001: 70).

##### Distribution.

The genus is known from Papua New Guinea, and Sulawesi and Halmahera Island, Indonesia.

#### ﻿ Athrypsiastis phaeoleuca

Taxon classificationAnimaliaLepidopteraXyloryctidae

Meyrick, 1910

17653170-638E-5DE4-836C-36C4ED07CCE2

Figs 23 , 49A, B , 67 , 71 , 76

Athrypsiastis phaeoleuca Meyrick, 1910: 458.

##### DNA barcodes.

N/A.

##### Type material.

West Papua, Indonesia: ***Holotype*** • ♂, Humboldt Bay, New Guinea, D. 10.93., specimen no. NHMUK010219697, slide no. NHMUK010316398.

##### Diagnosis.

The contrasting pale ochreous forewings and white hindwings distinguish this species from other *Athrypsiastis.* In the male genitalia the uncus is long, rectangular, and apically spatulate and the medial plate of the gnathos strongly projects posteriorly from the lateral arms and is very broad and strongly scobinate (Fig. 76).

##### Description.

**Male** (Fig. 23). Forewing length 7.5 mm, wingspan 17 mm. ***Head***: ocelli absent, frons with dull reddish brown appressed scales; vertex with cream appressed scales, two tufts of long cream scales laterally on vertex, two tufts of long cream scales on posterior part of occiput, pointing posteriorly, overlaying collar of long cream scales, also pointing posteriorly; pilifers moderately broad with a tuft of short bristles; maxillary palps ochreous. Labial palps long (>2.5× diameter of eye), strongly recurved, projecting strongly away from head; basal segment pale ochreous; second segment same length as third, long and strongly curved, evenly scaled pale brown; third segment long, slightly curved, pale brown appressed scales. Haustellum scaled ochreous brown at base. Antennae (both broken) bipectinate; scape pale ochreous, without pecten, dorsal surface of base of flagellum scaled pale ochreous, pectinations brown, covered in short white sensillae. ***Thorax***: pale reddish ochreous, tegulae missing; foreleg brown, small tibial epiphysis, other legs missing. Forewing broad, costa very gently arched at base, thereafter slightly curved, apex rounded, termen angled slightly inwards, tornus very obtusely rounded, forewing yellowish ochreous with a dark pinkish tinge to the naked eye, some dark scaling at edge of base of costa, otherwise unmarked, remains of cilia white. Hindwing as broad as forewing, apex rounded, shining white, unmarked (the asymmetric colouration (see Fig. 23) is staining), cilia white.

**Female.** Unknown.

***Pre-genital abdomen*.** Ochreous, anal tuft pale ochreous. Patches of tergal spines on posterior parts of T2–T7; T8 weakly sclerotised; sternites unsclerotised. Apodemes straight; venulae slightly sinuate.

***Male genitalia*** (Figs 49A, B, 71, 76). Uncus anteriorly broad, anterior margin of dorsal surface very weakly emarginate, long, rectangular, apically spatulate, slightly rounded at apex. Gnathos fused medially, lateral arms long and broad, medial plate very broad, strongly sclerotised and scobinate, strongly projecting posteriorly from lateral arms, pointed apically. Tegumen band elongate, lateral extensions of tegumen longer than width of tegumen band. Vinculum broad, robust, U shaped, base substantially projecting anteriorly beyond base of valvae. Saccus large. Juxta with broad basal plate, anellus lobes broad, moderately short. Valva moderately short, broad, costal margin straight medially, obtusely angled post-medially, setose ventral membrane from costa narrow and without setae basally, broadening and with robust setae medially, extending post-medially, small ridge at anterio-medial edge of membrane, saccular margin of valva almost straight, distal and saccular margins very broadly angled, apex broadly pointed. Sacculus longer than broad. Saccular process developing from distal part of sacculus, commencing above middle of valva, base broad, strongly curved towards saccular margin, curved through > 90° distally, basal half with fine setae, distal half with long thin brush of short appressed bristles, bristles slightly extending beyond apex of process. Aedeagus short, slightly broadened proximally, small ridge-like cornutus in vesica (Fig. 71). Bulbus ejaculatorius narrow, longer than aedeagus, head broad, elongate.

##### Biology and early stages.

Early stages unknown. Adult has been found in October.

##### Distribution.

New Guinea.

#### ﻿ Athrypsiastis cheesmanae

Taxon classificationAnimaliaLepidopteraXyloryctidae

Sterling & Lees sp. nov.

36C2048C-D31E-566B-8399-BCEE29EF6580

https://zoobank.org/6035B44A-F927-4FEC-9249-941EB1CAB458

Figs 24 , 50A, B , 63

##### DNA barcode.

BIN: N/A. The sequence fragment obtained (Process ID DEPAL048-20, Accession PP131476) is too short to have been allocated a BIN.

##### Type material.

West Papua, Indonesia: ***Holotype*** • ♂, Dutch New Guinea, Cyclops Mts., Sabron, 2000 ft., vii.1936, L.E. Cheesman leg., fwl 10.5 mm, specimen no. NHMUK010923206, slide no. NHMUK010316428, Process ID DEPAL048-20 (325 bp). ***Paratypes***: • 7♂, all with collection data same as holotype, specimen no. NHMUK013700129, slide no. NHMUK010316840; specimen no. NHMUK013700128, slide no. NHMUK010316841; specimen no. NHMUK010923085, slide no. NHMUK010316508; specimen no. NHMUK013700095, slide no. NHMUK010316413; specimen nos. NHMUK013700096; NHMUK013700097; NHMUK013699624.

##### Diagnosis.

The adult is indistinguishable from other white species of *Athrypsiastis*. In the male genitalia, the broad, apically pointed, valva is somewhat similar to *A.delicata* Diakonoff but the costal ventral membrane in *A.cheesmanae* is strongly sinuate, the hairs on the costal ventral membrane are longer and denser and the saccular process is more acutely curved towards the saccular margin than in *A.delicata*.

##### Description.

**Male** (Fig. 24). Forewing length 10–11 mm, wingspan 22–23.5 mm. ***Head***: ocelli absent, frons with cream appressed scales; vertex with appressed iridescent white scales, tufts of longer cream scales laterally on vertex, two tufts of long cream scales on posterior part of occiput pointing inwards and posteriorly, overlaying thick collar of broad flat cream scales on anterior margin of prothorax, pointing posteriorly; pilifers with dense brush of short bristles; maxillary palps cream. Labial palps long (> 2.5× diameter of eye), strongly recurved; basal segment small, white; second segment strongly curved, significantly longer than third segment, white with ochreous scaling towards base; third segment long, white appressed scales. Haustellum with silver white scaling on basal portion. Antenna bipectinate, scape white, dorsal surface of flagellum thickly scaled white over entire pectinated portion, remainder of flagellum and pectinations long, brown, covered in short white sensillae. ***Thorax***: cream, tegulae short, cream; foreleg white, large thick tibial epiphysis, mid legs missing on all specimens, hind leg white with thin tuft of long white scales. Forewing broad, costa gently arched at base, thereafter almost straight, apex obtusely rounded, termen slightly angled inwards, tornus obtusely rounded; apex of hindwing rounded, cream white, unmarked except thin line of dark brown scales from costa to 1/5, sometimes a thin patch of dark brown scaling on dorsum and a faint brown terminal line from apex to tornus. Hindwing as broad as forewing, rounded, shining white, unmarked. Ventrally, forewings and veins pale ochreous; hindwings white, unmarked.

**Female**. Unknown.

***Pre-genital abdomen*.** Cream coloured, anal tuft cream coloured. Patches of tergal spines on posterior parts of T2–T7; T8 and sternites weakly sclerotised. Apodemes straight; venulae slightly curved.

***Male genitalia*** (Fig. 50A, B). Uncus anteriorly broad, anterior margin of dorsal surface very weakly emarginate, lateral edges tapering towards posterior apex. Gnathos fused medially, lateral arms broad, medial plate strongly sclerotised and slightly scobinate, strongly projecting posteriorly from lateral arms. Tegumen band strongly arched, elongate, lateral extensions of tegumen slightly longer than width of tegumen band. Vinculum robust, moderately long, very broad at base, U shaped, base substantially projecting anteriorly beyond base of valvae. Saccus moderately large. Juxta with broad U-shaped basal plate, anellus lobes broad and moderately short. Valva long and broad, costal margin sinuate, setose ventral membrane from costa triangular, extending post-medially, covered in dense dark setae, saccular margin of valva slightly convex, narrow ventral sclerite postmedially, distal and saccular margins broadly angled at almost 90°, apex broadly pointed. Sacculus longer than broad. Saccular process developing from distal part of sacculus, commencing medially in valva, long, broad, strongly sclerotised, base broad, strong, short curve basally towards saccular margin, distally curved through > 90°, short bristles on greater part of saccular process, forming a dense thin brush apically, bristles projecting beyond apex of process. Aedeagus short, uniform width throughout, slightly curved, a small ridge-like cornutus posteriorly in the vesica. Bulbus ejaculatorius long, uncoiled, head broad and elongate.

##### Biology and early stages.

Early stages unknown. Adults found in June and July.

##### Distribution.

Dutch New Guinea.

##### Etymology.

*cheesmanae* — named in honour of Lucy Evelyn (Evelyn) Cheesman OBE (1882–1969), a pioneering woman entomologist who made several solo trips to Papua New Guinea in the 1920s and 1930s. This species was collected during her collecting trip to the Cyclops Mountains in Dutch New Guinea in 1936. The epithet is a noun in the genitive case.

##### Remarks.

This is one of three species of *Athrypsiastis* (two in his own collection and this species, deposited in the NHMUK collection by Evelyn Cheesman), determined by Edward Meyrick as *Athrypsiastissymmetra*, but without examination of the genitalia.

#### ﻿ Athrypsiastis edelweissella

Taxon classificationAnimaliaLepidopteraXyloryctidae

Sterling & Lees sp. nov.

5DE22A3C-14D3-591A-82BE-D8AA0EF56DF9

https://zoobank.org/D774598A-6CB0-4073-87EF-F9894C152853

Figs 25 , 51A, B

##### DNA barcode.

N/A.

##### Type material.

West Papua, Indonesia: ***Holotype*** • ♂, Dutch New Guinea, Satakwa [Setakwa] River, M. 3000ft., .7.10., fwl 9 mm, specimen no. NHMUK010219687, slide no. NHMUK010316429.

##### Diagnosis.

The adult is indistinguishable from other white species of *Athrypsiastis*. In the male genitalia the uncus is bifid with shallow medial emargination and the medial plate of the gnathos is very large and strongly sclerotised and projects posteriorly very strongly. The saccular process is very long and thin, substantially projecting beyond the apex of the valva.

##### Description.

**Male** (Fig. 25). Forewing length 9 mm, wingspan 20 mm. ***Head***: ocelli absent; frons with silver white appressed scales; vertex with silver white appressed scales, two tufts of long cream scales laterally on vertex, two tufts of long cream scales on posterior part of occiput pointing inwards and posteriorly, overlaying collar of broad flat silver white scales on anterior margin of prothorax, pointing posteriorly; pilifers not visible; maxillary palps white. Labial palps long (3× diameter of eye), strongly recurved, silver white, closely appressed to head; long, basal segment with small tuft; second segment strongly curved, thinly scaled, slightly longer than third segment; third segment long with white appressed scales. Haustellum with basal portion well scaled silver white. Antenna 2/3 length of forewing, bipectinate, scape white, flagellum with dorsal surface (except filiform portion) scaled silver white, filiform portion black, pectinations long, black, covered in short white sensillae, pectinations reducing at ¾, apical portion filiform. ***Thorax***: with remains of white scaling, remains of tegulae white; foreleg with femur and tibia white, long thin tibial epiphysis, tarsus brown ringed with white; mid legs missing, hind leg silver white with tuft of long silver white scales. Forewing broad, costa gently arched at base, thereafter straight, apex obtusely rounded, termen slightly angled inwards, tornus obtusely rounded, silver white, unmarked apart from small line of brown scales from base of costa of forewing to 1/5 and a faint ochreous tinge towards dorsum of forewing. Hindwing as broad as forewing, rounded, silver white, unmarked. Ventrally, costal area of forewing towards base and veins pale brown, otherwise forewing and hindwing white.

**Female.** Unknown.

***Pre-genital abdomen*.** White, anal tuft white. Patches of tergal spines on posterior parts of T2–T7; T8 and sternites unsclerotised. Apodemes curved; venulae sinuate.

***Male genitalia*** (Fig. 51A, B). The right valva is missing from the specimen but the genitalia are otherwise in good condition. Uncus long with anterior margin of dorsal surface almost straight, apically bifid with shallow medial emargination. Gnathos fused medially, lateral arms large, strongly sclerotised, medial plate very strongly sclerotised, thick and broad, strongly projecting posteriorly from lateral arms, apically pointed and scobinate, gnathos projecting posteriorly further than uncus. Tegumen band deeply arched, moderately narrow, lateral extensions of tegumen short. Vinculum short, broad, robust, base slightly projecting anteriorly beyond base of valvae. Saccus short. Anellus lobes short and broad. Valva long and broad, substantially broadening postmedially, costal margin straight basally, curved distally, large semicircular setose ventral membrane from costa with long fine setae and large ridge at anterior margin, saccular margin of valva straight, small weak ventral sclerite postmedially, distal and saccular margins broadly angled, distal margin angled outwards, apex broadly rounded. Sacculus longer than broad. Saccular process developing from distal part of sacculus, commencing above middle of valva, strongly curved towards saccular margin, very long, almost uniform width, projecting substantially beyond apex of valva, robust setae commencing submedially, double line of short appressed bristles commencing medially, continuing slightly beyond apex of process. Aedeagus narrow throughout, curved, slightly broader posteriorly with some sclerotisation. Bulbus ejaculatorius long, coiled, head large and broad.

##### Biology and early stages.

Early stages unknown. Adult found in July.

##### Distribution.

Dutch New Guinea.

##### Etymology.

*edelweissella* — this all-white species is named after the iconic white alpine flower *Leontopodium nivale* (Ten.) A. Huet ex Hand. (Asteraceae), the common name of which is Edelweiss. The epithet is a noun in apposition.

#### ﻿ Athrypsiastis halmaherella

Taxon classificationAnimaliaLepidopteraXyloryctidae

(Lvovsky, 2014) comb. nov.

9FB30488-ACD2-566D-8EEA-52D9D9235FD3

Figs 26 , 54

Metathrinca halmaherella Lvovsky, 2014: 196.

##### DNA barcode.

N/A.

##### Type material.

Indonesia: ***Holotype*** • ♂, specimen no. RMNH. INS. 1283483, slide no. Gen Prep. No. 62, A. Lvovsky det., Halmahera Island, Goa-Plains, 50–100 m, 9–12.ix.1951. The type is held at NBC.

##### Diagnosis.

The saccular process of this species is scythe-shaped, strongly curving towards the saccular margin. The saccular process of *A.phaeoleuca* is slightly similar in shape but is broader. Also, the uncus of *A.phaeoleuca* is elongate and apically spatulate whereas in this species the lateral edges of the uncus taper towards the posterior apex.

##### Material examined.

We have examined a digitised image of the holotype. The genitalia preparation of the type has not been located but we have examined the original drawing by Alexandr Lvovsky.

##### Remarks.

The data available from the material examined, though somewhat limited, shows that this species does not form a clade with *Metathrinca*. In this species, R3 is present in the forewing, R3, R4, and R5 have a common stalk and M3 and CuA1 are stalked. In the male genitalia, the lateral arms of the gnathos are broad and the medial plate of the gnathos appears to be strongly sclerotised and strongly scobinate and it strongly projects posteriorly. The saccular process is strongly curved both towards the saccular margin and distally. This species has the characters of the other species placed in *Athrypsiastis*. We therefore transfer this species to *Athrypsiastis*.

#### ﻿ Athrypsiastis penumbrella

Taxon classificationAnimaliaLepidopteraXyloryctidae

Sterling & Lees sp. nov.

2C57081B-D4BB-510C-B7F7-3E8EF00E8186

https://zoobank.org/88772AF2-DAB9-4AC3-8123-068290C44A2E

Figs 27 , 52A, B

##### DNA barcode.

BIN, BOLD:ADR3985 (Process ID METAT093-18).

##### Type material.

Indonesia: ***Holotype*** • ♂, Sulawesi, Utara, Dumoga-Bone NP, lower montane forest, 1140 m, March 1985, Project Wallace specimen no. NHMUK010923059, slide no. NHMUK010316430; Process ID METAT093-18; ***Paratypes*** (5♂) • 1♂, same collection data as holotype, specimen no. NHMUK010219686, slide no. NHMUK010316842; • 1♂, same collection data as holotype, specimen no. NHMUK010922452, slide no. NHMUK010316843; • 2♂, same collection data as holotype, specimen nos. NHMUK013700008, NHMUK013700009; • 1♂, October 1985, otherwise same collection data as holotype, specimen no. NHMUK013700010.

##### Diagnosis.

The forewing of this species is greyish white as opposed to the pure white of the other white species of *Athrypsiastis*. The antennae of the male are filiform. In the male genitalia, the anterior margin of the dorsal surface of the uncus is strongly emarginate (Fig. 52A) and the vesica has a small patch of fine spines posteriorly (Fig. 52B).

##### Description.

**Male** (Fig. 27). Forewing length 12.5 mm, wingspan 27 mm. ***Head***: ocelli absent; frons with silver grey appressed scales; vertex with silver grey appressed scales, tufts of long yellowish white scales laterally on vertex, tufts of scales on posterior part of occiput, overlaying collar of broad, flat, silver-grey scales on anterior margin of prothorax pointing posteriorly; pilifers short, cylindrical, with a few bristles; maxillary palps white. Labial palps very long (> 3.5× diameter of eye), strongly recurved, projecting away from head; basal segment with tuft of white scales; second segment long, curved, substantially longer than third, silver grey; third segment long, slightly curved, with silver grey appressed scales. Haustellum scaled basally. Antenna ¾ length of forewing, filiform throughout, brown with some silver scaling, scape without pecten, pedicel short, leading edge of flagellum covered in small white sensillae. ***Thorax***: pale grey, tegulae short, pale grey; foreleg with femur white, tibia dark brown with thin epiphysis, tarsus dark brown mixed paler brown, mid and hind legs white, hind legs with thick tuft of long silver white scales. Forewing broad, costa arched at base, thereafter straight, apex broadly rounded, termen significantly angled inwards, tornus very obtusely rounded; wings and cilia pale greyish white, unmarked apart from a small line of dark brown scales from base of costa to 1/5, dorsal area of forewing with denser, slightly darker grey scaling. Hindwing at least as broad as forewing, rounded with apex very slightly projecting, dull white, unmarked. Ventrally, forewings with fuscous scaling in basal area of costa, otherwise tinged yellowish brown, veins yellowish brown; hindwings white.

**Female.** Unknown.

***Pre-genital abdomen*.** Pale grey; anal tuft grey. Large patches of tergal spines on posterior ½ of T2–T7. Smaller patch of tergal spines on T8; sternites weakly sclerotised. Apodemes slightly curved; venulae straight.

***Male genitalia*** (Fig. 52A, B). Uncus anteriorly broad, anterior margin of dorsal surface strongly emarginate, lateral edges tapering towards posterior apex. Gnathos fused medially, lateral arms long and strongly sclerotised, medial plate strongly sclerotised, strongly projecting posteriorly from lateral arms, apex of projection pointed, gnathos projecting further than uncus. Tegumen band rectangular, slightly curved, lateral extensions of tegumen long and broad, longer than width of tegumen band. Vinculum long, robust, V shaped, base substantially projecting anteriorly beyond base of valvae. Saccus large. Juxta with U-shaped basal plate, anellus lobes moderately long, broad for most of length. Valva long and broad, costal margin straight, slightly projecting medially, large setose ventral membrane from costa extending postmedially, long fine setae distally, anterior margin with curved ridge, saccular margin of valva slightly sinuate, triangular ventral sclerite postmedially, attaching apically to base of saccular process, apex of valva broad, rounded. Sacculus longer than broad. Saccular process developing from distal part of sacculus, commencing above middle of valva, base broad, strongly curved towards saccular margin, curved through 90° distally, basal half setose, distal half with large brush of long robust bristles extending beyond apex of process. Aedeagus slightly broadened proximally, slightly curved, small distal projection, patch of small spines in vesica. Bulbus ejaculatorius very long, thin, almost straight.

##### Biology and early stages.

Early stages unknown. Adults found in March and October at 1140 m elevation.

##### Distribution.

Sulawesi, Indonesia.

##### Etymology.

*penumbrella* — from *penumbra* (*modern lat*.), among other things, a partial shadow. This is a white species which has a greyish shadow on the forewings. The epithet is a noun in apposition.

#### ﻿ Athrypsiastias symmetra

Taxon classificationAnimaliaLepidopteraXyloryctidae

Meyrick, 1915

70C57444-733F-5CC1-B42A-E716455B51E1

Figs 28 , 53A, B

Athrypsiastis symmetra Meyrick, 1915: 377.

##### DNA barcode.

N/A.

##### Type material.

Papua New Guinea: ***Holotype*** • ♀, New Guinea, Rossel Island. ASM. .05, fwl 8 mm, specimen no. NHMUK010219753 (external examination only).

##### Diagnosis.

Externally indistinguishable from other white species of *Athrypsiastis*. In the male genitalia, similarly to *A.chionodes*, the saccular process is almost straight after the curved base whereas in the other species of *Athrypsiastis* it is strongly curved, but the valva is postmedially broad and rounded in *A.symmetra* whereas it is elongate and tapering in *A.chionodes*. The anellus lobes are also shorter in *A.symmetra* than in the other members of this genus.

##### Description.

**Male** (Fig. 28). Forewing length 8–9 mm, wingspan 18–20 mm. ***Head***: ocelli absent; frons with intense white appressed scales; vertex with iridescent white appressed scales, tufts of thin white scales of moderate length laterally on vertex, a ruff of long white scales on posterior part of occiput pointing posteriorly, overlaying collar of long broad flat white scales on anterior margin of prothorax, also pointing posteriorly; pilifers cylindrical, with a few bristles; maxillary palps white. Labial palps strongly recurved, long (3× diameter of eye), thin, closely appressed to head; basal segment with small tuft of cream scales; second segment long, strongly curved, longer than third segment, thinly scaled white; third segment long, thin, with white appressed scales. Haustellum with basal portion scaled silver white. Antenna ¾ length of forewing, bipectinate, scape silver white, without pecten, pedicel short, dorsal surface of flagellum scaled silver white throughout, pectinations long, brown, covered in white sensillae, reducing at ¾, apical portion filiform. ***Thorax***: greyish white, tegulae short, greyish white; foreleg with femur white, tibia white with broad epiphysis, tarsus white mixed ochreous, mid legs and hind legs white with small amount of long white scaling. Forewing broad, costa gently arched at base, thereafter straight, apex obtusely rounded, termen slightly angled inwards, tornus obtusely rounded, white, iridescent, unmarked except for a thin line of pale brown scales from base of costa to 1/6 and an indistinct pale brown patch on the dorsum of some specimens, cilia white. Hindwing as broad as forewing, rounded. Ventrally, forewing and veins scaled yellowish ochreous, otherwise forewing and hindwing white.

**Female.** Similar, antenna filiform throughout, forewing length 10 mm, wingspan 21 mm.

***Pre-genital abdomen*.** Cream coloured, anal tuft cream coloured. Patches of tergal spines on posterior parts of T2–T7; T8 and sternites weakly sclerotised. Apodemes curved; venulae slightly sinuate.

***Male genitalia*** (Fig. 53A, B). Uncus anteriorly broad, anterior margin of dorsal surface very weakly emarginate, lateral edges tapering towards posterior apex. Gnathos fused medially, lateral arms broad, medial plate strongly sclerotised, strongly projecting posteriorly from lateral arms, projection rounded at apex. Tegumen band narrow, deeply curved, lateral extensions of tegumen longer than width of tegumen band. Vinculum robust, U shaped, base substantially projecting anteriorly beyond base of valvae. Saccus large. Juxta with broad U-shaped basal plate; anellus lobes short and broad. Valva long and broad, costal margin sinuate, ventral membrane from costa extending to apex of valva, thickly covered in long dark setae, curved ridge at anterior margin, saccular margin of valva almost straight, small, elongate ventral sclerite postmedially, close to ventral margin, apex of valva broadly rounded. Sacculus longer than broad. Saccular process commencing from distal part of sacculus, initially curving towards saccular margin, joining sub-basally to sclerotised flagellum of process, thin membrane from costal margin of valva also attached to flagellum of process sub-basally, flagellum of process curving distally through 45°, thick appressed robust setae on flagellum from base to pre-apical region, thick brush of long, robust, bristles pre-apically to apex of process, extending beyond apex. Aedeagus almost straight with a ridge-like cornutus in the vesica. Bulbus ejaculatorius long, slightly coiled, long broad head.

***Female genitalia*.** Not examined.

##### Biology and early stages.

A reported pest of *Cinnamonumzeylanicum* L. (Lauraceae) (Robinson et al. 2001: 70), *Nepheliumlappaceum* (Sapindaceae) (Yunus and Ho 1980) and *Theobromacacao* including damaging its bark (Papua New Guinea. Department of Agriculture, Stock and Fisheries 1961: 112; Robinson et al. 2001: 70). Adults recorded between December and January.

##### Distribution.

Rossel Island and Upper Setekwa River, Papua New Guinea.

##### Additional material examined.

(3♂) 2♂, Mt. Rossel, 2100 ft., Rossel Island, Dec. 1915–Jan. 1916 (W.F. Eichhorn), specimen no. NHMUK010219659, slide no. NHMUK010316426; specimen no. NHMUK010923081, slide no. NHMUK010316442; • 1♂, Upper Setekwa [Setakwa] R., Snow Mountains, Dutch N.G., 2–3000 ft., Aug. 1910, specimen no. NHMUK010922380, slide no. NHMUK010316443.

##### Remarks.

This species was described by Meyrick from a single female specimen collected by Albert Meek from Rossel Island, New Guinea. There are no males collected by Meek from Rossel Island. However, there are two males from Rossel Island collected by Albert Frederic Eichhorn in 1916. It is hypothesised here that these are the males of the species described by Meyrick from the Rossel Island female as *A.symmetra* Meyrick. The specimen labelled Upper Setekwa River [sic] (specimen no. NHMUK010922380), which was determined by Meyrick as *A.symmetra*, has the same genitalia as the Rossel Island males.

#### ﻿ Paralecta rosiflora

Taxon classificationAnimaliaLepidopteraXyloryctidae

(Meyrick, 1930) comb. nov.

39EF5662-1CCE-5BF4-92AC-15B1ED539DE5

Athrypsiastis rosiflora Meyrick, 1930: 11.

##### Note.

This attractive species is known only from the type specimen, a male, collected by Albert Meek on the Upper Setakwa River in the Snow Mountains, New Guinea on 16 September 1910 (Fig. 29). We have only examined this specimen externally, but it is clear from this that it does not belong in the genus *Athrypsiastis*. In this species the antennae are bipectinate and orange, tinged pink. All the legs are red. The second segment of the labial palps is red and the third segment is white. In the forewing, R3 is separate from R4 and R5, R4 and R5 are stalked, M1 and M2 approximate at the disc and M3, CuA1 and CuA2 are separate. In the hindwing only M3 and CuA1 are stalked. Externally, it resembles a number of species of *Paralecta* Turner, 1898, to which it is here transferred. *Paralecta* consists of more than 15 species and occurs almost entirely in New Guinea (Robinson et al. 1994). Although Turner’s original concept of *Paralecta* has both 3 and 4 (M3 and CuA1) and 6 and 7 (M1 and Rs) stalked in the hindwing and the antennae are described as shortly ciliate, it has been previously noted (Diakonoff 1954: 89) that several genera of the Xyloryctidae, viz. *Cryptophasa*, *Paralecta*, and others, seem to be rather arbitrary; they show considerable variation as to the neuration, the structure of the male antennae and the length of the terminal segment of the labial palpi, which makes the discrimination of the genera very difficult at times. Notwithstanding the differences in this species from Turner’s original description of *Paralecta*, that genus appears to be the closest to this species and the concept of *Paralecta* is expanded accordingly.

## ﻿Discussion

### ﻿Mitochondrial genome of “*Linoclostisgonatias*”

MT547768 analysed by Su et al. 2020 is the complete mitochondrial genome of a species there identified as ‘*Linoclostisgonatias* (Lepidoptera: Xyloryctidae)’. Although we have not had the opportunity to examine the exemplar from which this sequence was obtained, nor any image of it, the divergence of only two nucleotides in the COI-5P region of this exemplar and NHMUK010922992 (BIN, BOLD:ADS0105) and the absence of any evidence that *T.salva* and the true *Linoclostisgonatias* are related shows that MT547768 represents a misidentified *T.salva*. As far as we are aware, the only verified exemplar of *L.gonatias* is its female holotype, from the Khasis Hills in India. The mistake is more widespread in the literature. Robinson et al. (1994: 63) state that *L.gonatias* is one of the few [species of *Linoclostis*] that are common and widespread. However, *L.gonatias* has some distinctive characters (both M1 and R3 are absent in the forewing (Fig. 65) and the ductus bursae is by a substantial margin the longest of any white Oriental/Papuan xyloryctid moth which we have examined. There is no specimen in the NHMUK collection which those authors determined as this species and no other specimen which we have examined which has both these features.

Meyrick established the genus *Linoclostis* Meyrick, 1908 in which he placed three species. In these taxa, both R3 and M1 are absent in the forewing. There are a few other undescribed species which contain both these characters, but the absence of M1 in the forewing is a rare character in the white Oriental/Papuan Xyloryctidae. Apart from forewing venation, other characters which support the separation of *Linoclostis* from *Topiris* include the presence, in *L.gonatias*, of short recurved palps, a dark subterminal and terminal line, two dark lines in the cilia and iridescent silver patches between the subterminal line and the termen and, in the female genitalia, a short antrum, a very long, membranous, ductus bursae and a signum in the corpus bursae (Fig. 61).

We conclude that *Linoclostis* and *Topiris* are separate genera, the only currently known exemplar of *L.gonatias* is the type specimen and that the sequence MT547768 was obtained from a specimen of *T.salva*.

### ﻿The genus *Athrypsiastis*

There is no support for the monophyly of *Athrypsiastis* in our analyses. However, members of the genus exhibit a combination of morphological characters not otherwise found in related groups of xyloryctids. In common with most Australian and New Zealand species of Xyloryctidae, R3 is present in the forewing. This contrasts with the Oriental genera of *Metathrinca* and *Linoclostis* in which R3 is absent in the forewing (Fig. 65). M3 and CuA1 are stalked in the forewing. This is uncommon in Australian and New Zealand species of Xyloryctidae but is almost always present in species currently placed in *Metathrinca* and *Linoclostis* (although M3 and CuA1 are separate in the forewing of the type species of *Metathrinca* (*Ptochoryctisancistrias*). The saccular process is strongly curved towards the saccular margin. Again, this is rare in Australian, New Zealand and Papua New Guinea species of Xyloryctidae but is common in *Metathrinca* and *Linoclostis*. In common with *Topiris*, R3 is present in the forewing and R3, R4 and R5 share a common stalk (Fig. 63). However, in *Topiris*, the medial plate of the gnathos is lightly sclerotised and projecting posteriorly only slightly from the lateral arms (except in the *albidella* group in which the gnathos is not fused medially and there are two strongly projecting lateral projections), whereas, in *Athrypsiastis*, the medial plate of the gnathos is strongly sclerotised and strongly projecting posteriorly from of the lateral arms (Figs 74–76). Additionally, the aedeagus of *Athrypsiastis* species lack the recurved, filament-like distal projection which is present in *Topiris. Athrypsiastis* is known only from east of the Wallace line. Its distribution is from Sulawesi, Indonesia, in the west to Rossel Island, New Guinea, which is one of the most easterly islands in the New Guinea archipelago. *Topiris*, *Metathrinca* and *Linoclostis* are currently known principally from west of the Wallace line, although *T.albidella* and *T.digiticosta* are here described from Sulawesi and there are a significant number of undescribed species within the *Metathrinca*/*Linoclostis* grouping which have been collected in Sulawesi. These remarks are presented for guidance but this paper highlights the fact that the genus *Athrypsiastis* merits a revision which is beyond the scope of this study.

The topology of Fig. 1b (the morphological only tree) recovers *Metathrinca* + *Linoclostis* as a clade within a polytomy with named species of *Athrypsiastis* (minus *A.penumbrella*), whereas Fig. 2 (combined tree on which morphological characters are mapped) includes *A.penumbrella* in the same polytomy. The character mapping suggesting *Metathrinca* + *Linoclostis* share two non-homoplasious characters with *Athrypsiastis* needs to be qualified by this lack of topological support which reflects data deficiency and/or inapplicable characters for the three genera. *Metathrinca* is a complex genus which is badly in need of revision. Data from many more exemplars would be needed to form even a credible hypothesis as to the true relationship between *Metathrinca*, *Linoclostis* and *Athrypsiastis*. A revision of *Metathrinca* and associated genera is in preparation and is intended to be the subject of a future paper.

We expand Meyrick’s concept of *Athrypsiastis* to include taxa in which the antenna is filiform in the male in order to include *A.penumbrella* within this genus. *A.penumbrella* otherwise exhibits Meyrick’s characters for this genus including, in particular, the unusual forewing venation for Xyloryctidae and it also exhibits the characteristic strongly curved saccular process. We consider this a better course than to establish yet another monobasic genus within Xyloryctidae to accommodate this taxon. We also note that Meyrick’s generic description of *Athrypsiastis* states “ocelli present”. We have not found ocelli to be present in *A.phaeoleuca* or any other species of *Athrypsiastis* we have examined.

**Figures 32–39.**
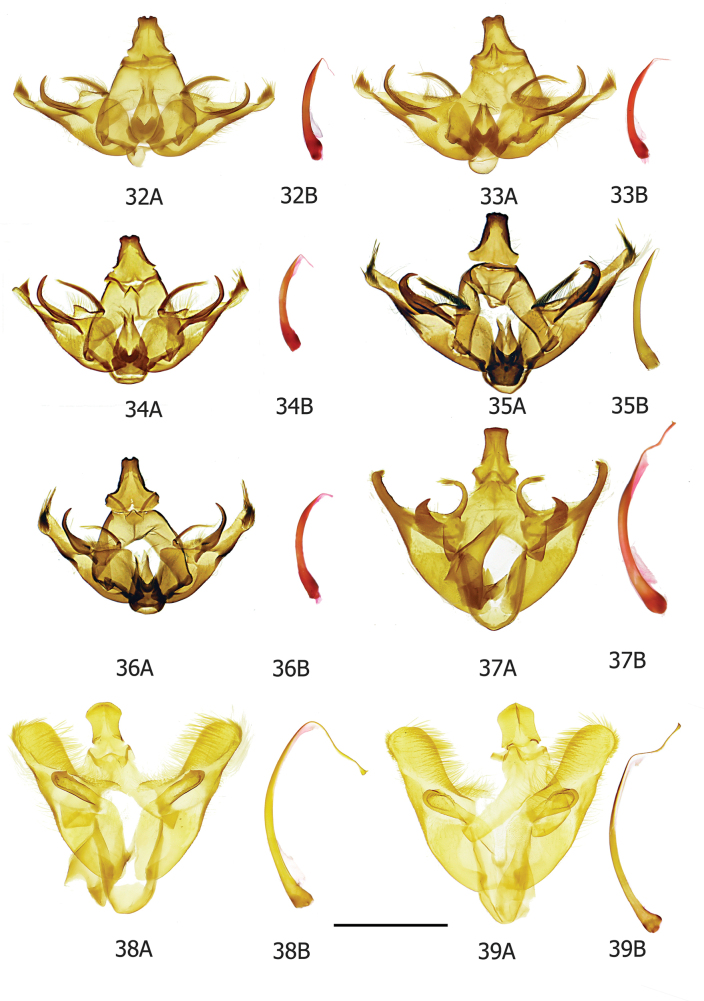
Male genitalia of *Topiris* spp. **32A, B***T.candidella* Walker, 1863, Brunei, slide no. NHMUK010316445 **33A, B***T.candidella*, Sarawak, slide no. NHMUK014331342 **34***T.cinderella* sp. nov. **A** holotype, slide no. NHMUK010316400 **B** paratype, slide no. NHMUK010316448 **35A, B***T.ochrotincta* sp. nov., holotype, slide no. NHMUK010316401 **36A, B***T.schneeweissella* sp. nov., holotype, slide no. NHMUK010316358 **37A, B***T.sericella* sp. nov., holotype, slide no. NHMUK010316873 **38A, B***T.salva* (Meyrick, 1932), comb. nov., slide no. NHMUK010316399 **39A, B***T.salva* (Meyrick, 1932), comb. nov., paraneotype, slide no. NHMUK010316441. Scale bar: 1 mm.

**Figures 40–47.**
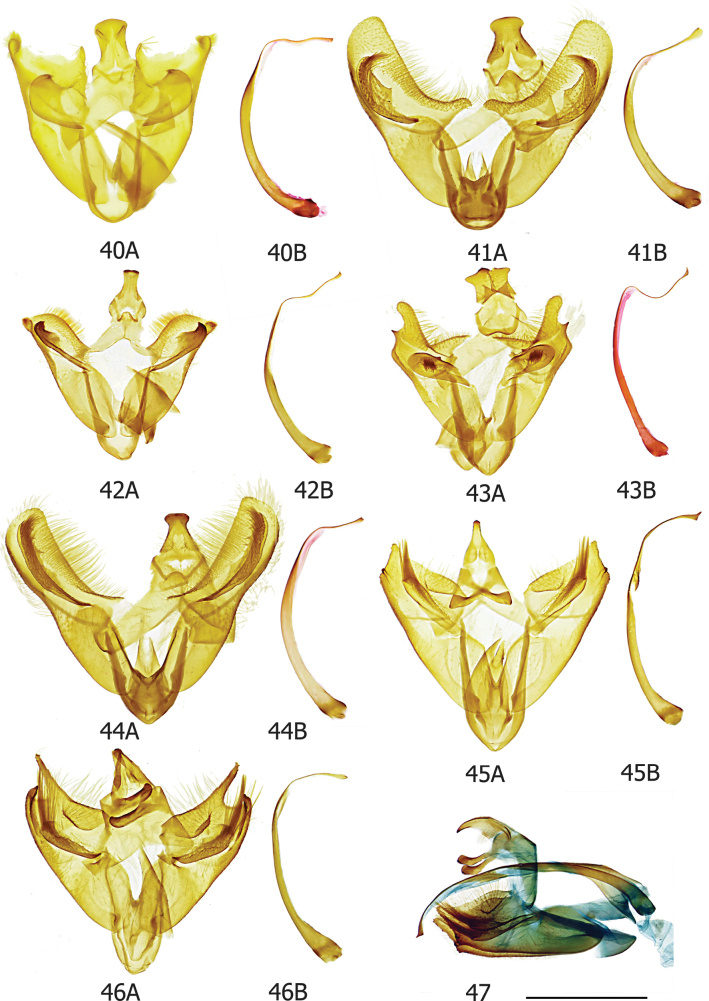
Male genitalia of *Topiris* spp. **40***T.albogrisella* sp. nov. **A** paratype, slide no. NHMUK010316437 **B** holotype, slide no. NHMUK010316356 **41A, B***T.lacteella* sp. nov., holotype, slide no. NHMUK010316362 **42A, B***T.madonna* sp. nov., holotype, slide no. NHMUK010316357 **43A, B***T.meyricki* sp. nov., holotype, slide no. NHMUK010316359 **44A, B***T.thunbergella* sp. nov., holotype, slide no. NHMUK010316361 **45***T.albidella* sp. nov., **A** holotype, slide no. NHMUK010316354 **B** paratype, slide no. NHMUK014331359 **46A, B***T.digiticosta* sp. nov., holotype, slide no. NHMUK010316355 **47***T.albidella* sp. nov., paratype, lateral, slide no. NHMUK014331360. Scale bar: 1 mm.

**Figures 48–56.**
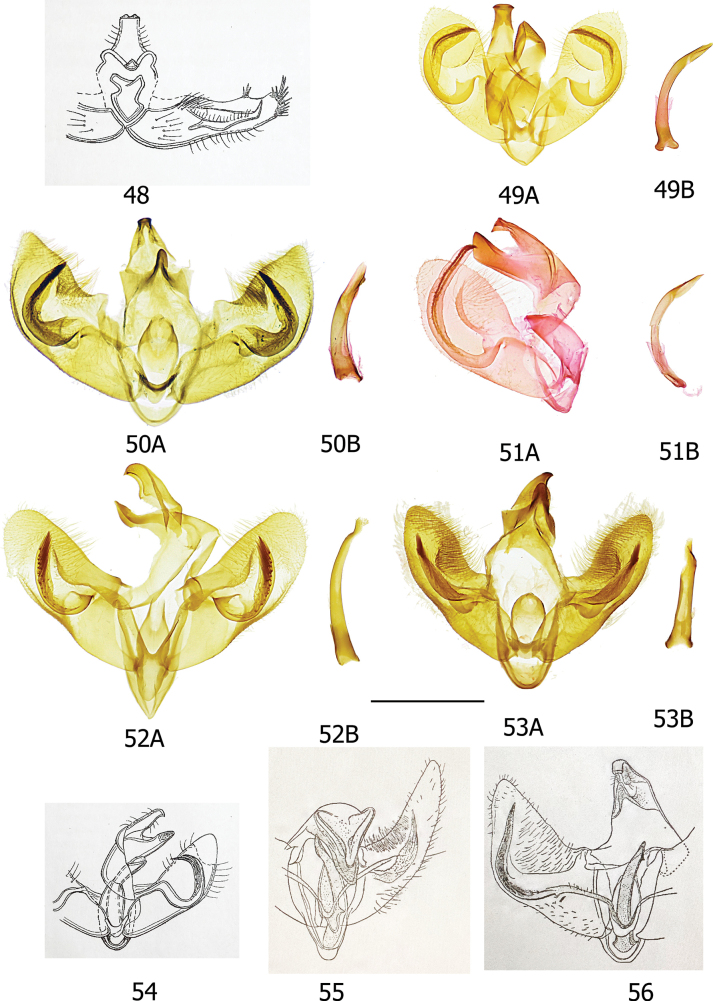
Male genitalia of *Topiris* and *Athrypsiastis* spp. **48***T.sampitella* (Lvovsky, 2014), holotype, Gen. prep. No 63 **49A, B***Athrypsiastisphaeoleuca* Meyrick, 1910, holotype, slide no. NHMUK010316398 **50A, B***A.cheesmanae* sp. nov., holotype, slide no. NHMUK010316428 **51A, B***A.edelweissella* sp. nov., holotype, slide no. NHMUK010316429 **52***A.penumbrella* sp. nov. **A** holotype, slide no. NHMUK010316430 **B** paratype, slide no. NHMUK010316843 **53A, B***A.symmetra* Meyrick, slide no. NHMUK010316443 **54***A.halmaherella* (Lvovsky), holotype, Gen. prep. No 62 **55***A.chionodes* Diakonoff, 1954, holotype **56***A.delicata* Diakonoff, 1954, holotype. Scale bar: 1 mm (**49–53B**).

**Figures 57–65.**
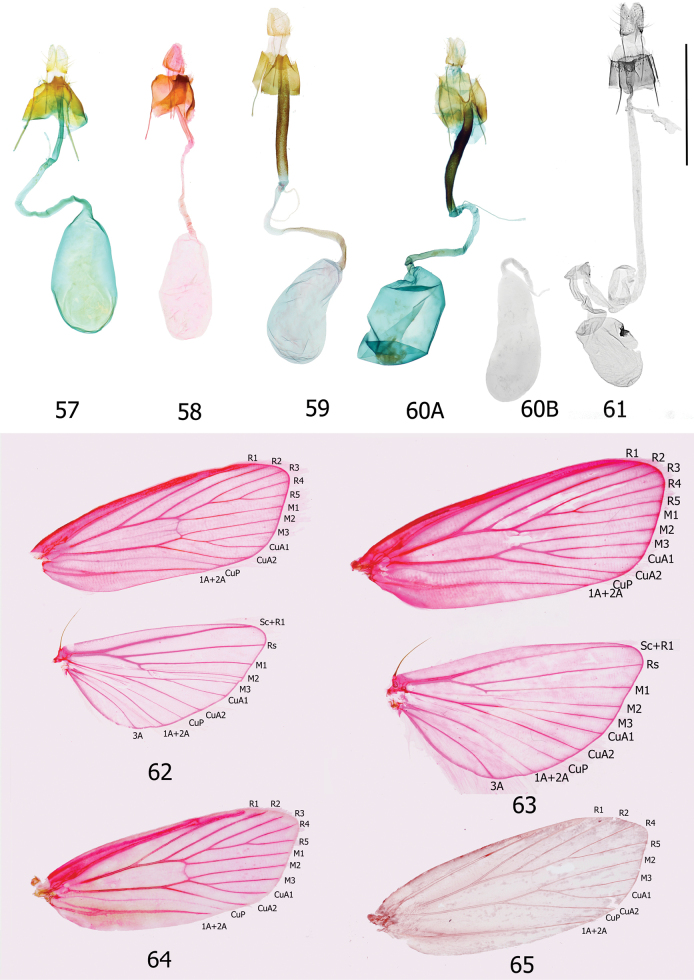
Female genitalia of *Topiris* and *Linoclostis* spp., wing preparations **57***Topiriscandidella* Walker, 1863, slide no. NHMUK014331340 **58***T.schneeweissella* sp. nov., paratype, slide no. NHMUK010316451 **59***T.salva* Meyrick, 1932, comb. nov., slide no. NHMUK014331352 **60A***T.albidella* sp. nov., paratype, slide no. NHMUK010316865 **60B** corpus bursae imaged during preparation **61***Linoclostisgonatias* Meyrick, 1908, type, slide no. JFGC7678 **62***T.candidella*, Brunei, slide no. NHMUK014331889 **63***Athrypsiastischeesmanae* sp. nov., paratype, slide no. NHMUK014331890 **64***T.salva*, forewing venation slide no. NHMUK010316844 **65***L.gonatias*, type, forewing venation, slide no. JFGC7678. Scale bars: 2 mm (**57–61**).

**Figures 66–77.**
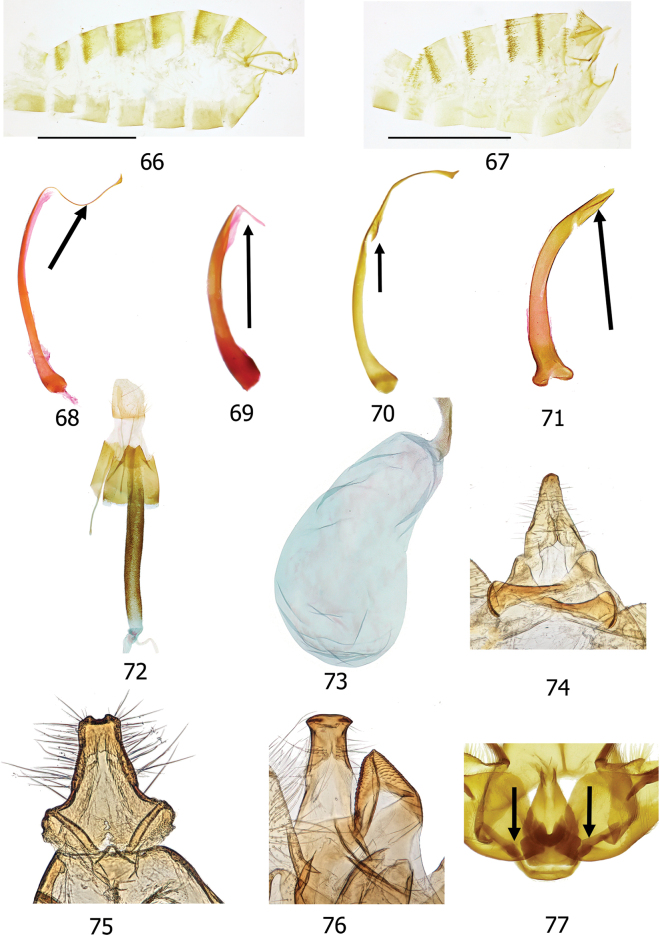
*Topiris* and *Athrypsiastis*, pre-genital abdomens and selected genitalia characters (enlarged) **66***Topiriscandidella* Walker, 1863, Brunei, slide no. NHMUK014331344 **67***Athrypsiastisphaeoleuca* Meyrick, 1910, slide no. NHMUK010316398 **68***T.meyricki* sp. nov., holotype, aedeagus, slide no. NHMUK010316359 **69***T.cinderella* sp. nov., aedeagus, slide no. NHMUK010316448 **70***T.albidella* sp. nov., aedeagus, slide no. NHMUK014331359 **71***A.phaeoleuca* Meyrick, 1910 holotype, aedeagus, slide no. NHMUK010316398 **72***T.salva* (Meyrick, 1932), antrum, slide no. NHMUK014331352 **73***T.salva* (Meyrick, 1932), corpus bursae, slide no. NHMUK014331352 **74***T.albidella* sp. nov., holotype, uncus and gnathos, slide no. NHMUK010316354 **75***T.schneeweissella* sp. nov., holotype, uncus and gnathos, slide no. NHMUK010316358 **76***Athrypsiastisphaeoleuca* Meyrick, 1910, holotype, uncus and gnathos, slide no. NHMUK010316398 **77***T.cinderella* sp. nov., holotype, vinculum, slide no. NHMUK010316400. Scale bars: 2 mm (**66, 67**).

**Figures 78–87.**
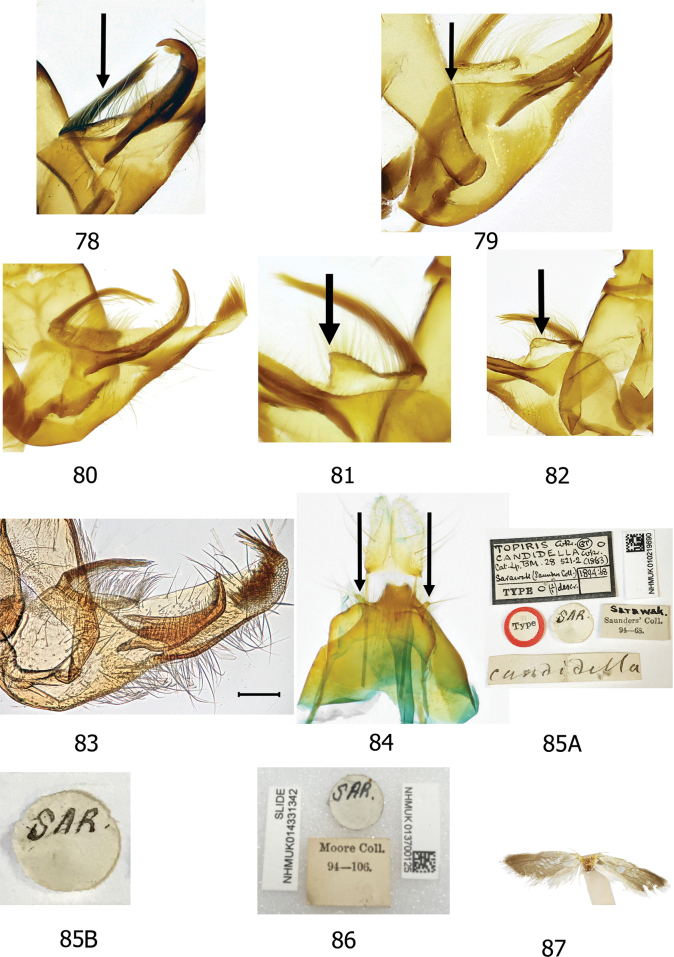
Selected genitalia characters and labels **78***T.ochrotincta* sp. nov., holotype, process of ventral costal membrane, slide no. NHMUK010316401 **79***T.candidella* Walker, 1863, sacculus with strong mesad shoulder, slide no. NHMUK010316360 **80***T.candidella* Walker, 1863, valva, slide no. NHMUK014331342 **81***T.cinderella* sp. nov., holotype, costal ventral membrane of valva distally raised, slide no. NHMUK010316400 **82***T.candidella* Walker, 1863, costal ventral membrane of valva distally flat, slide no. NHMUK010316360 **83***T.schneeweissella* sp. nov., holotype, valva showing setae, slide no. NHMUK010316358 **84***T.candidella* Walker, 1863, processes on S8, slide no. NHMUK014331340 **85A***T.candidella* Walker, 1863 type labels **85B** Wallace’s label (enlarged) **86***T.candidella* Walker, 1863 labels of Wallace specimen, specimen no. NHMUK013700125 **87** hindwings at some time wrongly affixed to type of *T.candidella*. Scale bar: 0.2 mm (**83**).

## ﻿Conclusions

We hope that this revision of *Topiris* and our notes on *Athrypsiastis.* will promote further study of these interesting and, as we show, morphologically and genetically distinctive moths. It will be interesting to explore what further diversity exists within these genera as well as to research how many species can still be found, as only three of the species reviewed in this paper are represented in Natural History Museum, London by specimens collected after the year 2000.

Genome skimming has provided a valuable insight into the genus *Topiris*. Before this technology was available, we had no objective evidence to link Walker’s holotype of *T.candidella* to other species in the genus (including, as it turns out, the available mitogenome of “*Linoclostisgonatias*”), and to distinguish it from *Athrypsiastis*. We would therefore have had no choice but to take Meyrick’s advice that it was better neglected, to establish a new genus for the newly examined taxa, and to consign a Walker type described from a specimen collected by Alfred Russel Wallace to monobasic oblivion. This research is a further testament to the power of museomics in deepening our understanding of taxonomy.

Our study also paves the way to examine more thoroughly the Microlepidoptera collected by Alfred Russel Wallace from Mt. Peninjau, many of which are currently unflagged and neglected by taxonomists in the NHMUK collections.

### ﻿Acknowledgments

The early work for this revision was carried out while MS was preparing for and subsequently recovering from an operation for cancer. Steven Hurel detected the cancer and Declan Cahill led the team which successfully operated to remove it. Without their skill and expertise this paper may well not have been written. Professor Houhun Li (Nankai University, China) and Rob de Vos (NBC) kindly assisted us with types not held at the NHMUK and, together with Alexandr Lvovsky, provided us with images of these types included in this paper. Ulf Eitschberger, the editor of Atalanta, kindly gave us permission to include in this paper images of drawings by Alexandr Lvovsky (Lvovsky 2014). Dr Roger Kendrick made available the habitus photographs which have been included. We would also like to thank Ian McMillan for his encouragement for this project, valuable insights based on his knowledge of the Australian xyloryctids and making available to us a substantial amount of his unpublished writing on the Australian Xyloryctidae. Henry Barlow has provided us with a number of recent specimens from his Genting Tea Estate in Peninsular Malaysia. Dr Mihai Stanescu (Bucharest, Romania) conducted a further search for the lost type of *Topirissalva* and provided us with information from unpublished materials from Popescu-Gorj. The next generation sequencing for this project was generously made available by Peter Buchner (Vienna, Austria). We would also like to thank the team at BOLD for performing the Sanger sequencing on our material. The molecular work on the type of *T.candidella* was supported via internal funds at the Natural History Museum. We are indebted to Joana Cristovao who helped with sample processing and Andie Hall who helped with lab work on this specimen. We thank our colleague Geoff Martin for practical help. We have also benefitted greatly from the wisdom and knowledge of our colleague Dr. Klaus Sattler. Jim Hayden and Ga Eun Lee helped greatly with getting some relatively old cladistic programs to run. Liz Milla (NCMI, Black Mountain, Australia) is thanked for suggesting MITObim for assembling data from museum specimens. Lastly, we owe a substantial debt of gratitude to Professor Li and to Dr. Mark Metz of the Smithsonian Institute, respectively the reviewer and subject editor for this paper, for their guidance on an earlier draft on which this paper is based and for their thorough review of and helpful input into this paper.

## Supplementary Material

XML Treatment for Topiris

XML Treatment for Topiris candidella

XML Treatment for Topiris cinderella

XML Treatment for Topiris ochrotincta

XML Treatment for Topiris schneeweissella

XML Treatment for Topiris sericella

XML Treatment for Topiris salva

XML Treatment for Topiris albogrisella

XML Treatment for Topiris lacteella

XML Treatment for Topiris madonna

XML Treatment for Topiris meyricki

XML Treatment for Topiris sampitella

XML Treatment for Topiris thunbergella

XML Treatment for Topiris albidella

XML Treatment for Topiris digiticosta

XML Treatment for Topiris

XML Treatment for Athrypsiastis

XML Treatment for Athrypsiastis phaeoleuca

XML Treatment for Athrypsiastis cheesmanae

XML Treatment for Athrypsiastis edelweissella

XML Treatment for Athrypsiastis halmaherella

XML Treatment for Athrypsiastis penumbrella

XML Treatment for Athrypsiastias symmetra

XML Treatment for Paralecta rosiflora

## ﻿Additional information

### Author ORCIDs

Mark J. Sterling https://orcid.org/0009-0006-5118-2838

Ben W. Price https://orcid.org/0000-0001-5497-4087

David C. Lees https://orcid.org/0000-0002-7580-8560

### Data availability

All of the data that support the findings of this study are available in the main text or Supplementary Information.

## Supplementary materials

Supplementary material 1 Data matrix Data type xls Explanation note Morphology and DNA barcodes. https://binary.pensoft.net/file/1269576This dataset is made available under the Open Database License (http://opendatacommons.org/licenses/odbl/1.0/). The Open Database License (ODbL) is a license agreement intended to allow users to freely share, modify, and use this Dataset while maintaining this same freedom for others, provided that the original source and author(s) are credited.Mark J. Sterling, Ben W. Price, David C. Lees

Supplementary material 2 Vouchers and accession numbers Data type xls Explanation note Genomic. https://binary.pensoft.net/file/1269577This dataset is made available under the Open Database License (http://opendatacommons.org/licenses/odbl/1.0/). The Open Database License (ODbL) is a license agreement intended to allow users to freely share, modify, and use this Dataset while maintaining this same freedom for others, provided that the original source and author(s) are credited.Mark J. Sterling, Ben W. Price, David C. Lees
